# Driftfusion: an open source code for simulating ordered semiconductor devices with mixed ionic-electronic conducting materials in one dimension

**DOI:** 10.1007/s10825-021-01827-z

**Published:** 2022-05-25

**Authors:** Philip Calado, Ilario Gelmetti, Benjamin Hilton, Mohammed Azzouzi, Jenny Nelson, Piers R. F. Barnes

**Affiliations:** 1 Department of Physics, Imperial College London, London, SW7 2AZ UK; 2 Institute of Chemical Research of Catalonia (ICIQ), Barcelona Institute of Science and Technology (BIST), Avda. Paisos Catalans 16, 43007 Tarragona, Spain

**Keywords:** Semiconductor device simulation, Numerical modelling, Drift-diffusion, Solar cells, Perovskites, Ionic-electronic conductors, Device physics

## Abstract

The recent emergence of lead-halide perovskites as active layer materials for thin film semiconductor devices including solar cells, light emitting diodes, and memristors has motivated the development of several new drift-diffusion models that include the effects of both electronic and mobile ionic charge carriers. In this work we introduce Driftfusion, a versatile simulation tool built for modelling one-dimensional ordered semiconductor devices with mixed ionic-electronic conducting layers. Driftfusion enables users to model devices with multiple, distinct, material layers using up to four charge carrier species: electrons and holes plus up to two ionic species. The time-dependent carrier continuity equations are coupled to Poisson’s equation enabling transient optoelectronic device measurement protocols to be simulated. In addition to material and device-wide properties, users have direct access to adapt the physical models for carrier transport, generation and recombination. Furthermore, a discrete interlayer interface approach circumvents the requirement for boundary conditions at material interfaces and enables interface-specific properties to be introduced.

**Supplementary Information:**

The online version contains supplementary material available at 10.1007/s10825-021-01827-z.

## Introduction

Accurate models of semiconductor devices are essential to further our understanding of the key physical processes governing these systems and hence rationally optimise them. One approach to modelling devices on the mesoscopic scale is to use continuum mechanics, whereby charge carriers are treated as continuous media as opposed to discrete particles. Typically, electronic carriers are modelled at discrete energy levels with a transport model describing the dynamics of carriers in response to an electric field (drift) and carrier density gradients (diffusion). This drift-diffusion (Poisson–Nernst–Planck) treatment leads to a system of coupled partial differential equations (the van Roosbroeck system [1]): a set of continuity equations, defining how the density of each charge carrier changes with time at each spatial location, are coupled with Poisson’s equation (Gauss’ Law), which relates the space-charge density to the electrostatic potential. For many architectures of thin-film semiconductor device (with the notable exception of transistors), provided that the materials are homogeneous and isotropic, it is sufficient to model devices with properties that vary in a single spatial dimension. In all but the most elementary of cases the resulting system of equations must be solved numerically.

### Recent progress in mixed electronic-ionic conductor device models

The recent emergence of lead-halide perovskites (referred to herein as perovskites) as active layer materials for thin film semiconductor devices including solar cells, light emitting diodes (LEDs), and memristors has motivated the development of several new drift-diffusion models that include mobile ionic species in addition to electronic carriers [2–10]. Ab initio calculations and experimental evidence have shown that, under many standard operating conditions, the charge density distribution, and consequently the electric field, in perovskite materials is dominated by high densities of relatively slow-moving mobile ionic defects [11–14]. This has a profound impact on the optoelectronic response of devices with perovskite active layers, leading to strong hysteresis effects in experimental measurements on timescales from microseconds to hundreds-of-seconds [15, 16].

To date, both experimental and theoretical research into perovskites has primarily focussed on their application as a photovoltaic absorber material for solar cells and we now review the recent advances in device-level modelling in this field of application. Van Reenen, Kemerink and Snaith were the first to publish perovskite solar cell (PSC) simulations using a coupled model that included continuity equations for three charge carriers: electrons, holes and a single mobile ionic species [3]. They found that current–voltage (J-V) hysteresis in PSCs could only be reproduced by including a density of trap states close to one of the interfaces acting as a recombination centre [3]. Later calculations of a 1.5 nm Debye length^1^ [4] suggested, however, that the choice of a 4 nm mesh spacing in the simulations was too coarse to properly resolve the ionic charge profiles at the perovskite active layer-transport layer interfaces (described herein simply as interfaces). Richardson and co-workers overcame the numerical challenge of high ionic carrier and potential gradients at the interfaces by using an asymptotic analytical model to calculate the potential drop in the Debye layers of a single mixed electronic-ionic conducting material layer [2, 4]. While this approach enabled the reproduction of hysteresis effects using high rates of bulk recombination, the inability to accurately model interfacial recombination limited the degree to which the simulation could represent real-world devices [4]. In a later publication by the same group modelling dark current transients, interfacial recombination was implemented, but only at the inner boundary of the Debye layer [17]. Furthermore, since these models were limited to a single layer, unrealistically large ionic charge densities were calculated at the interfaces as compared to three-layer models with discrete electron and hole transport layers (ETL and HTL, respectively).

Our own work simulating PSCs began with a three-layer p-i-n dual homojunction model in which the p- and n-type regions simulated the HTL and ETL, and where interfacial recombination was approximated by including high rates of recombination throughout these layers. Our results supported van Reenen et al.’s conclusion that both mobile ions and high rates of interfacial recombination are required to reproduce J-V hysteresis effects and other comparatively slow transient optoelectronic phenomena in p-i-n solar cells [18]. Shortly after, Neukom et al. published a modelling study with similar conclusions [5]. They used the commercial package SETFOS [19] to solve for electronic carriers in combination with a separate MATLAB code that solved for the ionic carrier distributions. More recently, Courtier et al. published results from IonMonger, a freely available, fully coupled, three-layer device model that included a single ionic charge carrying species and boundary conditions at the interfaces such that surface recombination of electronic carriers between the different layers could be explicitly included [8, 20]. There remained some limitations with the model however; only the majority carriers were calculated in the ETL and HTL, excluding the possibility of simulating single carrier devices, and intrinsic or low-doped transport layers such as organic semiconductors; ions were confined to the perovskite layer and; users only had the possibility to simulate three-layer devices. Jacobs et al. also published results from a three-layer coupled electronic-ionic carrier simulation implemented using COMSOL Multiphyics®[21] and MATLAB Livelink™[22, 23]. Most recently, Tessler and Vaynzof published impressive results from a similar three-layer PSC device model that included the option to use either Boltzmann or Fermi-Dirac statistics [24]. Notwithstanding, the methodological details from both Jacobs et al. [23] and Tessler and Vaynzof [24] are sparse and at the time of writing neither code is publicly available.

### Driftfusion

Here, we present a comprehensive guide to Driftfusion, our open source simulation tool designed for simulating semiconductor devices with mixed ionic-electronic conducting layers in one dimension. The software (based in MATLAB) enables users to simulate devices with any number of distinct material layers and up to four charge carrying species: electrons and holes by default plus up to two mobile ionic species. The time-dependent continuity equations are fully coupled to Poisson’s equation enabling transient optoelectronic measurements to be accurately simulated. In addition to common material parameters, users have direct access to adapt the carrier transport, recombination and generation models as well as the system’s initial and boundary conditions [25]. Driftfusion uses a discrete interlayer interface approach for junctions between material layers (heterojunctions) such that energetic and carrier density properties are graded between adjacent layers using a range of grading options. This method has the added benefits that it circumvents the requirement for boundary conditions at heterojunctions, and enables interface-specific properties to be defined within the interface regions. While the example architectures and outputs given in this work use PSCs as a model system, Driftfusion can, in principle, be used to model any ordered one-dimensional mixed ionic-electronic semiconductor or redox system for which the drift-diffusion approach is valid.

This work is divided into four main sections; we begin with a general overview of the simulation tool in Sect. 2; in Sect. 3 the default physical models for charge carrier transport, generation, and recombination are outlined; Sect. 4 provides a detailed description of the system architecture and a step-by-step guide of the important commands and functions that will enable readers to get started with Driftfusion; we conclude in Sect. 5 by comparing calculations from Driftfusion to two analytical and two numerical models to validate the simulation solutions and the discrete interface approach.

## General overview of Driftfusion

### Workflow

**Fig. 1:**
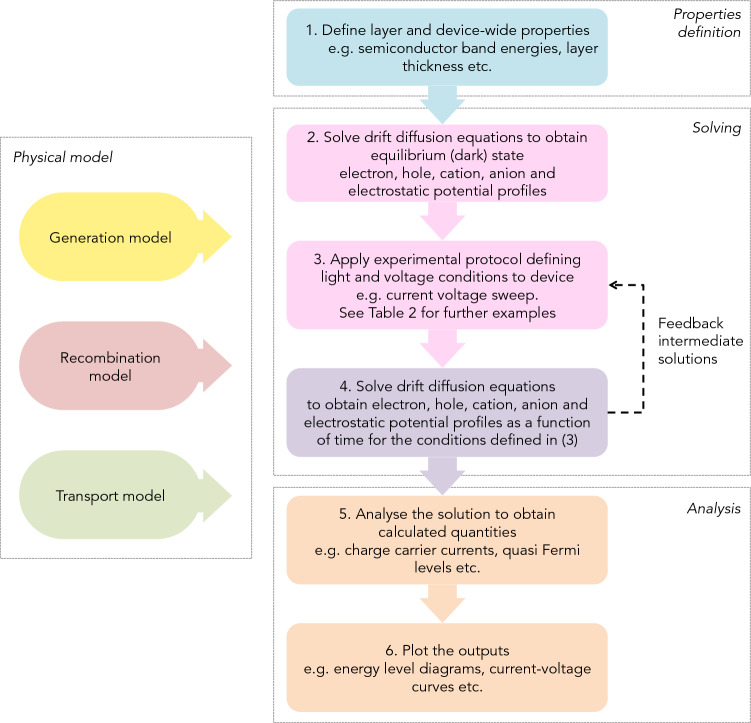
The general workflow of Driftfusion. 1. The user defines a device in the properties definition step; 2. The device equilibrium state is solved for using the given recombination and transport models; 3. An experimental protocol, which defines time-dependent voltage and optical generation conditions, is applied to the equilibrium solution; 4. A solution is obtained that may be fed back into the protocol until the desired solution is reached; 5. The solution is analysed to obtain calculated outputs; 6. The outputs are visualised using plotting tools

A flow diagram summarising Driftfusion’s general workflow is given in Fig. 1. The system is designed such that the user performs a linear series of steps to obtain a solution; (1) the process begins with the creation of a semiconductor device object for which both device-wide properties, such as the system temperature, and layer-specific properties, such as the carrier mobilities are defined. A user-definable physical model comprised of one-dimensional generation, recombination, and transport models determines the continuity equation for each charge carrier (see Sect. 3 for the default expressions); (2) the continuity equations are solved simultaneously with Poisson’s equation (Eq. 17) to obtain a solution for the electron density, hole density, cation density (optional), anion density (optional), and electrostatic potential distributions at equilibrium; (3) an experimental protocol such as a current–voltage scan is defined using an appropriate set of input parameters e.g. scan rate and voltage limits. The protocol generates time-dependent voltage and light conditions that are subsequently applied to the device, typically using the equilibrium solution as the initial conditions. In more sophisticated protocols the solution is broken into a series of steps whereby intermediate solutions are fed back into the solver. Likewise, protocols can be cascaded such that the solution from one protocol supplies the initial conditions for the next; (4) the desired solution is output as a MATLAB data structure (see *Solution structures* highlighted box); (5) the solution structure can be analysed to obtain calculated outputs such as the charge carrier currents, quasi-Fermi levels, etc.; (6) a multitude of plotting tools are available to visualise the simulation outputs. Instructions on how to run each step programmatically and further details of the system architecture, protocols, solutions, and analysis functions are given in Sect. 4.

### Licensing information

The front end code of Driftfusion has been made open-source under the GNU Affero General Public License v3.0 in order to accelerate the rate of development and expand our collective knowledge of mixed ionic-electronic conducting devices [26]. It is important to note, however, that Driftfusion currently uses MATLAB’s Partial Differential Equation solver for Parabolic and Elliptic equations (pdepe), licensed under the MathWorks, Inc. Software License Agreement, which strictly prohibits modification and distribution. If you use Driftfusion please consider giving back to the project by providing feedback and/or contributing to its continued development and dissemination.

We now proceed to describe the default physical models underlying this release of Driftfusion  [26]. Herein relevant Driftfusion functions and commands are highlighted using boxes and referred to using command line typeface.

**Figure:**
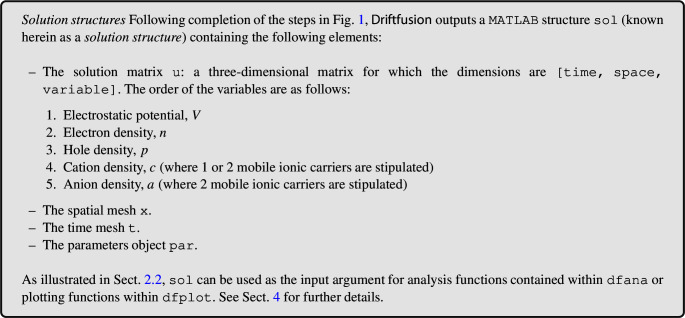

## Implementation of established semiconductor theoretical principles in Driftfusion

The device physics implemented in Driftfusion is principally based on established semi-classical semiconductor transport and continuity principles, which are well described in Sze and Kwok [27] and Nelson [28]. Elements of this section are adapted from Ref. [29] and are provided here as a direct reference for the reader. The equations described herein are written in terms of a single spatial dimension and can only be applied to devices with one-dimensional architectures and for which the material layers are homogeneous.

Driftfusion evolved from a diffusion-only code written to simulate transient processes in dye sensitised solar cells [30] and uses MATLAB’s [22] built-in pdepe solver [31]. The code solves the continuity equations and Poisson’s equation for electron density *n*, hole density *p*, cation density *c* (optional), anion density *a* (optional), and the electrostatic potential *V* as a function of position *x*, and time *t*.

The full details of the numerical methods employed by the pdepe solver for discretising the equations are given in Skeel and Berlizns 1990 [32].

### Semiconductor energy levels

**Fig. 2:**
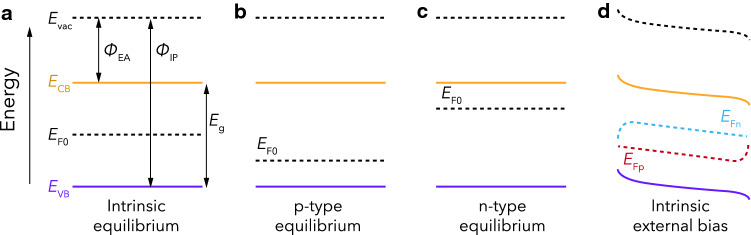
Semiconductor energy levels. **a** An intrinsic semiconductor material showing the vacuum level $E_\mathrm {vac}$, electron affinity $\varPhi _\mathrm {EA}$, ionisation potential $\varPhi _\mathrm {IP}$, conduction and valence band energies $E_\mathrm {CB}$ and $E_\mathrm {VB}$, band gap $E_\mathrm {g}$, equilibrium Fermi energy $E_\mathrm {F0}$ and electron and hole quasi-Fermi levels $E_\mathrm {Fn}$ and $E_\mathrm {Fp}$. **b** A *p*-type material: $E_\mathrm {F0}$ lies closer to the VB due to acceptor impurities adding holes to the VB. **c** An *n*-type material: $E_\mathrm {F0}$ lies closer to the CB as donor impurities increase the CB electron density. **d** An intrinsic layer under external bias

Figure 2a shows the energy levels associated with an idealised intrinsic semiconductor. The electron affinity $\varPhi _\mathrm {EA}$ and ionisation potential $\varPhi _\mathrm {IP}$ are the energies required to add an electron to the conduction band (CB) from the vacuum level $E_\mathrm {vac}$ and to remove an electron from the valence band (VB) to $E_\mathrm {vac}$  respectively. Note that in contrast to the established convention and the description given here, in Driftfusion $\varPhi _\mathrm {EA}$ and $\varPhi _\mathrm {IP}$ are input and stored as negative values for consistency with other energetic properties referenced using the electron energy scale. The electronic band gap $E_\mathrm {g}$ of the material can be defined as

1 $$\begin{aligned} E_\mathrm {g} = \varPhi _\mathrm {IP} - \varPhi _\mathrm {EA}. \end{aligned}$$

#### The vacuum energy

The vacuum energy $E_\mathrm {vac}$ is defined as the energy at which an electron is free of all forces from a solid [28]. Spatial changes in the electrostatic potential *V* are therefore reflected in $E_\mathrm {vac}$ such that, at any point in space,

2 $$\begin{aligned} E_{\mathrm {vac}}(x,t) = -qV(x,t) , \end{aligned}$$

where *q* is the elementary charge.

#### Conduction and valence band energies

The conduction and valence band energies $E_{\mathrm {CB}}$ and $E_{\mathrm {VB}}$ are defined as the difference between the vacuum energy and $\varPhi _\mathrm {EA}$ and $\varPhi _\mathrm {IP}$, respectively, such that

3 $$\begin{aligned} E_{\mathrm {CB}}(x,t)= & {} E_{\mathrm {vac}}(x,t)-\varPhi _\mathrm {EA}(x), \end{aligned}$$

4 $$\begin{aligned} E_{\mathrm {VB}}(x,t)= & {} E_{\mathrm {vac}}(x,t)-\varPhi _\mathrm {IP}(x). \end{aligned}$$

The band energies, then, include both the energy associated with the molecular orbitals of the solid and the electrostatic potential arising from the existence of charge both within and external to the material.

### Electronic carrier densities and quasi-Fermi levels

#### The occupation probability distribution function and electronic equilibrium carrier densities

At equilibrium the net exchange of mass and energy into and out of, as well as between different locations within a system is zero. Under these conditions the average probability *f* that an electron will occupy a particular state of energy *E* at equilibrium in a semiconductor at temperature *T* is given by the Fermi-Dirac (F-D) distribution function,

5 $$\begin{aligned} f(E, E_\mathrm {F0}, T) = \left( e^{\frac{E - E_\mathrm {F0}}{k_{\mathrm {B}} T}} + 1 \right) ^{-1} , \end{aligned}$$

where $k_\mathrm {B}$ is Boltzmann’s constant. The equilibrium Fermi energy $E_\mathrm {F0}$ defines the energy at which a hypothetical electronic state has a 50% probability of occupation. At equilibrium, and for V=0, the Fermi energy is identical to the chemical potential of the material. For an intrinsic semiconductor, $E_\mathrm {F0}$ lies close to the middle of the gap. Where the semiconductor is p-type, dopant atoms accept electrons from the bands, shifting $E_\mathrm {F0}$ towards the valence band (Fig. 2b). Similarly, where the semiconductor is n-type, dopants donate electrons to the bands, shifting $E_\mathrm {F0}$ towards the conduction band (Fig. 2c). Note that herein the subscript ‘0’ denotes the value or expression of properties at equilibrium e.g. $E_{CB,0}$ is the conduction band energy at equilibrium.^2^

To obtain the density of free electrons in the conduction band *n*, the product of the probability distribution function and the conduction band density of states (DOS) function $g_{CB}$ is integrated across energies above the conduction band edge $E_{CB}$:

6 $$\begin{aligned} n_0 = \int _{E_\mathrm {CB,0}}^{\infty } g_\mathrm {CB}(E) f(E, E_\mathrm {F0}, T) dE. \end{aligned}$$

Similarly, to obtain the density of free holes in the valence band *p*, the product of the average probability that an electron is *not* at energy *E* (i.e. (1-f)) with the valence band DOS function $g_\mathrm {VB}$ is integrated across all energies up to the valence band edge $E_\mathrm {VB}$:

7 $$\begin{aligned} p_0 = \int _{-\infty }^{E_\mathrm {VB,0}} g_\mathrm {VB}(E) (1 - f(E, E_\mathrm {F0}, T)) dE. \end{aligned}$$

For semiconductor materials, $g_\mathrm {CB}$ and $g_\mathrm {VB}$ are typically modelled as parabolic functions with respect to electron energy at energies close to the band edges. Specifically, $g_\mathrm {CB} = 4 \pi (2m_e^*/h^2)^{3/2}(E - E_\mathrm {CB})$ and $g_\mathrm {VB} = 4 \pi (2m_h^*/h^2)^{3/2}(E_\mathrm {VB} - E)$, where $m_e^*$ and $m_h^*$ are the effective electron and hole masses and *h* is Planck’s constant. Unfortunately, closed-form solutions cannot be found to Eqs. 6 and 7 using the parabolic band approximation and the F-D distribution function (Eq. 5). Hence, we use Blakemore’s approximation [33] to the above integrals to obtain closed-form expressions for the equilibrium carrier densities:

8 $$\begin{aligned} n_0(x)= & {} N_\mathrm {CB}(x) \left( e^{\frac{E_\mathrm {CB,0}(x) - E_\mathrm {F0}(x)}{k_{\mathrm {B}} T}} + \gamma \right) ^{-1}, \end{aligned}$$

9 $$\begin{aligned} p_0(x)= & {} N_\mathrm {VB}(x) \left( e^{\frac{E_\mathrm {F0}(x) - E_\mathrm {VB,0}(x)}{k_{\mathrm {B}} T}} + \gamma \right) ^{-1}, \end{aligned}$$

where $N_\mathrm {CB}$ and $N_\mathrm {VB}$ are the temperature-dependent^3^ effective density of states (eDOS)^4^ of the conduction and valence bands, respectively. γ is a constant defining how close the approximation is to the Boltzmann regime (note that Eqs. 8 and 9 reduce to the Boltzmann approximation for γ=0). Following Farrell et al., we set γ=0.27 by default for ordered materials [34]. This results in a close agreement to F-D statistics for $E_\mathrm {VB} - 1.3 k_\mathrm {B} T< E_\mathrm {F0} < E_\mathrm {CB} + 1.3 k_\mathrm {B} T$, such that degenerate semiconductor states are permissible within the scope of the model.

#### Equilibrium Fermi levels in doped materials

In charge-neutral n-type materials the equilibrium electron density is approximately equal to the density of donor dopant atoms such that $n_0 \approx N_\mathrm {D}$. Similarly in p-type materials, $p_0 \approx N_\mathrm {A}$, where $N_\mathrm {A}$ is the density of acceptor dopants. In Driftfusion users input values for $E_{\mathrm {F0}}$ for each material layer and the corresponding equilibrium carrier and doping densities, $n_0$, $p_0$, $N_\mathrm {D}$, and $N_\mathrm {A}$ are calculated during creation of the device parameters object (see Sect. 4.2) according to Eqs. 8 and 9.

**Figure:**
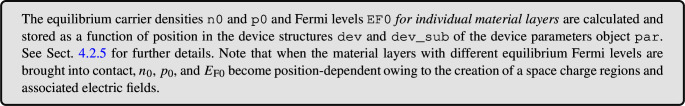

#### Quasi-Fermi levels

A key approximation in semiconductor physics is the assumption that, under the application of an external optical or electrical bias, the electron and hole populations at any particular location can be treated separately, with individual distribution functions and associated quasi-Fermi levels (QFLs), $E_\mathrm {Fn}$ and $E_\mathrm {Fp}$ (Fig. 2d). This is permitted because the thermal relaxation of carriers to the band edges is typically significantly faster than interband relaxation, resulting in quasi-equilibrium states for each carrier population [28]. Under these circumstances a similar approach to that taken for the true equilibrium state can be used to derive expressions for the QFLs,

10 $$\begin{aligned} E_{\mathrm {Fn}}(x,t)= & {} E_{\mathrm {CB}}(x,t)+k_\mathrm {B}T \ln \left( \dfrac{n(x,t)}{N_{\mathrm {CB}}(x)}\ - \gamma \right) , \end{aligned}$$

11 $$\begin{aligned} E_{\mathrm {Fp}}(x,t)= & {} E_{\mathrm {VB}}(x,t)-k_\mathrm {B}T \ln \left( \dfrac{p(x,t)}{N_{\mathrm {VB}}(x)}\ - \gamma \right) . \end{aligned}$$

It can be helpful to conceptualise the QFLs as the sum of the electrostatic (-V) and average chemical potential energy ($k_\mathrm {B}T\ln (n/N_\mathrm {CB} - \gamma ) - \varPhi _\mathrm {EA}$ for electrons, $-k_\mathrm {B}T\ln (p/N_\mathrm {VB} - \gamma ) - \varPhi _\mathrm {IP}$ for holes) components of the carriers at each location. It follows that the gradient of the QFLs provides a convenient way to determine the direction of the current since, from the perspective of the electron energy scale, electrons move ‘downhill’, and holes move ‘uphill’ in response to electrochemical potential gradients. Moreover, the electron and hole currents, $J_n$ and $J_p$, can be expressed in terms of the product of the electron and hole QFL gradients with their corresponding carrier conductivities, $\sigma _n$ and $\sigma _p$, such that

12 $$\begin{aligned} J_n(x,t)= & {} \dfrac{\sigma _n}{q} {\frac{{\text {d}}E_{\mathrm {Fn}}(x,t)}{{\text {d}}x}}, \end{aligned}$$

13 $$\begin{aligned} J_p(x,t)= & {} \dfrac{\sigma _p}{q} {\frac{{\text {d}}E_{\mathrm {Fp}}(x,t)}{{\text {d}}x}}. \end{aligned}$$

Here, the conductivities are the product of the electronic carrier mobilities, $\mu _{n}$ and $\mu _{p}$, with their corresponding concentrations and the elementary charge:

14 $$\begin{aligned} \sigma _n(x,t)= & {} qn(x,t)\mu _n(x), \end{aligned}$$

15 $$\begin{aligned} \sigma _p(x,t)= & {} qp(x,t)\mu _p(x). \end{aligned}$$

**Figure:**
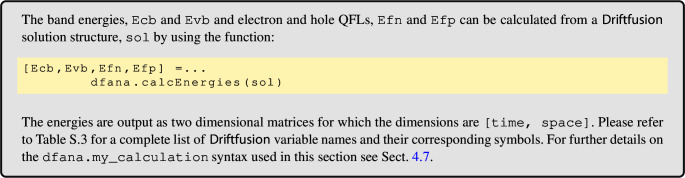

#### Open circuit voltage

The open circuit voltage $V_\mathrm {OC}$ is the maximum energy per unit charge that can be extracted from an electrochemical cell for a given charge state at open circuit. The $V_\mathrm {OC}$ can be calculated using the difference in the electron QFL at the location of the cathode ($x_\mathrm {cathode}$) and the hole QFL at the location of the anode ($x_\mathrm {anode}$) with the cell at open circuit,

16 $$\begin{aligned} qV_{\mathrm {OC}}(t) = E_{\mathrm {Fn}}(x_\mathrm {cathode}, t) - E_{\mathrm {Fp}}(x_\mathrm {anode}, t) . \end{aligned}$$

**Figure:**
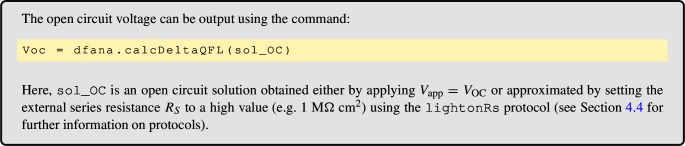

### Poisson’s equation

Poisson’s equation (deriving from Gauss’s Law) relates the electrostatic potential to the space charge density ρ and the relative dielectric constant of the medium $\varepsilon _r$ via the Divergence Theorem. The space charge density is the sum of the mobile and static charge densities at each spatial location. Doping is simulated via the inclusion of fixed charge density terms for ionising donor and acceptor atoms. In this release of Driftfusion mobile ionic carriers are modelled as Schottky defects [35] for which every ion has an oppositely charged counterpart, maintaining overall ionic defect charge neutrality within the device.^5^ The mobile cation density *c* is initially balanced by a uniform static counter-ion density $N_\mathrm {cat}$ and the mobile anion density *a* is similarly balanced by a static density $N_\mathrm {ani}$. For the one-dimensional system described, Poisson’s equation can be explicitly stated as

17 $$\begin{aligned}&\dfrac{\partial ^2 V(x,t)}{\partial x^2}= -\dfrac{\rho (x,t)}{\varepsilon _0\varepsilon _r(x)} \nonumber \\&\quad = -\dfrac{q}{\varepsilon _0\varepsilon _r(x)}(p(x,t) - n(x,t) + N_{\mathrm {D}}(x) - N_{\mathrm {A}}(x) +...\nonumber \\&\quad z_c c(x,t) + z_a a(x,t) - z_c N_\mathrm {cat}(x) - z_a N_\mathrm {ani}(x)), \end{aligned}$$

where $\varepsilon _0$ is the permittivity of free space. We emphasise that *p*, *n*, *c*, and *a* represent mobile species, while $N_{\mathrm {A}}$, $N_{\mathrm {D}}$, $N_\mathrm {cat}$ and $N_\mathrm {ani}$ are static ion densities. $z_c$ and $z_a$ are the integer charge states for the ionic species (by default $z_c=1$, and $z_a=-1$).

**Figure:**
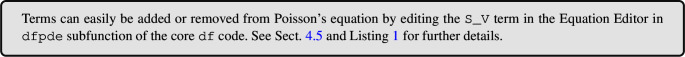

**Figure:**
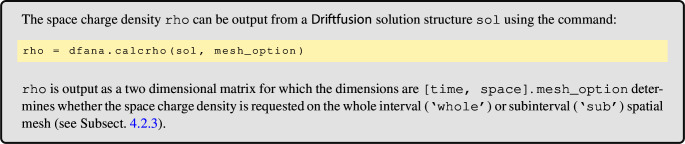

### Charge transport: Drift and diffusion

As the name suggests, the drift-diffusion (Poisson–Nernst–Planck) model assumes that charge transport within semiconductors is driven by two processes:

1. *Drift* arising from the Lorentz force on charges due to an electric field *F*, where F=-dV/dx.
2. *Diffusion* arising from the entropic drive for carriers to move from regions of high to low concentration.

#### Bulk transport

Within the bulk of material layers the expressions for the flux density of electrons $j_n$, holes $j_p$, anions $j_a$, and cations $j_c$ with mobility $\mu _{y}$ and diffusion coefficient $D_{y}$ (where *y* denotes a generic charge carrier) are given by

18 $$\begin{aligned} j_n(x,t)= & {} -\mu _n(x)n(x,t)F(x,t) - D_n(n,x) \dfrac{\partial n(x,t)}{\partial x}, \end{aligned}$$

19 $$\begin{aligned} j_p(x,t)= & {} \mu _p(x)p(x,t)F(x,t) - D_p(p,x) \dfrac{\partial p(x,t)}{\partial x}, \end{aligned}$$

20 $$\begin{aligned} j_c(x,t)= & {} \mu _c(x) z_c c(x,t)F(x,t) - D_c(c,x) \dfrac{\partial c(x,t)}{\partial x}, \end{aligned}$$

21 $$\begin{aligned} j_a(x,t)= & {} \mu _a(x) z_a a(x,t)F(x,t) - D_a(a,x) \dfrac{\partial a(x,t)}{\partial x}.\nonumber \\ \end{aligned}$$

Figure S.1 illustrates how the directions of electron and hole flux densities are determined from gradients in the electric potential and charge carrier densities. An analogous diagram can be drawn for mobile ionic species by substituting cations for holes and anions for electrons. The carrier (particle) currents are calculated as the product of the flux densities with the specific carrier charge $q z_y$ such that $J_y = q z_y j_y$.

**Figure:**
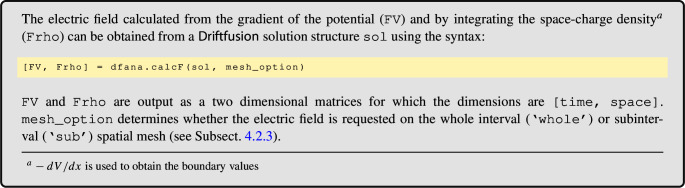

#### Diffusion enhancement

The implementation of electronic carrier statistics beyond the Boltzmann approximation (Sect. 3.2.1) necessitates the inclusion of a generalised Einstein relation to define the relationship between the carrier mobilities and diffusion coefficients as a function of band state occupancy [34]. The result is a nonlinear diffusion enhancement as the QFLs approach and move into the bands. Under Blakemore’s approximation, [33] the diffusion coefficient-mobility relationships for electrons and holes can be expressed using the closed-forms

22 $$\begin{aligned} D_n(n, x)= & {} \dfrac{k_\mathrm {B}T}{q}\mu _n(x)\left( \dfrac{N_\mathrm {CB}(x)}{N_\mathrm {CB}(x) - \gamma n(x,t)} \right) , \end{aligned}$$

23 $$\begin{aligned} D_p(p, x)= & {} \dfrac{k_\mathrm {B}T}{q}\mu _p(x)\left( \dfrac{N_\mathrm {VB}(x)}{N_\mathrm {VB}(x) - \gamma p(x,t)} \right) . \end{aligned}$$

We use similar expressions to those above for the ionic carriers from a model proposed by Kilic et al. [36] to account for steric effects at high ion densities:

24 $$\begin{aligned} D_c(c, x)= & {} \dfrac{k_\mathrm {B}T}{q}\mu _c(x)\left( \dfrac{c_{max}(x)}{c_{max}(x) - c(x,t)} \right) , \end{aligned}$$

25 $$\begin{aligned} D_a(a, x)= & {} \dfrac{k_\mathrm {B}T}{q}\mu _a(x)\left( \dfrac{a_{max}(x)}{a_{max}(x) - a(x,t)} \right) . \end{aligned}$$

Here, $a_{max}$ and $c_{max}$ denote the limiting anion and cation densities. In the first instance these are set to the lattice cite density for the corresponding ions.

#### Transport across heterojunctions

At the interface between two different semiconductor materials there is a change in the band energies and electronic density of states. In Driftfusion we choose to model the mixing of states at the interface using a smooth transition in material properties over a discrete interlayer region, in contrast to the commonly employed abrupt interface model^6^ (see Fig. 4 for a schematic illustrating the difference between the two models). To accommodate this approach, Eqs. 18 and 19 are modified to include additional gradient terms for spatial changes in $\varPhi _\mathrm {EA}$, $\varPhi _\mathrm {IP}$, $N_{\mathrm {CB}}$, and $N_{\mathrm {VB}}$. This leads to an adapted set of flux equations for electrons and holes within the interfaces [37]:

26 $$\begin{aligned} j_n(x,t)= & {} \mu _n(x,t) n\left( -F(x,t)-\dfrac{\partial \varPhi _\mathrm {EA}(x)}{\partial x} \right) \nonumber \\&- D_n(n,x) \left( \dfrac{\partial n(x,t)}{\partial x} - \dfrac{n(x,t)}{N_{\mathrm {CB}}(x)}\dfrac{\partial N_{\mathrm {CB}}(x)}{\partial x}\right) , \nonumber \\ \end{aligned}$$

27 $$\begin{aligned} j_p(x,t)= & {} \mu _p(x,t) p\left( F(x,t) + \dfrac{\partial \varPhi _\mathrm {IP}(x)}{\partial x} \right) \nonumber \\&- D_p(p,x) \left( \dfrac{\partial p(x,t)}{\partial x} - \dfrac{p(x,t)}{N_{\mathrm {VB}}(x)}\dfrac{\partial N_{\mathrm {VB}}(x)}{\partial x}\right) .\nonumber \\{} \end{aligned}$$

Values of between 1 and 2 nm have been extensively tested for the interfacial region thickness and are used in the example parameter files accompanying Driftfusion. By default, $\varPhi _\mathrm {EA}$ and $\varPhi _\mathrm {IP}$ are graded linearly, while $N_{\mathrm {CB}}$ and $N_{\mathrm {VB}}$ are graded exponentially within the interfacial regions.

**Figure:**
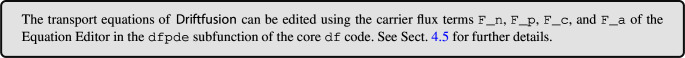

#### Displacement current

The displacement current $J_{disp}$, as established in the Maxwell-Ampere law, is the rate of change of the electric displacement field, $\partial D/ \partial t$. In terms of the electric field the displacement current can be expressed as

28 $$\begin{aligned} J_{disp}(x,t) = \varepsilon _0 \varepsilon _r(x) \dfrac{\partial F(x,t)}{\partial t}. \end{aligned}$$

#### Total current

The total current, *J* is the sum of the individual current components at each point in space and time such that

29 $$\begin{aligned} J(x,t) = J_n(x,t) + J_p(x,t) + J_a(x,t) + J_c(x,t) + J_{disp}(x,t) . \end{aligned}$$

**Figure:**
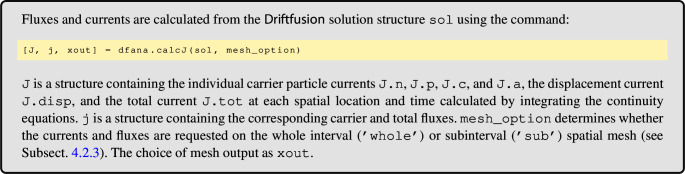

#### Validity criteria for the drift-diffusion approach

The drift-diffusion approach is valid for semiconductor materials that satisfy the following criteria [28]:

1. The electron and hole populations are at quasi-thermal equilibrium.
2. The electron and hole population temperatures are the same as that of the atomic lattice.
3. Changes in state occupancy are more likely to be due to scattering collisions within a band than generation and recombination events between bands or trapping events.
4. The electron and hole states can be described by a quantum number, *k*.
5. The mean free path length of carriers, $\bar{L}$ is significantly shorter than the layer thickness, *d* ($\bar{L}<< d$).

### Charge continuity

The continuity equations are a set of ‘book-keeping’ equations, based on the conservation of charge, describing how charge carrier densities change in time at each location within a system. In one-dimension, the continuity equation for a generic carrier *y* with flux density $j_y$, and source/sink term $S_y$ can be expressed as

30 $$\begin{aligned} {\frac{{\partial }y(x,t)}{{\partial }t}} = -\dfrac{\partial j_y(x,t)}{\partial x} + S_y(x,t). \end{aligned}$$

**Fig. 3:**
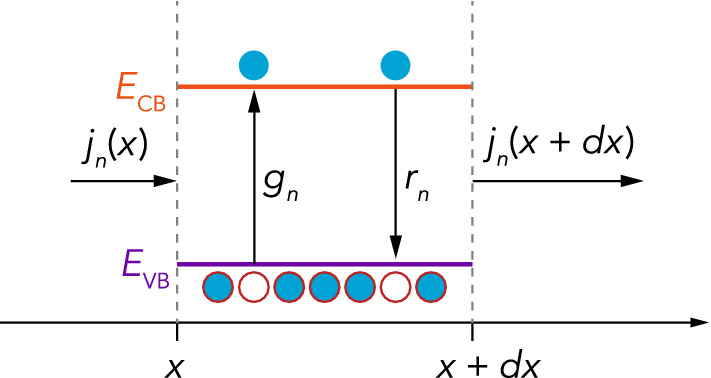
Continuity of electrons within a semiconductor. Schematic illustrating the principle of continuity for electrons in a thin slab of material *dx*. A difference in the incoming and outgoing flux density $j_n$, generation $g_n$, and recombination $r_n$ of electrons results in changes in the electron concentration over *dx* (Eq. 31). The conduction and valence band energies are denoted $E_{\mathrm {CB}}$ and $E_{\mathrm {VB}}$, respectively. Electrons are represented by solid blue circles and holes by open red circles. Figure concept adapted fromRef. [38]

For electronic carriers, *S* is composed of two components; 1. Generation *g* of carriers by both thermal and photo excitation and; 2. Recombination *r* of carriers through radiative (photon emission) and non-radiative pathways. Figure 3 illustrates the principle of continuity: changes in the electron concentration with time within a thin slab *dx* are determined by the generation, recombination, and difference in incoming and outgoing flux density of carriers.

Where chemical reactions take place within devices, additional generation and recombination terms for carriers may also contribute to *S*. In the current version of Driftfusion, mobile ionic charge carriers are treated as inert such that $g_{c} = g_{a} = r_{c} = r_{a} = 0$. Users are, however, free to edit the default source terms using the Equation Editor (Sect. 4.5). A guide describing how to do this is included in the Supplemental Information Section S.6.

In one-dimension the continuity equations for electrons, holes, cations and anions are given by

31 $$\begin{aligned}&\dfrac{\partial n(x,t)}{\partial t} = -\dfrac{\partial j_n(x,t)}{\partial x} + g_n(x,t) - r_n(x,t), \end{aligned}$$

32 $$\begin{aligned}&\dfrac{\partial p(x,t)}{\partial t} = -\dfrac{\partial j_p(x,t)}{\partial x} + g_p(x,t) - r_p(x,t), \end{aligned}$$

33 $$\begin{aligned}&\dfrac{\partial c(x,t)}{\partial t} = -\dfrac{\partial j_c(x,t)}{\partial x(x,t)} + g_c(x,t) - r_c(x,t), \end{aligned}$$

34 $$\begin{aligned}&\dfrac{\partial a(x,t)}{\partial t} = -\dfrac{\partial j_a(x,t)}{\partial x} + g_a(x,t) - r_a(x,t). \end{aligned}$$

Equation 17 and Eqs. 31–34 then form the complete set of equations to be solved.

#### Steady-state approximation to electronic carrier densities and fluxes within the interfacial regions

To better understand the discrete interface model employed by Driftfusion we solve the electron and hole continuity equations (Eqs. 26, 27, 31 and 32) to obtain analytical expressions for the electronic carrier densities within the discrete interfacial regions using the following approximations and assumptions:

1. Carriers within an interface are at steady-state with respect to the surroundings layers (dn/dt=0, dp/dt=0).
2. There is no optical generation within the interface (g=0).
3. The electric field can be treated as approximately constant throughout the interfacial region (dF/dx=0).
4. The recombination rate *r* within the interfacial region is constant and distributed uniformly.
5. The electron and hole QFLs remain within the Boltzmann regime ($E_\mathrm {CB} - E_\mathrm {Fn} > 3 k_\mathrm {B} T$ and $E_\mathrm {Fp} - E_\mathrm {VB} > 3k_\mathrm {B} T$)

**Fig. 4:**
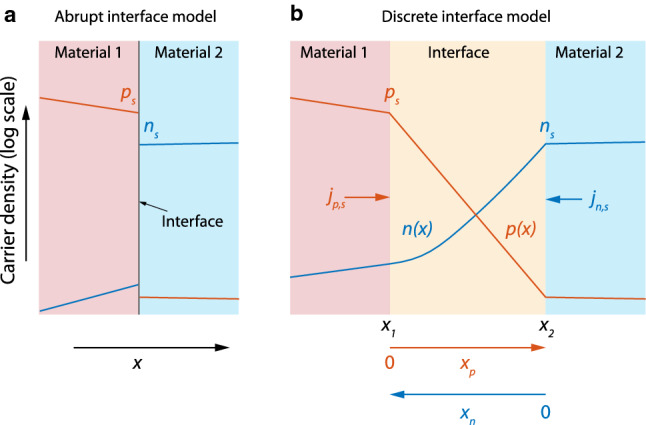
Schematic of carrier densities at (**a**) abrupt and (**b**) discrete interface models. $n_s$ and $p_s$ are the boundary electron and hole densities, while $j_{n,\mathrm {s}}$ and $j_{p,\mathrm {s}}$ are the boundary fluxes. The pure exponential change in hole density, *p*(*x*) across the interfacial region (**b**) implies the hole mobility is large such that the $j_{p,\mathrm {s}}$ and *r* terms in Eq. 36 are negligible. By contrast, the curvature in the logarithm of the electron density, *n*(*x*) profile indicates that the $j_{n,\mathrm {s}}$ and *r* terms in Equation 35 are of a similar order to the $n_s$ term close to $x_1$. The red, yellow and blue regions indicate Material 1 (p-type), Interface, and Material 2 (n-type) layers, respectively. $x_n$ and $x_p$ are the translated position (*x*) co-ordinates

As detailed in the Supplemental Information Section S.3.1, using the boundary conditions $n(x_n = 0) = n_\mathrm {s}$, $p(x_p = 0) = p_\mathrm {s}$, $j_n(x_n = 0) = j_{n,s}$, and $j_p(x_p = 0) = j_{p,s}$ (see Fig. 4b), the following expressions can be obtained for the carrier densities within the interfacial regions:

35 $$\begin{aligned} n(x_n)= & {} n_\mathrm {s} e^{\alpha x_n} + \dfrac{j_{n,s}}{k_B T \alpha \mu _n}(1-e^{\alpha x_n})-\nonumber \\&\dfrac{r}{k_B T \alpha ^2 \mu _n}(1 -e^{\alpha x_n} + \alpha x_n), \end{aligned}$$

36 $$\begin{aligned} p(x_p)= & {} p_\mathrm {s} e^{\beta x_p} + \dfrac{j_{p,s}}{k_B T \beta \mu _p}(1- e^{\beta x_p})-\nonumber \\&\dfrac{r}{k_B T \beta ^2 \mu _p}(1 -e^{\beta x_p} + \beta x_p), \end{aligned}$$

where,

37 $$\begin{aligned}&\alpha = -\frac{1}{k_\mathrm {B}T} \left( \dfrac{\partial \varPhi _\mathrm {EA}(x_n)}{\partial x_n} - q\dfrac{\partial V}{\partial x_n} \right) + \dfrac{1}{N_{\mathrm {CB}}(x_n)}\dfrac{\partial N_{\mathrm {CB}}(x_n)}{\partial x_n},\nonumber \\ \end{aligned}$$

38 $$\begin{aligned}&\beta = \frac{1}{k_\mathrm {B}T} \left( \dfrac{\partial \varPhi _\mathrm {IP}(x_p)}{\partial x_p} - q\dfrac{\partial V}{\partial x_p} \right) + \dfrac{1}{N_{\mathrm {VB}}(x_p)}\dfrac{\partial N_{\mathrm {VB}}(x_p)}{\partial x_p}.\nonumber \\ \end{aligned}$$

The corresponding fluxes are given by

39 $$\begin{aligned}&j_{n}(x_n) = j_{n,\mathrm {s}} - rx_n, \end{aligned}$$

40 $$\begin{aligned}&j_{p}(x_p) = j_{p,\mathrm {s}} - rx_p. \end{aligned}$$

As illustrated in Fig. 4b, the translated co-ordinates $x_n$ and $x_p$ are taken to be in the direction for which α and β are negative and typically the direction for which carrier densities decay.

Example solutions comparing the analytical approximations to numerical solutions calculated using Driftfusion under different transport and recombination regimes are given in the Supplemental Information, Section S.3.2. Where transport is a limiting factor within the interfaces the solutions become strongly dependent on the boundary flux and recombination rates. It is noteworthy however that in the limiting case of infinitely fast transport ($\mu _{n,p} \rightarrow \infty$) Equations 35 and 36 converge towards purely exponential forms for which the carrier densities change by a Boltzmann factor ($\varDelta n = N_\mathrm {CB} e^{\alpha d_\mathrm {int}}$ and $\varDelta p = N_\mathrm {VB} e^{\beta d_\mathrm {int}}$) across the width of the interface $d_\mathrm {int}$. For the special case where F=0, the result is a change in carrier densities equivalent to that expected from an abrupt interface model using Boltzmann statistics. The results presented in this section are applied in Sect. 3.7.3 to the interfacial volumetric surface recombination model.

### Electronic carrier generation

Two optical models for electronic carrier generation are currently available for use in Driftfusion; uniform generation for which a uniform volumetric generation rate $g_0$ is defined for each layer (excluding interfacial regions) and; Beer–Lambert law generation as described below. Irrespective of the choice of optical model the generation rate is zeroed within the interfacial regions to avoid potential stability issues.

#### Beer–Lambert law generation

The Beer–Lambert law models the photon flux density as falling exponentially within a material with a characteristic photon energy-dependent absorption coefficient $\alpha _{\mathrm {abs}}$. The volumetric generation rate *g*, over a range of photon energies $E_{\gamma }$ with incident photon flux density $\varphi _0$, is given by the integral across the spectrum,

41 $$\begin{aligned}&g(x) =(1-\kappa ) \int _0 ^\infty \alpha _{\mathrm {abs}}(E_{\gamma },x) \varphi _0 (E_{\gamma }) \exp (-\alpha _{\mathrm {abs}}(E_{\gamma },x) x) \ dE_{\gamma },\nonumber \\ \end{aligned}$$

where κ is the reflectance. For simplicity, we assume that a single electron-hole pair is generated by a single photon.

#### Arbitrary generation profiles

An arbitrary generation profile can be inserted following creation of the parameters object for users who wish to use profiles calculated from different models using an external software package. Details on how to do this are given in Sect. 4.2.7.

### Recombination

**Fig. 5:**
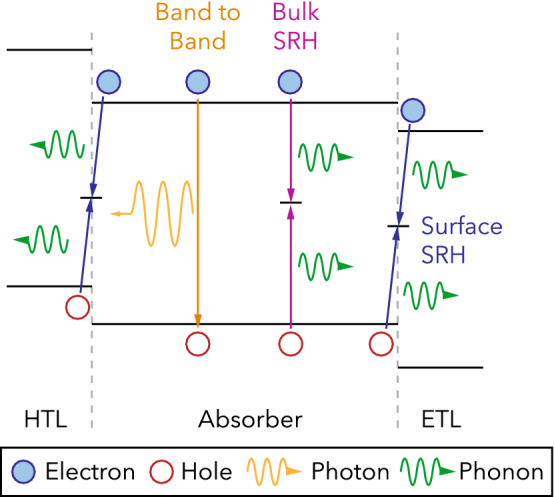
Schematic of different recombination mechanisms in a hole transport layer (HTL)-Absorber-electron transport layer (ETL) device. Figure adapted from Ref [16]

By default, two established models for recombination are included in Driftfusion: band-to-band recombination and trap-mediated Shockley-Read-Hall (SRH) recombination. Figure 5 is a simplified energy level schematic illustrating these mechanisms. The recombination expressions can be modified in the source terms of the Equation Editor (Sect. 4.5).

#### Band-to-band recombination

The rate of band-to-band recombination $r_{\mathrm {btb}}$ (also commonly termed direct, radiative or bimolecular recombination) is proportional to the product of the electron and hole densities at a given location such that

42 $$\begin{aligned} r_{\mathrm {btb}}(x,t) = B(x)(n(x,t)p(x,t) - n_{\mathrm {i}}(x)^2), \end{aligned}$$

where *B* is the band-to-band recombination rate coefficient. The $n_{\mathrm {i}}^2$ term is equivalent to including an expression for thermal generation and ensures that $np \geqslant n_i^2$ at steady-state.

#### Shockley-Read-Hall recombination

Recombination via trap states is modelled using a simplified SRH recombination [39] expression $r_{\mathrm {SRH}}$ for which the capture cross section, mean thermal velocity of carriers, and trap density are collected into SRH time constants, $\tau _{n,\mathrm {SRH}}$ and $\tau _{p,\mathrm {SRH}}$ for electrons and holes, respectively,

43 $$\begin{aligned}&r_{\mathrm {SRH}}(x,t) \nonumber \\&\quad = \dfrac{n(x,t)p(x,t) - n_{\mathrm {i}}(x)^2}{\tau _{n,\mathrm {SRH}}(x)(p(x,t)+p_{\mathrm {t}}(x)) + \tau _{p,\mathrm {SRH}}(x)(n(x,t)+n_{\mathrm {t}}(x))}.\nonumber \\ \end{aligned}$$

Here, $n_{\mathrm {t}}$ and $p_{\mathrm {t}}$ are parameters that define the dependence of the recombination rate on the trap level and are given by the electron and hole densities when their respective QFLs are at the position of the trap energy $E_{\mathrm {t}}$,

44 $$\begin{aligned}&n_{\mathrm {t}}= {} N_{\mathrm {CB}} \left( e^{\left( {\frac{-\varPhi _{\mathrm {EA}}- E_{\mathrm {t}}}{k_\mathrm {B}T}}\right) } + \gamma \right) ^{-1}, \end{aligned}$$

45 $$\begin{aligned}&p_{\mathrm {t}}= {} N_{\mathrm {VB}} \left( e^{\left( {\frac{E_{\mathrm {t}} + \varPhi _{\mathrm {IP}}}{k_\mathrm {B}T}}\right) } + \gamma \right) ^{-1}. \end{aligned}$$

It should be noted that Eq. 43 is valid only when trapped carriers are in thermal equilibrium with those in the bands. It follows that the rate of trapping and de-trapping of carriers is assumed to be fast compared to the timescale being simulated, such that the approximation is reasonable. In the current version of Driftfusion we also assume that the quantity of trapped carriers is negligible compared to that of the free carriers such that trapped carriers can be neglected in Poisson’s equation.

**Figure:**
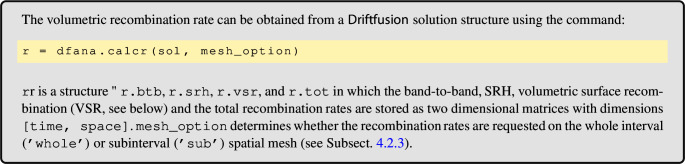

**Figure:**
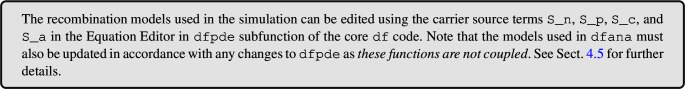

#### Surface recombination at interfaces

Abrupt interface models typically use a SRH surface recombination model to determine the recombination flux, $R_\mathrm {int}$ between majority carriers $n_\mathrm {s}$ and $p_\mathrm {s}$ at the interface between two materials (see Fig. 4a) such that [20]

46 $$\begin{aligned} R_\mathrm {int}(t) = \dfrac{n_\mathrm {s}(t)p_\mathrm {s}(t) - n_{\mathrm {i}}^2}{\frac{1}{s_n} (p_\mathrm {s}(t)+p_{\mathrm {t}}) + \frac{1}{s_p} (n_\mathrm {s}(t)+n_{\mathrm {t}})}. \end{aligned}$$

Here, $s_n$ and $s_p$ are the surface recombination velocities for electrons and holes at the interface. This model implies that the electron and hole populations in the two materials have delocalised wave functions that overlap significantly such that recombination events are probable.

Since Driftfusion uses discrete interfacial regions, in order to obtain an equivalent recombination flux to the abrupt interface model, we convert Eq. 46 into a volumetric surface recombination rate $r_{\mathrm {vsr}}$ by distributing the recombination uniformly across a zone of thickness, $d_\mathrm {vsr}$ within the interface (see Figure S.4), such that $r_{\mathrm {vsr}} = R_\mathrm {int}/d_\mathrm {vsr}$. By default the recombination zone is automatically located next to the interface with the highest minority carrier density at equilibrium. To obtain an expression for $r_{\mathrm {vsr}}$, Eqs. 35 and 36 can be rearranged to express $n_\mathrm {s}$ and $p_\mathrm {s}$ in terms of $n(x_n)$ and $p(x_p)$ to yield

47 $$\begin{aligned}&n_\mathrm {s} = e^{-\alpha x_n} \left( n(x_n) - \dfrac{j_{n,s}}{k_B T \alpha \mu _n}(1-e^{\alpha x_n}) +... \right. \nonumber \\&~~~~~~~~~~~~~~~~~\quad \left. \dfrac{r}{k_B T \alpha ^2 \mu _n}(1 -e^{\alpha x_n} + \alpha x_n) \right) , \end{aligned}$$

48 $$\begin{aligned}&p_\mathrm {s} = e^{-\beta x_p} \left( p(x_p) - \dfrac{j_{p,s}}{k_B T \beta \mu _p}(1- e^{\beta x_p}) +... \right. \nonumber \\&\quad ~~~~~~~~~~~~~~~~~\left. \dfrac{r}{k_B T \beta ^2 \mu _p}(1 -e^{\beta x_p} + \beta x_p) \right) . \end{aligned}$$

For sufficiently high values of $\mu _n$, and $\mu _p$ the $n(x_n)$ and $p(x_p)$ terms dominate Eqs. 47 and 48 and the carrier density profiles within the interfaces tend towards purely exponential functions, such that $n_\mathrm {s} \approx n(x_n) e^{-\alpha x_n}$ and $p_\mathrm {s} \approx p(x_p) e^{-\beta x_p}$. In many instances it is then sufficient to approximate the volumetric surface recombination rate within the recombination zone as

49 $$\begin{aligned}&r_{\mathrm {vsr}}(x,t) \nonumber \\&\quad =\dfrac{n(x,t)e^{-\alpha x_n} p(x,t)e^{-\beta x_p} - n_{\mathrm {i}}^2}{\tau _{n,\mathrm {vsr}}(p(x,t)e^{-\beta x_p}+p_{\mathrm {t}}) + \tau _{p,\mathrm {vsr}}(n(x,t)e^{-\alpha x_n}+n_{\mathrm {t}})},\nonumber \\ \end{aligned}$$

where α and β are given in Eqs. 37 and 38, and the $d_\mathrm {vsr}$ term is subsumed into the volumetric surface recombination time constants, $\tau _{n,\mathrm {vsr}}$ and $\tau _{p,\mathrm {vsr}}$ such that

50 $$\begin{aligned}&\tau _{n,\mathrm {vsr}}= \frac{d_\mathrm {vsr}}{s_n}, \end{aligned}$$

51 $$\begin{aligned}&\tau _{p,\mathrm {vsr}} = \frac{d_\mathrm {vsr}}{s_p}. \end{aligned}$$

We stress here that this is not a physically motivated model in the sense that we do not anticipate recombination to be distributed uniformly throughout a recombination zone in reality. This approach does however result in a good approximation to the established abrupt interface surface recombination model for a wide variety of devices and conditions (see Sect. 5.4).

**Figure:**
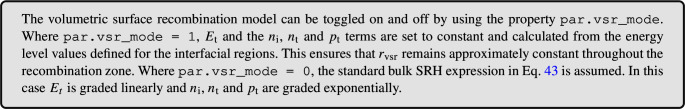

**Figure:**
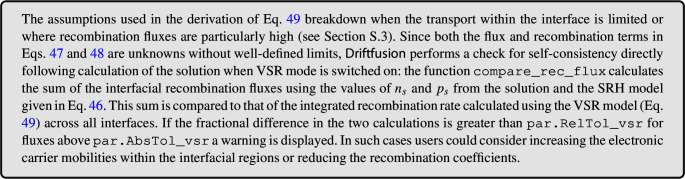

### Initial conditions

At present two sets of initial conditions are used in Driftfusion, dependent on the number of layers. These conditions are designed to be consistent with the boundary conditions and to minimise the error in the space charge density at junctions which can lead to large electric fields and convergence failure when solving for the equilibrium conditions.

#### Single layered devices

A linearly varying electrostatic potential and exponentially varying electronic carrier densities over the layer thickness *d* are used as the initial conditions (Eqs. 53, 54, and 52) when simulating a single layer. Uniform ionic carrier density profiles are used throughout the layer to guarantee ionic defect charge neutrality (Eqs. 56 and 55).

52 $$\begin{aligned} V(x)= & {} \dfrac{x}{d}V_{bi}, \end{aligned}$$

53 $$\begin{aligned} n(x)= & {} n_{0,l} \exp \left( \ln \left( \dfrac{n_{0,r}}{n_{0,l}} \right) \dfrac{x}{d} \right) , \end{aligned}$$

54 $$\begin{aligned} p(x)= & {} p_{0,l} \exp \left( \ln \left( \dfrac{p_{0,r}}{p_{0,l}} \right) \dfrac{x}{d} \right) , \end{aligned}$$

55 $$\begin{aligned} c(x)= & {} N_\mathrm {cat}(x), \end{aligned}$$

56 $$\begin{aligned} a(x)= & {} N_\mathrm {ani}(x). \end{aligned}$$

Here, the built-in potential $V_{\mathrm {bi}}$ of the device is determined by the difference in boundary electrode workfunctions $\varPhi _{l}$ and $\varPhi _{r}$,

57 $$\begin{aligned} qV_{\mathrm {bi}} = \varPhi _{r} - \varPhi _{l} . \end{aligned}$$

#### Multilayered devices

For multilayered devices the electrostatic potential is set to fall uniformly throughout the device (Eq. 58), while the electronic carrier densities are chosen to be the equilibrium densities for the individual layers ($n_0$ and $p_0$). As with the single layers, the ionic carriers are given a uniform density (Eqs. 59–61), guaranteeing local electro-neutrality.

58 $$\begin{aligned} V(x)= & {} \dfrac{x}{d_\mathrm {dev}}V_{bi}, \end{aligned}$$

59 $$\begin{aligned} n(x)= & {} n_0(x), \end{aligned}$$

60 $$\begin{aligned} p(x)= & {} p_0(x), \end{aligned}$$

61 $$\begin{aligned} c(x)= & {} N_\mathrm {cat}(x), \end{aligned}$$

62 $$\begin{aligned} a(x)= & {} N_\mathrm {ani}(x). \end{aligned}$$

Here, the device thickness $d_\mathrm {dev}$ is the sum of the individual layer thicknesses $d_i$ ($d_\mathrm {dev} = \sum _{i} d_i$). Driftfusion auto-detects the number of layers in the device and uses the appropriate set of initial conditions when running the equilibrate protocol to obtain the equilibrium solutions for the device (Sect. 4.3).

**Figure:**
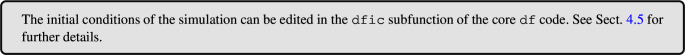

### Boundary conditions

Solving Eq. 17 and Eqs. 31–34 requires two constants of integration for each variable, which are provided by the system boundary conditions. For the charge carriers, Neumann (defined-flux value) conditions are used to set the flux density into and out of the system. The electrostatic potential uses Dirichlet conditions (defined-variable value) such that the potential is fixed at both boundaries at each point in time as detailed in Sect. 3.9.1. The details of these boundary conditions are discussed in the following subsections.

#### Electrostatic potential boundary conditions

In Driftfusion the electrostatic potential at the left-hand boundary is set to zero (Eq. 63) and used as the reference potential. The applied electrical bias $V_{\mathrm {app}}$ minus the potential drop across the external series resistance $V_{Rs}$ is applied to the right-hand boundary as described in Eq. 64.

63 $$\begin{aligned}&V_l(t)=0, \end{aligned}$$

64 $$\begin{aligned}&V_r(t)=V_{\mathrm {bi}}-V_{\mathrm {app}}(t)+V_{\mathrm {Rs}}(t). \end{aligned}$$

Here, Ohm’s law is used to calculate $V_{Rs}$ from the electron and hole flux densities,

65 $$\begin{aligned} V_{Rs}(t)= q(j_{p,r}(t) - j_{n,r}(t))R_s, \end{aligned}$$

where $R_s$ is the area-normalised series resistance, given by the product of the external series resistance and the device active area. Setting $R_s$ to a relatively high value (e.g. $R_s = 10^6$ ${\Omega }$ $\hbox {cm}^2$) approximates an open circuit condition for devices with metal electrodes. Technically this can be achieved using the lighton_Rs protocol (see Sect. 4.4 for a description of protocols).^7^

**Figure:**
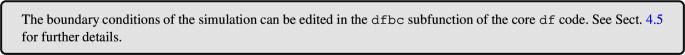

**Figure:**
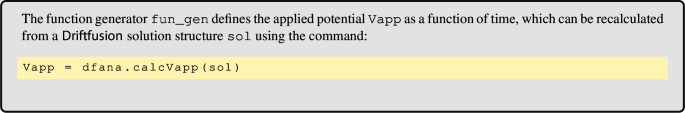

#### Carrier selectivity and surface recombination at the system boundaries

Many architectures of semiconductor device, including solar cells and LEDs employ selective contact layers that block minority carriers from being extracted (or injected) via energetic barriers. These are known variously as transport layers, blocking layers, blocking contacts, or selective contacts. For solar cells semiconductor layers are typically sandwiched between two metallic electrodes constituted of metals or highly-doped semiconductors. Such materials can be numerically challenging to simulate owing to their high charge carrier densities and thin depletion widths. Consequently, a common approach is to use boundary conditions defining charge carrier extraction and recombination flux densities to simulate the properties of either the contact or electrode material. It should be noted, however, that the employment of fixed electrostatic potential boundary conditions (as defined in Sect. 3.9.1) implies that the potential falls *within the discrete system* and not within the electrodes. This approximation is only realistic for contact materials with vanishingly small depletion widths (infinite interfacial capacitances) i.e. metals and highly doped semiconductors. It follows that to accurately simulate semiconductor contacts layers with finite depletion regions these layers must also be included within the discretised system.

For electronic carriers the surface recombination velocity coefficients $s_n$ and $s_p$ determine the carrier extraction/recombination rate at the boundaries of the system. For majority carriers in solar cells, high values of $s_n$ and $s_p$ (e.g. $>10^7$ cm $\hbox {s}^{-1}$ [40]) are advantageous for carrier extraction, while low values imply poor contact extraction properties. For minority carriers, high values of $s_n$ and $s_p$ are typically undesirable as they imply high rates of surface recombination at the electrode. In Driftfusion the expressions for electronic boundary carrier flux densities, $j_n$ and $j_p$ are given by the typical first-order equations

66 $$\begin{aligned} j_{n,l} (t)= & {} s_{n,l}(n_l(t) - n_{0, l}), \end{aligned}$$

67 $$\begin{aligned} j_{p,l} (t)= & {} s_{p,l}(p_l(t) - p_{0, l}), \end{aligned}$$

68 $$\begin{aligned} j_{n,r} (t)= & {} s_{n,r}(n_r(t) - n_{0, r}), \end{aligned}$$

69 $$\begin{aligned} j_{p,r} (t)= & {} s_{p,r}(p_r(t) - p_{0, r}), \end{aligned}$$

where $n_{0, l}$, $n_{0, r}$, $p_{0, l}$, and $p_{0, r}$ are the equilibrium carrier densities at the left (x=0) and right-hand ($x=d_\mathrm {dev}$) boundaries, calculated using Eqs. 8 and 9 under the assumption that the semiconductor QFLs are at the same energy as the electrode Fermi energy, which is further assumed to remain constant. Hence, for the left-hand boundary $n_{0, l}$ and $p_{0, l}$ are given by Eqs. 70 and 71:

70 $$\begin{aligned} n_{0, l}= & {} N_{\mathrm {CB}} \left( e^{\frac{\varPhi _l - \varPhi _\mathrm {EA}}{k_\mathrm {B}T}} + \gamma \right) ^{-1}, \end{aligned}$$

71 $$\begin{aligned} p_{0, l}= & {} N_{\mathrm {VB}} \left( e^{\frac{\varPhi _\mathrm {IP} - \varPhi _l}{k_\mathrm {B}T}} + \gamma \right) ^{-1}, \end{aligned}$$

where $\varPhi _{l}$ is the left-hand electrode work function. Analogous expressions are used for $n_{0,r}$ and $p_{0,r}$ at the right-hand boundary. Extraction barriers can also be modelled with this approach by including a term for the barrier energy in the exponent of Eqs. 70 and 71. At present, however, quantum mechanical tunnelling and image charge density models for energetic barriers at the system boundaries are not accounted for in Driftfusion.

#### Ionic carrier boundary conditions

In the simplest case, ionic carriers are confined to the device and do not react at the electrode boundaries. This leads to a set of zero flux density boundary conditions for mobile anions and cations,

72 $$\begin{aligned}&j_{c,l}(t) = 0 , \end{aligned}$$

73 $$\begin{aligned}&j_{a,l}(t) = 0, \end{aligned}$$

74 $$\begin{aligned}&j_{c,r}(t) = 0, \end{aligned}$$

75 $$\begin{aligned}&j_{a,r}(t) = 0. \end{aligned}$$

Where an infinite reservoir of ions exists at a system boundary (such as an electrolyte), a Dirichlet boundary condition defining a constant ion density could alternatively be imposed.

This concludes our description of the physical models employed in Driftfusion. In the following section the system architecture and key commands are introduced as well as a guide on how to get started with using Driftfusion.

## System architecture and how to use Driftfusion

**Fig. 6:**
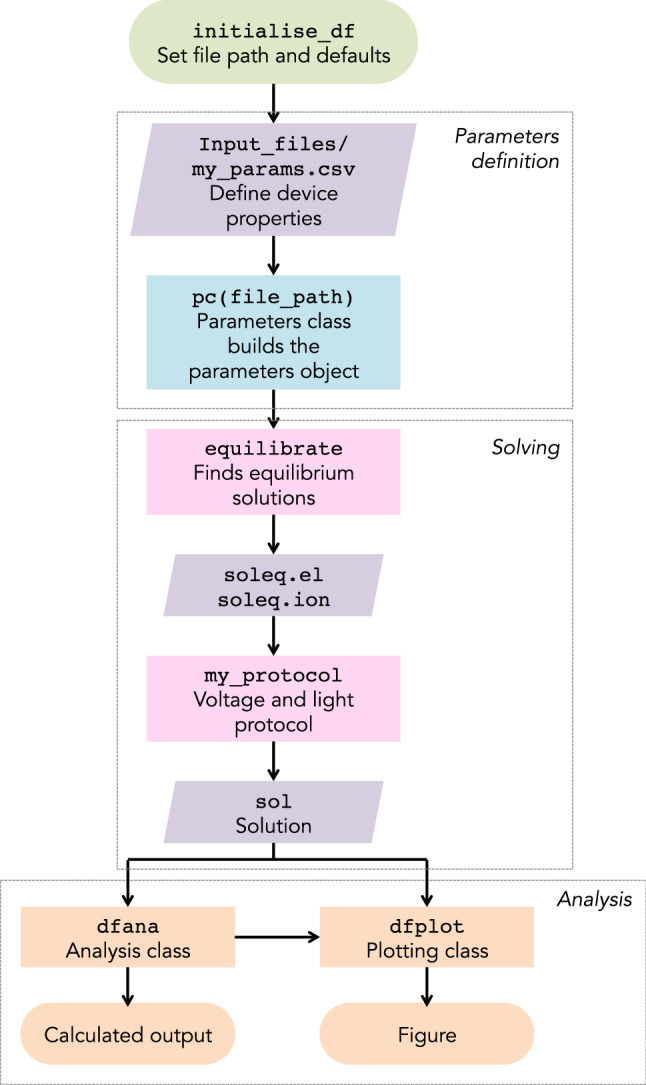
Flow diagram showing the key steps to obtain a solution. A key to the box shapes is given in Fig. 7

Driftfusion is designed such that the user performs a linear sequence of simple procedures to obtain a solution. The key steps are summarised in Fig. 6; following initialisation of the system, the user defines a device by creating a parameters object containing all the individual layer and device-wide properties; the equilibrium solutions (soleq.el and soleq.ion) are then obtained for the device before applying a voltage and light protocol, which may involve intermediate solutions; Once a desired solution (sol) has been obtained, analysis and plotting functions can be called to calculate outputs and visualise the solutions. Below, the principal functions are discussed in further detail.

### Initialising the system: initialise_df

At the start of each MATLAB session, initialise_df needs to be called from within the Driftfusion parent folder (*not one of its subfolders*) to add the program folders to the file path and set plotting defaults. This action must be completed before any saved data objects are loaded into the MATLAB workspace to ensure that objects are associated with their corresponding class definitions.

**Fig. 7:**
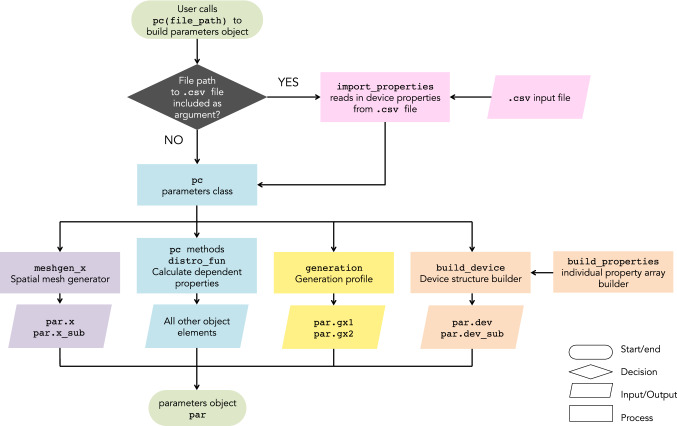
Flow diagram showing the key processes involved in building a parameters object

### Defining device properties and creating a parameters object: pc(file_path)

The parameters class, pc contains the default device properties and functions required to build a parameters object, which we shall denote herein as par. The parameters object defines both layer-specific and device-wide properties. Layer-specific properties must be a cell or numerical array containing the same number of elements as there are layers, *including* interface layers. For example, a three-layer device with two heterojunctions requires layer-specific property arrays to have five elements. Examples can be found in the Input_files folder (see Sect. 4.2.1).

Figure 7 shows the processes through which the main components of the parameters object are built; The user is required to define a set of material properties for each layer. The comments in pc describe each of the parameters in detail and give their units (also see Supplemental Information, Table S.3 for quick reference); Several subfunctions (methods) of pc then calculate dependent properties, such as the equilibrium carrier densities for example, from the choice of probability distribution function (prob_distro_function) and other user-defined properties. The treatment of properties in the device interfaces is dealt with, and can be changed, using the device builder build_device (see below).

#### Importing properties

Typically, the most important user-definable properties (Table 1) are stored in a .csv file, which is easily editable with a spreadsheet editor such as LibreOffice. The file path to the .csv file can then be used as an input argument for pc, for example:

**Figure:**
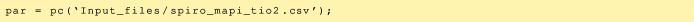

This functionality allows the user to easily create and store sets of key device parameters without editing pc. Default values for properties set in the parameters class pc will be overwritten by the values in the .csv file during creation of the parameters object par. New properties defined in pc can easily be added to the .csv file provided that they are also included in import_properties, which tests to see which properties are present in the text file and reads them into the parameters object where present. Following the properties read-in step performed by import_properties, the number of rows in the layer_type column (see Sect. 4.2.2) is used for error checking all other entries to confirm that properties have been defined for each discrete layer of the system (this does not include the electrode rows, which are pseudo-layers). To avoid potential incompatibility, any new user-defined material properties defined in pc, which have distinct values for each layer, should be included in the .csv file and added to the list of importable properties in import_properties, as well as build_device. An example of how to do this is given in the Supplemental Information, Sect. S.6.

#### Layer types

**Fig. 8:**
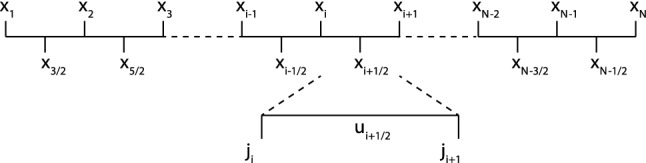
The computational spatial grid. Variables are solved for on the subintervals while flux densities are calculated on the integer intervals. Figure concept taken from Ref [41]

**Table 1:** Key to properties contained in external .csv parameters files and their constraints

Column heading	Description	Options/range	Units	Section ref.
layer_type	Layer type	electrode, layer, active, interface	–	4.2.2
material	Chemical short-form name	Materials contained within ./Libraries/Index_of_Refraction_library.xls	–	4.2.7
thickness	Layer thickness	>0	cm	4.2.3
layer_points	Number of points in the layer	⩾3	–	4.2.3
xmesh_coeff	A parameter defining how densely the spatialmesh points are concentrated at theboundaries of the layer for xmesh_type = ‘erf-linear’	>0	–	4.2.3
Phi_EA	Electron affinity^1^	–	eV	3.1
Phi_IP	Ionisation potential^1^	–	eV	3.1
EF0	Equilibrium Fermi energy^2,3^	⩾Phi_IP, ⩽Phi_EA	eV	3.2.1
Et	SRH trap energy level (single trap level model)	⩾Phi_IP, ⩽Phi_EA	eV	3.7, 3.7.3
Nc	Effective density of conduction band states	>0	$\hbox {cm}^{-3}$	3.2.1
Nv	Effective density of valence band states	>0	$\hbox {cm}^{-3}$	3.2.1
Ncat	Intrinsic Schottky defect density(mobile cations) at equilibrium	<c_max	$\hbox {cm}^{-3}$	3.3
Nani	Intrinsic Schottky defect density (mobile anions) at equilibrium	<a_max	$\hbox {cm}^{-3}$	3.3
c_max	Limiting mobile cation density	>Ncat	$\hbox {cm}^{-3}$	3.4.2
a_max	Limiting mobile anion density	>Nani	$\hbox {cm}^{-3}$	3.4.2
mu_n	Electron mobility	⩾0	$\hbox {cm}^2$ $\hbox {V}^{-1}$ $\hbox {s}^{-1}$	3.4
mu_p	Hole mobility	⩾0	$\hbox {cm}^2$ $\hbox {V}^{-1}$ $\hbox {s}^{-1}$	3.4
mu_c	Cation mobility	⩾0	$\hbox {cm}^2$ $\hbox {V}^{-1}$ $\hbox {s}^{-1}$	3.4
mu_a	Anion mobility	⩾0	$\hbox {cm}^2$ $\hbox {V}^{-1}$ $\hbox {s}^{-1}$	3.4
epp	Relative dielectric constant	>0	–	3.3
g0	Uniform generation rate	⩾0	$\hbox {cm}^{-3}$ $\hbox {s}^{-1}$	3.6, 4.2.7
B	Band-to-band recombination rate coefficient	>0	cm $\hbox {s}^{-1}$	3.7.1
taun	SRH electron lifetime	>0	s	3.7.2
taup	SRH hole lifetime	>0	s	3.7.2
sn	Electron surface recombination $\hbox {velocity}^{2,4}$	>0	cm $\hbox {s}^{-1}$	3.7.3, 3.9
sp	Hole surface recombination $\hbox {velocity}^{2,4}$	>0	cm $\hbox {s}^{-1}$	3.7.3, 3.9
vsr_zone_loc	Volumetric interfacial surface recombination zone location	auto, L, C, R	–	3.7.3
Red	Layer colour red RGB triplet component	0-1	norm.	–
Green	Layer colour green RGB triplet component	0-1	norm.	–
Blue	Layer colour blue RGB triplet component	0-1	norm.	–
optical_model	Optical model	uniform, Beer–Lambert	–	3.6,4.2.7,3.6.1
xmesh_type	Spatial mesh type	linear, erf-linear	–	4.2.3
side	Illumination side	left, right	–	–
N_ionic_species	Number of mobile ionic species	0, 1 (cations), 2 (cations and anions)	–	–

$^1\hbox {Note}$ that contrary to convention, Phi_EA and Phi_IP take negative values for consistency with other energies referenced to the electron energy scale. ^2^These properties are required for electrode pseudo-layers, but all other properties are ignored in these rows. $^3\hbox {For}$ electrode layers, entries for EF0 are stored in the parameters object (par) as the distinct properties Phi_left and Phi_right rather than as part of the EF0 array. Phi_left and Phi_right take negative values for consistency with other energies referenced to the electron energy scale. ^4^ For electrode layers, entries for sn and sp are stored in the parameters object (par) as the distinct properties sn_l, sn_r, sp_l, and sp_r rather than as part of the sn and sp arrays

Layer types, set using the layer_type property, flag how each layer should be treated. Driftfusion currently uses four layer types:

1. ‘electrode’: A pseudo-layer which defines the boundary properties of the system. These are not discrete layers and do not appear in visualised outputs.
2. ‘layer’: A slab of semiconductor for which all properties are spatially constant.
3. ‘active’: As layer but flags the active layer of the device. The number of the first layer designated ‘active’ is stored in the active_layer property and is used for calculating further properties such as the active layer thickness, d_active. Flagging the active layer proves particularly useful when automating explorations.
4. ‘interface’: An interfacial region between two different material layers. The properties of the interface are varied according to the specific choice of grading method as defined in build_device (see below). *It is critical that interfacial layers are included between material layers with different energy levels and eDOS values i.e. at heterojunctions. See the included default input files for examples of how to set up devices with heterojunctions.*

#### Spatial mesh

The computational grid is divided into *N* intervals with N-1 subintervals, where the position of the subintervals is defined by $x_{i +1/2} = (x_{i+1} + x_{i})/2$ for i=1,2,3,...,N-1. pdepe solves for the variable values $u_{i +1/2}$ on the subintervals ($x_{i+1/2}$) and their associated flux densities $j_{i}$ on the integer intervals ($x_{i}$) as illustrated in Fig. 8.

Owing to the use of a finite element discretisation scheme the details of the spatial mesh in Driftfusion are of critical importance to ensure fast and reliable convergence. In this release of Driftfusion two types of spatial mesh are available:

1. ‘linear’: Linear piece-wise spacing
2. ‘erf-linear’: Mixed error function (bulk regions)- linear (interfacial regions) piece-wise spacing

meshgen_x generates integer and subinterval spatial meshes, x and x_sub, respectively (see Fig. 8), based on the layer thickness, the number of layer_points defined in the device properties, and the xmesh_type. The solution is interpolated for the integer grid points when generating the output solution matrix sol.u (see Sect. 4.6). The property xmesh_coeff controls the spread of points for regions where an error function is used for point spacing i.e. layer and active layer types: higher values result in higher point densities close to the layer boundaries. In general we recommend using ‘erf-linear’ for devices with relatively high ionic defect densities as, where ionic carriers are confined, high point densities are required at the layer boundaries to resolve the carrier distributions. Where the depletion of ionic carriers extends into the bulk, xmesh_ceoff can be reduced to increase the bulk point density.

#### Time mesh

pdepe uses an adaptive time step for forward time integration and solution output is interpolated for the user-defined time mesh. Convergence of the solver is weakly dependent of the user-defined time mesh interval spacing and strongly dependent on the maximum time and the maximum allowable time step. These can be adjusted by changing the tmax and MaxStepFactor properties of the parameters object (see Sect. 4.2). The options for different time mesh types (tmesh_type) are:

1. ‘linear’ or 1
2. ‘log 10’ or 2

Typically, the time mesh is changed frequently for intermediate solutions within protocols to accommodate the different timescales on which carriers move. For example, in some cases the ionic carriers may be frozen to obtain a stable short timescale solution for the electronic carriers, before a second, longer timescale, solution is calculated with the ionic carriers mobile. Similarly the tmesh_type is adjusted dependent on the voltage and light conditions. For example during a J-V scan a linear time mesh is used in keeping with the linear change in the applied voltage with time. In other instances a logarithmic mesh is more appropriate in order to resolve time periods over which the carrier time derivatives are larger.

#### The device structures

build_device and build_property are called during creation of the parameters object to build two important data structures, which we call the ‘device structures’: dev, defined on the integer grid intervals ($x_i$), is used to determine the initial conditions only, while dev_sub, defined on the subintervals ($x_{i+\frac{1}{2}}$), is used by the pdepe solver function. dev and dev_sub contain arrays defining all spatially varying properties at every location within the device including the interfacial regions. build_property enables the user to specify different types of interface grading for each property listed in build_device. At present there are four generic grading option types:

1. ‘zeroed’: The value of the property is set to zero throughout the interfacial region.
2. ‘constant’: The value of the property is set to be constant throughout the interfacial region. Note that the user must define this value in the .csv file.
3. ‘lin_graded’: The property is linearly graded using the property values of the adjoining layers.
4. ‘exp_graded’: The property is exponentially graded using the property values of the adjoining layers.

For the volumetric surface recombination scheme, described in Sect. 3.7.3, we also introduce parameter-specific grading schemes for the VSR time constants:

1. ‘taun_vsr’: Sets $\tau _{n,\mathrm {vsr}}$ according to Eq. 50.
2. ‘taup_vsr’: Sets $\tau _{p,\mathrm {vsr}}$ according to Eq. 51.

The interface grading type for each property is set in the build_device function e.g. the default grading type for the electron affinity is ‘lin_graded’.

Dependent on the choice of grading option, input values may, or may not, be required for a given material property in the interfacial layers. For example, when using the ‘lin_graded’ option a value is not needed because the interface property values are calculated from those of the adjacent layers. By contrast, when using the ‘constant’ option, a property value does need to be specified for the interfacial layer to avoid an error. Since property values that are not required are ignored, *we recommend that users specify all property values for all layers* to future-proof against problems arising when experimenting with grading options.

#### The electronic carrier probability distribution function

The class distro_fun defines the electronic carrier probability distribution function for the model and is called to calculate the equilibrium boundary, and initial carrier densities when building the parameters object. At present there are two available options for the choice of probability distribution function:

1. ‘Boltz’: The Boltzmann approximation (γ=0)
2. ‘Blakemore’: The Blakemore approximation (γ>0 see Sect. 3.2.1)

While the Boltzmann approximation results in marginally faster calculations owing to the absence of a diffusion enhancement, we recommend using Blakemore statistics for their extended domain of validity.

#### The electronic carrier generation profile function

The function generation calculates the two generation profiles gx1 and gx2, which can be used for a constant bias light and a pulse source for example, at each spatial location in the device for the chosen optical_model and light sources (see Sect. 3.6 and the flow diagram in the Supplemental Information Figure S.5). The light sources can be set using the light_source1 and light_source2 properties. There are two options for the optical_model:

1. ‘uniform’: a uniform volumetric generation rate defined by the property g0 is applied to bulk layers with layer and active layer types. The multiplier properties int1 and int2 define the intensities for light sources 1 and 2 respectively.
2. ‘Beer_Lambert’: The generation profile follows the Beer–Lambert law as detailed in Sect. 3.6.1 with material layer optical properties taken from ./Libraries/Index_of_Refraction_library.xls for the corresponding materials defined in the material cell array. As with uniform generation, the generation rate profile is multiplied by the intensity properties int1 and int2 before being applied.

An arbitrary generation profile calculated using an external program (such as Solcore [42] or the McGeHee Group’s Transfer Matrix code [43]) can be inserted into the parameters object par by overwriting the generation profile properties gx1 or gx2 following creation of the object. The profile must be interpolated for the subinterval ($x_{i+1/2}$) grid points defined by x_sub (see Sect. 4.2.3). We also recommend that the generation rate is set to zero within the interfacial regions to avoid stability issues.

The light source time-dependencies are controlled using the g1_fun_type and g2_fun_type properties, which define the function type (e.g. sine wave) and the g1_fun_arg and g2_fun_arg properties, which are coefficient arrays for the function generator (see Sect. 4.5.5) e.g. the frequency, amplitude, etc.

### Protocols: equilibrate

Once a device has been created and stored in the MATLAB workspace as a parameters object the next step is to find the equilibrium solution for the device. The function equilibrate starts with the initial conditions described in Sect. 3.8 and runs through a number of steps to find the equilibrium solutions, with and without mobile ionic carriers, for the device described by par. The output structure soleq contains two solutions (see Sect. 4.6);

1. soleq.el: only the electronic charge carriers are mobile and are at equilibrium.
2. soleq.ion: electronic and ionic carriers are mobile and are at equilibrium.

Storing both solutions in this way allows devices with and without mobile ionic charge to be compared easily.

**Table 2:** List of protocols available in Driftfusion at the time of publication

Command	Description
changeLight	Switches light intensity using incremental intensity steps.
doCV	Cyclic Voltammogram (CV) simulation using triangle wave function generator .
doIMPS	Intensity Modulated Photocurrent Spectroscopy (IMPS) simulation at a specified frequency and light intensities.
doIMVS	Switches to open circuit (OC), runs to steady-state OC, then performs Intensity Modulated Photovoltage Spectroscopy (IMVS) measurement simulation at a specified frequency and light intensities.
doJV	Forward and reverse current–voltage (JV) scan with options for dark and constant illumination conditions.
doLightPulse	Uses light source 2 with square wave generator superimposed on light source 1 (determined by the initial conditions) to optically pulse the device.
doSDP	Step, Dwell, Probe (SDP) measurement protocol: Jump to an applied potential, remain at the applied potential for a specified dwell time and then perform optically pulsed current transient. See Ref. [44] for further details of the experimental protocol.
doSPV	Surface PhotoVoltage (SPV) simulation: Switches bias light on with high series resistance.
doTPV	Transient PhotoVoltage (TPV) simulation: Switches to open circuit with bias light and optically pulses the device.
equilibrate	Start from base initial conditions and find equilibrium solutions without mobile ionic charge (soleq.el) and with mobile ionic charge (soleq.ion).
findVoc	Obtains steady-state open circuit condition using Newton–Raphson minimisation.
findVocDirect	Obtains an approximate steady-state open circuit condition using 1 $\hbox {M}\Omega$ $\hbox {cm}^2$ series resistance.
genIntStructs	Generates solutions at various light intensities.
genIntStructsRealVoc	Generates open circuit solutions at various light intensities.
genVappStructs	Generates solutions at various applied voltages.
jumptoV	Jumps to a new applied voltage and stabilises the cell at the new voltage for a user-defined time period.
lightonRs	Switches on the light for a specified period of time with a user-defined series resistance.
stabilize	Runs a set of initial conditions to a steady-state.
sweepLight	Linear sweep of the light intensity over a user-defined time period.
transient_nid	Simulates a transient ideality factor ($n_{id}$) measurement protocol [16]
VappFunction	Applies a user-defined voltage function to a set of initial conditions.

### Protocols: General

**Fig. 9:**
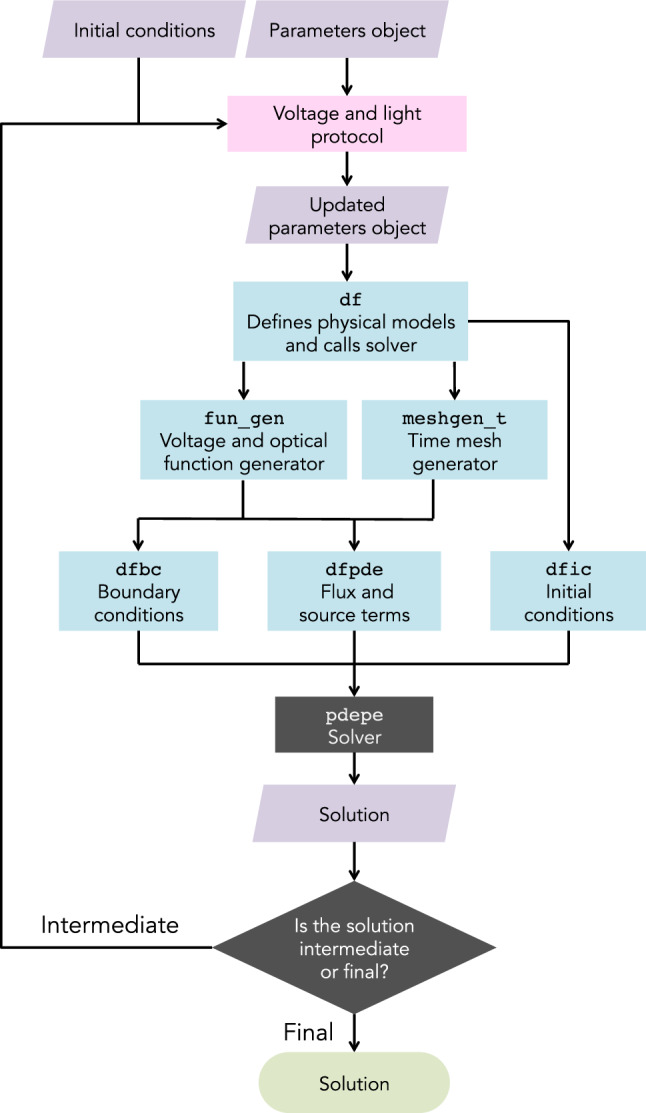
Flow diagram illustrating execution of a Driftfusion protocol

A Driftfusion protocol is defined as a function that contains a series of instructions that takes an input solution (initial conditions) and produces an output solution. For many of the existing protocols (listed in Table 2) with the exception of equilibrate, the input is one of the equilibrium solutions, soleq.el or soleq.ion. Figure 9 is a flow diagram illustrating the key functions called during execution of a protocol.

Protocols typically start by creating a temporary parameters object that is a duplicate of the input solution parameters object. This temporary object can then be used to write new voltage and light parameters that will be used by the function generator to define the generation rate at each point in space and time and the potential at the boundary at each point in time. Additional parameters, for example those defining the output time mesh or carrier transport are also frequently adjusted. The approach is to split a complex experimental protocol into a series of intermediate steps that facilitate convergence of the solver. For example, where ionic mobilities are separated by many orders of magnitude from electronic mobilities, a steady-state solution is easiest found by temporarily increasing the ionic mobilities to be similar to that of the electronic carriers using the K_a and K_c properties. The simulation can then be run with an appropriate time step and checked to confirm that a steady-state has been reached. The possibilities are too numerous to list here and users are encouraged to investigate the existing protocols listed in Table 2 in preparation of writing their own.

### The Driftfusion master function df

df is the core of Driftfusion. The function takes the device parameters, and voltage and light conditions, calls the solver and outputs the solution (Fig. 9). df contains three important subfunctions: dfpde, dfic, and dfbc, which respectively define the continuity equations to be solved, deal with the initial conditions, and define the boundary conditions of the system.

#### Driftfusion Partial Differential Equation function dfpde and the Equation Editor

dfpde defines the equations to be solved by MATLAB’s Partial Differential Equation: Parabolic and Elliptic solver toolbox (pdepe) [31]. For one-dimensional Cartesian co-ordinates, pdepe solves equations of the form

76 $$\begin{aligned} C \left( x,t,u,\dfrac{\partial u}{\partial x} \right) \dfrac{\partial u}{\partial t} = \dfrac{\partial }{\partial x} \left( F \left( x,t,u,\dfrac{\partial u}{\partial x} \right) \right) + S\left( x,t,u,\dfrac{\partial u}{\partial x} \right) \nonumber \\ \end{aligned}$$

Here, *u* is a vector containing the variables *V*, *n*, *p*, *c*, and *a* at each position in space *x* and time *t*. *C* is a vector defining the prefactor for the time derivative, *F* is a vector determining the flux density terms (note: *F* does *not* denote the electric field in this section), and *S* is a vector containing the source/sink terms for the components of *u*. By default C=1 for charge carriers but could, for example, be used to change the active volume fraction of a layer in a mesoporous structure. The equations can be easily reviewed and edited in the dfpde Equation Editor as shown in Listing 1. Here, device properties that have a spatial dependence are indexed with the variable *i* to obtain the corresponding value at the location given by x_sub(i). A step-by-step example of how to change the physical model using the Equation Editor is given in the Supplemental Information, Section S.6.

**Figure:**
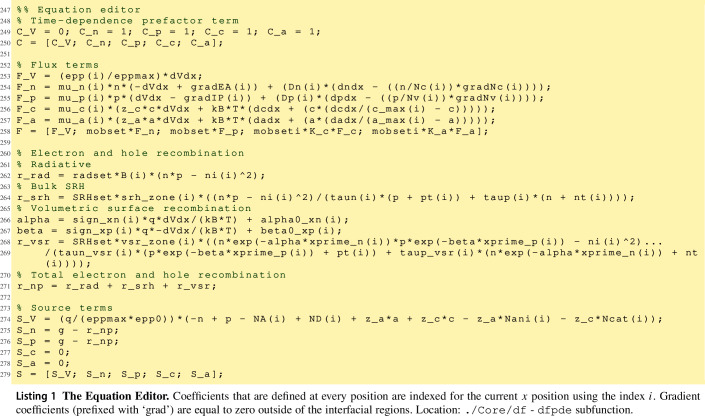

#### Driftfusion Initial Conditions dfic

dfic defines the initial conditions to be used by pedpe. If running equilibrate to obtain the equilibrium solution or running df with an empty input solution, the first set of initial conditions is as described in Sect. 3.8. Otherwise, the final time point of the input solution is used.

#### Driftfusion Boundary Conditions dfbc

**Figure:**
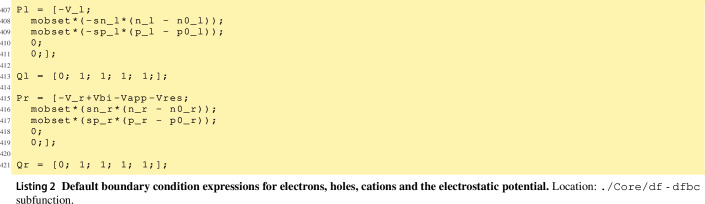

dfbc defines the system boundary conditions. The boundary condition expressions are passed to pdepe using two coefficients *P* and *Q*, with $N_u$ elements, where $N_u$ is the number of independent variables being solved for. The boundary conditions are expressed in the form

77 $$\begin{aligned} P(x,t,u) + Q(x,t)F \left( x,t,u,\dfrac{\partial u}{\partial x} \right) = 0. \end{aligned}$$

For Dirichlet conditions *P* must be nonzero to define the variable values, whereas for Neumann conditions *Q* must be nonzero to define the variable flux. Listing 2 shows how the default Driftfusion boundary conditions (described in Sect. 3.9) are implemented in this release of the code.

In addition to these subfunctions, df also calls two important external functions: the time mesh generator, meshgen_t and the function generator, fun_gen.

#### The time mesh generator meshgen_t

df calls the time mesh generator meshgen_t at the start of the code. As discussed in Sect. 4.2.4, the solver uses an adaptive time step and interpolates the solution to the user-defined mesh. The values of the mesh should be chosen such as to resolve the solution properly on the appropriate timescales. It should be noted that the total time step of the solution tmax and the maximum time step (controlled using the MaxStepFactor property) influence convergence strongly. For this reason, where convergence is proving problematic, it is recommended that either tmax or MaxStepFactor is reduced and the solution obtained in multiple stages.

#### The function generator fun_gen

df calls fun_gen to generate time-dependent algebraic functions that define the applied voltage and light intensity conditions. df includes the ability to call two different light intensity functions with different light sources, enabling users to simulate a constant bias light and additional pump pulse using the square wave generator, for example. Each function type requires a coefficients array with a number of elements determined by the function type and detailed in the comments of fun_gen. Listing 3 is an example from the ./Scripts/VappFunction_script script showing how to define a sine wave function for the applied voltage.

**Figure:**
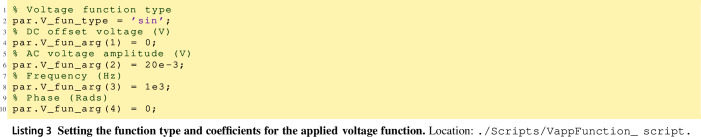

### Solution structures

df outputs a solution structure sol with the following components:

- The solution matrix u: a three dimensional matrix for which the dimensions are [time, space, variable]. The order of the variables is:
  1. Electrostatic potential
  2. Electron density
  3. Hole density
  4. Cation density (where 1 mobile ionic carrier is stipulated)
  5. Anion density (where 2 mobile ionic carriers are stipulated)
- The spatial mesh x.
- The time mesh t.
- The parameters object par.

All other outputs can be calculated from the above by calling methods from dfana.

### Calculating outputs dfana

dfana is a collection of functions (methods) that enable the user to calculate outputs such as carrier currents, quasi-Fermi levels, recombination rates, etc. from the solution matrix u, the parameters object par, and the specified physical models. The use of a class in this instance enables the package syntax dfana.my_calculation(sol) to be used. For example the command

**Figure:**
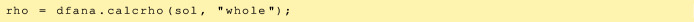

outputs a two dimensional matrix containing the space charge density as function of time and position. Additional examples of how dfana methods can be used to calculate outputs are given in the highlighted boxes of Sect. 3. The full list of the available analysis methods can be viewed and easily navigated by selecting the dfana in the Current Folder window and opening the functions browser sub-window in MATLAB.

**Figure:**
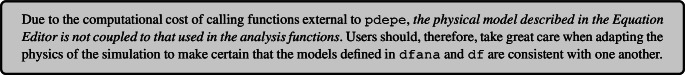

### Plotting outputs dfplot

dfplot is a class containing a collection of plotting methods. Similar to dfana, this enables the package syntax dfplot.my_plot(sol) to be used. For variables plotted as a function of position, an optional vector argument $[t_1, t_2, t_3,... t_m]$ can be included to plot the solution at $t = t_1, t_2, t_3,... t_m$, where *m* is the *m* *th* time point to be plotted. For example the command

**Figure:**
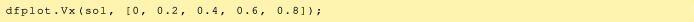

plots the electrostatic potential component of the solution as a function of time at the position t=0, 0.2, 0.4, 0.6, and 0.8 s (an example is shown in Fig. 15a). If no second argument is given then only the final time point is plotted. dfplot also includes the generic property plotting function dfplot.x2d to allow users to easily create new two-dimensional plots.

For variables plotted as a function of time the second argument defines the position. For example the command

**Figure:**
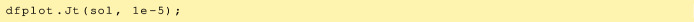

plots the current density for each carrier as a function of position at $x = 10^{-5}$ cm. For plots where variables are integrated over a region of space, the second argument is a vector containing the limits $[x_1 , x_2]$. Further details can be found in the comments of dfplot.

### Getting started and the example scripts

While the underlying system may appear complex, Driftfusion has been designed such that with a few simple commands, users can simulate complex devices and transient optoelectronic experiment protocols. Table 2 is a complete list of protocols available at the time of writing. In addition to the brief guide below, a quick start with up-to-date instructions can be found in the README.md file contained within the Driftfusion GitHub repository, [26] and a series of example scripts for running specific protocols are also presented in the Scripts folder. *New users are advised to study these scripts and adapt them to their own purposes.* In addition, we have written an introductory workshop to guide students through the process of building basic semiconductor devices and applying optical and voltage biases to them. This can be found in the Semiconductor-device-physics-workshop branch of the Driftfusion GitHub repository.

#### How to build a device object, find the equilibrium solution, and run a cyclic voltammogram

In this section some commonly used commands are put together to show new users how to create a device object, obtain the device equilibrium solutions, and run a protocol, which in this example simulates a cyclic voltammogram (CV). The doCV protocol applies a triangular wave voltage function to the device, with optional constant illumination, for a set number of cycles enabling the device current–voltage characteristics at a given scan rate to be calculated.

At the start of each session, the system must be initialised by typing the command

**Figure:**
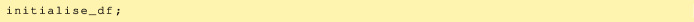

To create a parameters object using the default material and device properties for a Spiro-OMeTAD/perovskite/$\hbox {TiO}_2$ perovskite solar cell the parameters class pc is called with the file path to the relevant .csv file as the input argument:

**Figure:**
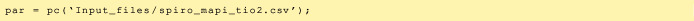

The equilibrium solutions with and without mobile ionic carriers for the device can now be obtained by calling the equilibrate protocol:

**Figure:**
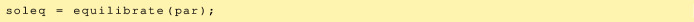

As discussed in Sect. 4.3, the output structure soleq contains two solutions: soleq.el and soleq.ion. In this example we are interested in seeing how mobile ionic carriers influence the device currents so we will use the solution including mobile ionic charge carriers, soleq.ion.

To perform a cyclic voltammogram simulation from 0 to 1.2 to -0.2 to 0 V at 50 $\hbox {mVs}^{-1}$, under 1 sun illumination we call the doCV protocol with the appropriate argument values as detailed in the protocol comments shown in Listing 4.

**Figure:**
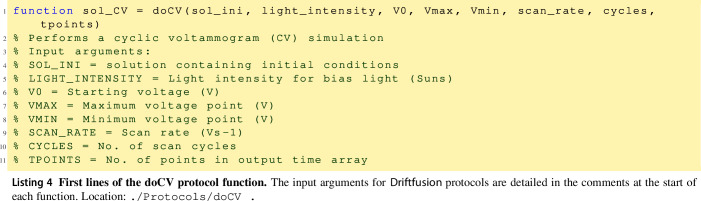

**Figure:**
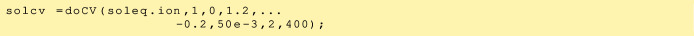

Once a solution has been calculated the different components of the currents can be plotted as a function of voltage using the command:

**Figure:**
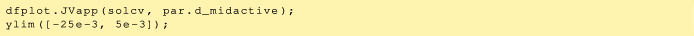

**Fig. 10:**
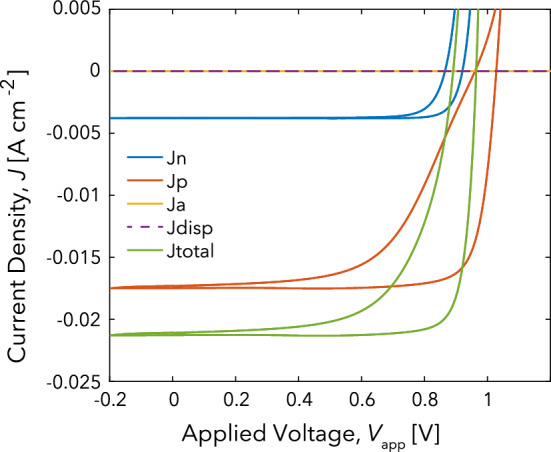
Current–voltage scan results obtained from the cyclic voltammogram protocol (doCV) applied to the default Spiro-OMeTAD/ perovskite/${{TiO}}_2$ solar cell parameters. See the corresponding guide in Sect. 4.9.1 for step-by-step instructions on how to obtain these results

The second argument of dfplot.JVapp is the pre-calculated dependent property d_midactive, the value of which is equal to the position at the midpoint of the active layer of the device. The resulting plot is given in Fig. 10 for reference.

#### How to change the physical model

A detailed step-by-step example of how to modify the physical model to account for the possible effects of photogenerated mobile ionic charge carriers is described in Section S.6 of the Supplemental Information.

### Advanced features

#### Rebuilding device structures and spatial meshes: refresh_device

In some situations where device properties, such as layer widths, are changed in a user-defined script or function, users may need to rebuild the device structures dev and dev_sub, and spatial meshes x and x_sub. To maintain code performance this is not performed automatically using dependent properties within the parameters class definition since non-device-related parameters are frequently changed within protocols. meshgen_x and build_device can be rerun and stored using the new parameter set using the syntax:

**Figure:**
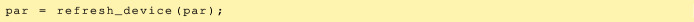

To further illustrate how to use refresh_device, refresh_device_script, a script describing the necessary steps to change the interfacial recombination parameters, is provided in the Scripts folder of the Driftfusion repository.

#### Parallel computing and parameter exploration: explore

Driftfusion’s parameter exploration class explore takes advantage of MATLAB’s parallel computing toolbox to enable multiple simulations to be calculated using a parallel pool. explore_script is an example script demonstrating how to use explore to run an active layer thickness versus light intensity parameter exploration and plot the outputs using explore’s embedded plotting tools.

## Validation against existing models

To verify the numerical accuracy of the simulation, results from Driftfusion were compared against those from two analytical and two numerical models. In Sect. 5.1 current–voltage characteristics obtained using analytical and numerical solutions for a p-n junction solar cell are compared. In Sect. 5.2 the simulation’s time integration is verified by calculating the transient photovoltage response of a single, field-free layer and comparing it to the solution obtained using a zero-dimensional kinetic model. In Sect. 5.3, numerical solutions for three-layer, dual heterojunction devices obtained using Driftfusion are compared with those from the Advanced Semiconductor Analysis (ASA) simulation tool, an established, commercially available package [37]. Finally, in Sect. 5.4, J-V characteristics calculated using Driftfusion for devices dominated by bulk and interfacial recombination processes are compared with those of IonMonger [20], a recently published, free-to-use, mixed ionic-electronic carrier semiconductor device simulator.

The location of the MATLAB scripts and the parameter sets used to obtain the results in this section can be found in the Supplemental Information, Section S.9.

### The depletion approximation for a p-n junction

**Fig. 11:**
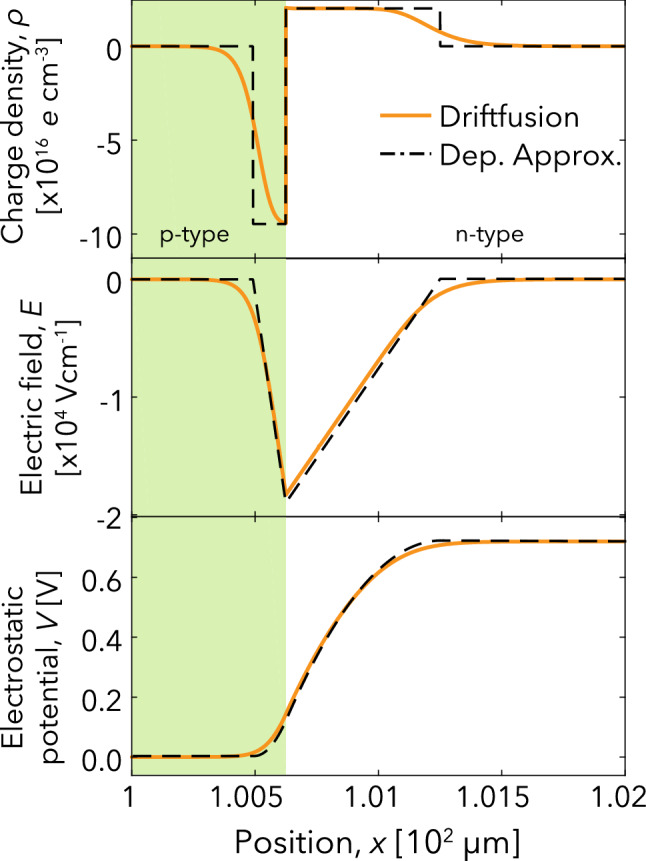
Analytical approximation and numerical solutions of a p-n junction. Solutions obtained from Driftfusion (solid orange curve) and the depletion approximation (Dep. Approx., dashed black curve) for a p-n junction with $E_g = 1.12$ eV and $N_{\mathrm {A}} = 9.47 \times 10^{15}$ $\hbox {cm}^{-3}$, $N_\mathrm {D} = 2.01 \times 10^{15}$ cm^-3^. Green and white regions indicate the n-type and p-type layers, respectively. The complete parameter sets for the simulations are given in Tables S.5 and S.6

*The p-n junction depletion approximation* The Depletion Approximation (DA) allows the continuity equations and Poisson’s Equation (Eqs. 31, 32 and 17) to be solved analytically for a p-n homojunction [45]. Provided that the space charge region at the junction of the device is in a depleted state, the space charge density ρ can be approximated using a step function with magnitude equal to the background doping density (see Fig. 11, top panel). Transport and recombination of free carriers in the depletion region are also neglected within the approximation. Poisson’s equation can then be solved by applying fixed carrier density ($p(x=-\infty ) = p_0$ and $n(x=\infty ) = n_0$) and zero-field boundary conditions to obtain the depletion widths for n- and p-type regions, $w_{\mathrm {n}}$ and $w_{\mathrm {p}}$, yielding [27]

78 $$\begin{aligned} w_{\mathrm {n}}= & {} \dfrac{N_{\mathrm {A}}}{N_{\mathrm {A}} + N_{\mathrm {D}}}\sqrt{\dfrac{2\varepsilon _r \varepsilon _\mathrm {0} V_{\mathrm {bi}}}{q\left( \dfrac{1}{N_{\mathrm {A}}} +\dfrac{1}{N_{\mathrm {D}}} \right) }}, \end{aligned}$$

79 $$\begin{aligned} w_{\mathrm {p}}= & {} \dfrac{N_{\mathrm {D}}}{N_{\mathrm {A}} + N_{\mathrm {D}}}\sqrt{\dfrac{2\varepsilon _r \varepsilon _\mathrm {0} V_{\mathrm {bi}}}{q\left( \dfrac{1}{N_{\mathrm {A}}} +\dfrac{1}{N_{\mathrm {D}}} \right) }}. \end{aligned}$$

Solving the DA for the current flowing across the junction under the assumption that the diffusion length of both carriers is significantly greater than the device thickness ($$L_{n,p}>> d)$$ yields the *Shockley diode equation*:

80 $$\begin{aligned} J = J_{0} \left( e^{{\left( {\frac{{qV_{{{\text {app}}}} }}{{k_{B} T}}} \right) }} - 1 \right) - J_{{SC}}, \end{aligned}$$

where $J_\mathrm {SC}$ and $J_0$ are the short circuit and dark saturation current densities, respectively. Here, we use the convention that a positive applied (forward) bias generates a positive current flowing across the junction.

To make a meaningful comparison between numerical and analytical current–voltage (J-V) characteristics, values for $J_0$ and $J_\mathrm {SC}$ need to be related to input parameters of the simulation. $J_0$ embodies the recombination characteristics of the device; with respect to the contribution to recombination in the quasi-neutral region, $J_0$ can be related to the electron and hole diffusion lengths $L_n$ and $L_p$, minority carrier lifetimes $\tau _n$ and $\tau _p$, and diffusion coefficients $D_n$ and $D_p$, according to [27]

81 $$\begin{aligned} J_0 = \dfrac{q D_p p_{0,n-type}}{L_p} + \dfrac{q D_n n_{0,p-type}}{L_n}, \end{aligned}$$

where $$L_p = \sqrt{\tau _p D_p}$$ and $$L_n = \sqrt{\tau _n D_n}$$.

The material band gap and AM1.5 solar spectrum were used to calculate the theoretical maximum current density $J_{\mathrm {SC,max}}$ and corresponding uniform generation rate throughout the depletion region. Figure S.8 of the Supplemental Information shows the AM1.5 Global Tilt solar spectrum obtained from Ref. [46] used for the calculation.

The limiting short circuit photocurrent for a perfectly absorbing semiconductor of band gap $E_{\mathrm {g}}$ is given by

82 $$\begin{aligned} J_{\mathrm {SC}}(E_{\mathrm {g}}) = q \int _{0}^{\infty } \eta (E_\gamma ) \phi _0(E_\gamma ) dE_\gamma , \end{aligned}$$

where η is the external quantum efficiency [28]. If η=1 for photon energies $E_\gamma \ge E_\mathrm {g}$, and η=0 for $E_\gamma < E_\mathrm {g}$, the maximum theoretically achievable short circuit current for a single junction $J_{\mathrm {SC,max}}$ is given by the integral of $\phi _0$ from the bandgap energy to infinity. Figure S.8 of the Supplemental Information shows the maximum achievable current density $J_{\mathrm {SC,max}}$ over a range of band gap energies. For the comparison we use the bandgap of silicon $E_\mathrm {g} = 1.12$ eV resulting in $J_{\mathrm {SC,max}} = 42.7$ mA cm^-2^.

*Simulation methods* A p-n junction was created in Driftfusion with very thick n- and p-type layers ($\approx 100\ \mu \hbox {m}$, $N_\mathrm {A} = 9.47 \times 10^{15}$ $\hbox {cm}^{-3}$, $N_\mathrm {D} = 2.01 \times 10^{15}$ $\hbox {cm}^{-3}$) to approximate the assumptions and boundary conditions used in the DA. Furthermore, ionic carriers and trapped electronic charges are not included in the simulations in this section. The surface recombination velocity was set to zero for minority carriers at both the left and right system boundaries. A special recombination scheme was implemented using the following simplified first-order expressions:

83 $$\begin{aligned} r= & {} \dfrac{n-n_0}{\tau _n} \quad \mathrm {for} \quad x < d/2 - w_p, \end{aligned}$$

84 $$\begin{aligned} r= & {} \dfrac{p-p_0}{\tau _p} \quad \mathrm {for} \quad x > d/2 + w_n, \end{aligned}$$

where $\tau _n$ and $\tau _p$ are the electron and hole lifetimes, respectively. For simplicity, $\tau _n$ and $\tau _p$ were set equal to one another and, for consistency with the DA, recombination was switched off in the depletion region.

To convert the value obtained for $J_{\mathrm {SC,max}}$ into a uniform carrier generation rate, the short circuit flux density ($j_{\mathrm {SC,max}} = J_{\mathrm {SC,max}}/q$) was divided by the depletion region thickness $d_{\mathrm {DR}}$, yielding $g_0 = j_{\mathrm {SC,max}}/d_{\mathrm {DR}}$. The complete parameter sets for the simulations in this section are given in Tables S.5 and S.6.

*Results* Both the analytical and numerical solutions for the space charge density, electric field, and electric potential are shown in Fig. 11: The space charge widths, field strength and potential profiles all show good agreement.

**Fig. 12:**
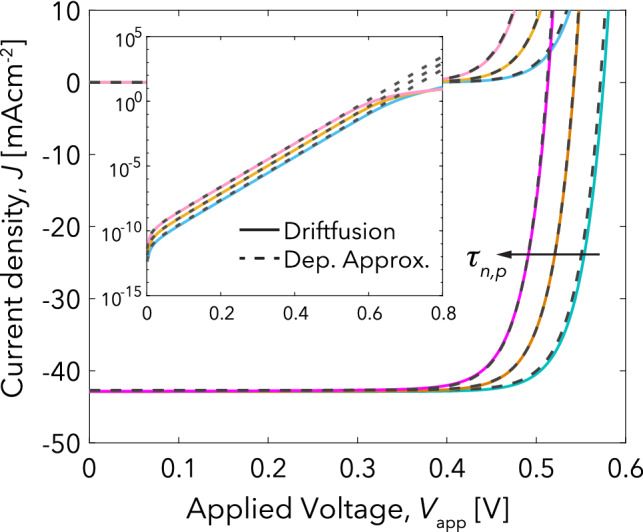
Comparison of current–voltage characteristics obtained using Driftfusion and the Depletion Approximation for a p-n junction. Current–voltage characteristics for numerical and analytical solutions for a p-n junction with $\tau _n = \tau _p = 10^{-6}, 10^{-7}$, and $10^{-8}$ s. Results from Driftfusion and the Depletion Approximation (Dep. Approx.) are denoted by solid and dashed lines, respectively. The complete parameter sets for the simulations are given in Tables S.5 and S.6. The dark currents are shown on a logarithmic scale in the inset

Figure 12 shows the light and dark current–voltage curves for the analytical solution obtained using equation 80, as compared to the numerical solutions from Driftfusion for three values of $\tau _{n,p}$. For $\tau _{n,p} = 10^{-6}s$ and $\tau _{n,p} = 10^{-7}s$ the agreement is very good, even at current densities as low as $10^{-14}$ mA $\hbox {cm}^{-2}$. The solutions begin to diverge with $\tau _{n,p} = 10^{-8}$ s, for which $L_{n,p} = 2.3$ $\mu \hbox {m}$ such that $L_{n,p}<< d$ and the underlying assumptions of the DA break down. The deviation from the ideal model in Driftfusion at higher current densities (Fig. 12, inset) is expected due to the absence of series resistance in the DA and because the capacitance of the space charge regions can no longer be approximated using the DA at applied voltages close to, and beyond, the built-in voltage of the junction.

### Transient photovoltage response of a single layer field-free device

*Analytical methods* To verify the time-dependence of the solution from Driftfusion, the transient photovoltage (TPV) response for a field-free slab of intrinsic semiconductor was calculated numerically and compared to results from a zero-dimensional (0-D) kinetic model. During a TPV experiment the device is illuminated at open circuit with a constant bias light and pulsed with an optical excitation source to produce a small additional photovoltage $\varDelta V_{\mathrm{OC}}$. As detailed in Supplemental Information, Sect. S.9.2, it can be shown using a kinetic model that $\varDelta V_{\mathrm{OC}}$ is given by:

85 $$\begin{aligned}&\varDelta V_\mathrm {OC}= \dfrac{2k_\mathrm {B}T}{q n_\mathrm {OC}}\dfrac{\varDelta g}{k_\mathrm {TPV}} \left( 1 - e^{-k_\mathrm {TPV}(t + t_\mathrm {pulse})}\right) \nonumber \\&\mathrm {for}\ -t_\mathrm {pulse} <t \leqslant 0, \end{aligned}$$

86 $$\begin{aligned}&\varDelta V_\mathrm {OC}= \dfrac{2k_\mathrm {B}T}{q n_\mathrm {OC}}e^{-k_\mathrm {TPV}t} \quad \mathrm {for}\ t >0, \end{aligned}$$

where $t_{\mathrm {pulse}}$ is the length of the laser pulse, Δg is the additional uniform volumetric generation rate due to the excitation pulse, $n_\mathrm {OC}$ is the steady-state open circuit carrier density, and $k_\mathrm {TPV}$ is the decay rate constant of the TPV signal.

*Simulation methods* A 100 nm field-free single layer of semiconductor with $E_g = 1.6$ eV was simulated in Driftfusion with the zero flux density boundary conditions for electronic carriers representing perfect blocking contacts. The constant and uniform volumetric generation rate was set to $g_0 = 1.89\times 10^{21}$ $\hbox {cm}^{-3}$ $\hbox {s}^{-1}$ =1 sun equivalent, based on the integrated photon flux density for the $\hbox {AM}1.5\hbox {G}$ solar spectrum and a step function absorption. In this special case the splitting of the quasi-Fermi levels in the simulation is solely attributable to changes in chemical potential. It should be noted however that, for instances where an electric field is present in the device, these boundary conditions will not represent an open circuit condition and a high value of external series resistance $R_s$ or a mirrored cell approach (see Ref. [18] for details) should be used instead. The second-order band-to-band recombination coefficient was set to $B = 10^{-10}$ $\hbox {cm}^3\hbox {s}^{-1}$ and SRH recombination was switched off. Since the underlying kinetic theory demands that the TPV perturbation must be small such that the additional carrier density $\varDelta n<< n_\mathrm {OC}$, we used $t_{\mathrm {pulse}} = 1\ \mu \hbox {s}$ and set the pulse intensity equivalent to be 20% of the bias light intensity. The complete parameter sets for the simulations are given in Tables S.7 and S.8.

**Fig. 13:**
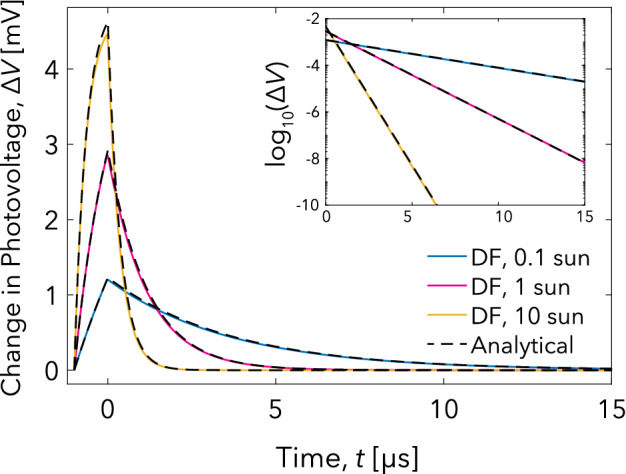
Zero-dimensional kinetic model solution versus 1-D numerical drift-diffusion simulations for the transient photovoltage response of a single layer of semiconductor. Change in photovoltage as a function of time calculated using Driftfusion (DF, solid curves) and a zero-dimensional kinetic model (Analytical, black dashed curves) at bias light intensities of 0.1, 1, and 10 sun equivalent. The inset shows the results on a log vertical scale. The complete parameter sets for the simulations are given in Tables S.7 and S.8

**Table 3:** Summary of the key simulation parameters for comparison of Driftfusion with ASA. CB and VB denote the conduction and valence bands, respectively

Parameterset	Description	Built-in voltage (V)	Active layer thickness (nm)	CB and VB effective density of states ($\hbox {cm}^{-3}$)		
				HTL	Absorber	ETL
1a	3-layer device based on MAPI active layer	1.05	400	$10^{19}$	$10^{18}$	$10^{19}$
1b	As 1a with thinner active layer	1.05	200	$10^{19}$	$10^{18}$	$10^{19}$
2a	Randomised layer properties	0.6	200	$10^{19}$	$10^{18}$	$10^{20}$
2b	As 2a with uniform eDOS all layers	0.6	200	$10^{18}$	$10^{18}$	$10^{18}$

*Results* A comparison of the results from the analytical and simulation models for bias light intensities of 0.1, 1, and 10 sun equivalent is shown in Fig. 13. The steady-state charge carrier densities and open circuit voltages obtained using Driftfusion agreed to within 10 decimal places with the analytical values calculated using Equations S.15 and S.14 ($n = 4.35\times 10^{15}$ $\hbox {cm}^{-3}$, $V_\mathrm {OC} = 1.08$ V at 1 sun equivalent). The transient photovoltage perturbations also behaved as predicted, with the rate constants extracted from fitting the TPV decays correct to within 3 significant figures of the values calculated using the analytical expression in Eq. 86.

### Numerical solution for three-layer devices with electronic carriers

*Simulation methods* To verify that the discrete treatment of the interfaces employed in Driftfusion produces equivalent results to models that use abrupt interfaces, a HTL/absorber/ETL device was simulated using both Driftfusion and the Advanced Semiconductor Analysis (ASA) simulation tool [37]. To maintain consistent layer dimensions the linear grid spacing for the ASA simulations and the interface thickness in Driftfusion were set to be 1 nm.

The base material parameters for the active layer were based loosely on those for a perovskite material excluding mobile ionic charge. The parameters for the contact layers were not chosen to simulate real materials but rather to produce a large built-in potential and to vary as many properties as possible including the layer thickness, dielectric constants, recombination coefficients, mobilities, etc. For optical generation the Beer–Lambert option (without back contact reflection) was chosen and the same optical constant and incident photon flux density spectrum data were used in both simulators. Four different parameter sets (PS) were compared, the key differences for which are summarised in the first four columns of Table 3. The complete parameter sets for the simulated devices are given in Tables S.9–S.12.

*Results: Beer–Lambert optical model* The results for the integrated generation rate profiles are shown in Figure S.10. Despite the wavelength-dependent generation rates appearing to be very closely matched between the two simulators (Figure S.11), the integration across photon energies resulted in marginally different generation profiles in the two simulations corresponding to a total difference in generation current of 1.24 mA $\hbox {cm}^{-2}$. As a means to ensure that the input generation profiles were identical, the generation profile from ASA was inserted into the Driftfusion parameters objects using the method described in Sect. 4.2.7.

*Results: Current–voltage characteristics* To compare the current outputs from the different simulation tools, current–voltage scans were performed from $V_{\mathrm {app}} = 0$ to 1.3 V. Since ASA solves for the steady-state current, the J-V scan rate was set to be $k_{\mathrm {scan}} = 10^{-10}$ V $\hbox {s}^{-1}$ in Driftfusion to minimise contributions from the displacement current.

**Fig. 14:**
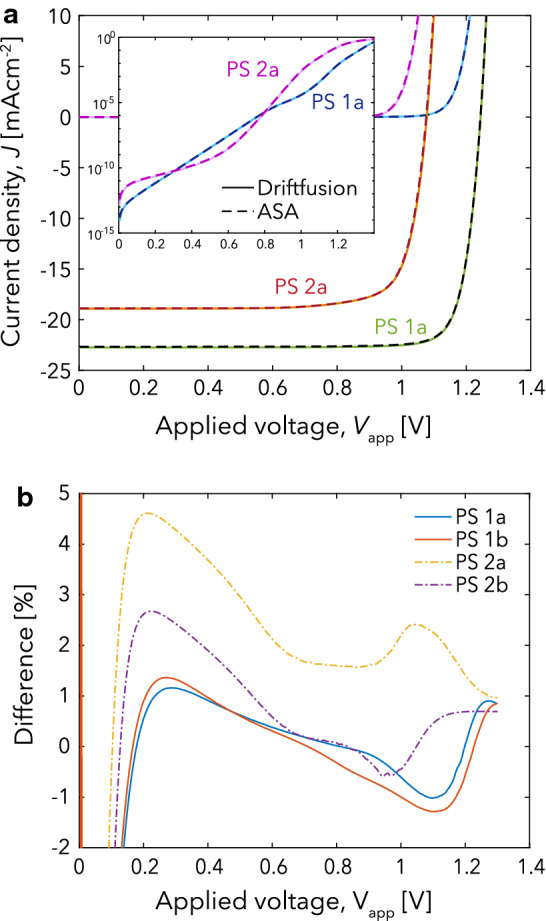
Comparison of dark and light current–voltage characteristics calculated by Driftfusion and ASA **a** Current–voltage characteristics for Parameter Sets (PS) $1\hbox {a}$ and $2\hbox {a}$. Inset dark currents shown on log scale. Dashed and solid lines indicate the current calculated using Driftfusion and ASA, respectively. **b** Percentage difference between dark current calculated using Driftfusion and ASA for the 4 different parameter sets investigated. The complete parameter sets for the simulations are given in Tables S.9 - S.12

Figure 14a shows a comparison of the J-V characteristics obtained from the two simulation tools for Parameter Sets (PS) 1a and 2a. While the different parameter sets result in distinctly different characteristics for the two devices, the data show excellent agreement between the two simulators despite the significant difference in discretisation schemes and interface treatment.

**Fig. 15:**
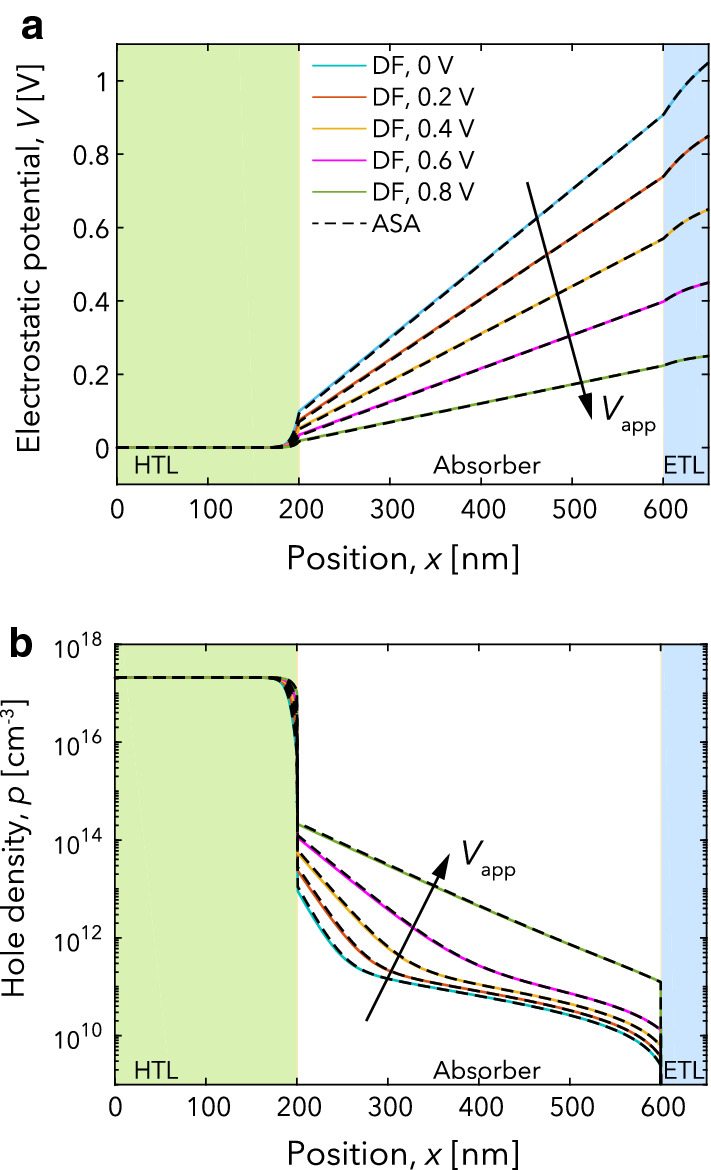
Comparison of the electrostatic potential and hole density profiles during a *J*-*V* scan for a three-layer device calculated by Driftfusion (solid lines) and ASA (dashed lines). Corresponding electron densities are given in Supplemental Information Figure S.15

Closer examination of the results from the two simulators (Fig. 14b) reveals that, the percentage difference (calculated as $100 \times (J_\mathrm {ASA} - J_\mathrm {DF})/J_\mathrm {ASA}$) for PS 1a is on the order of 1 % for current densities beyond $J = 10^{-12}$ A $\hbox {cm}^{-2}$. Halving the active layer thickness has little impact on this difference (PS 1b). The percentage difference calculated for PS 2a, however, is much greater, with a maximum of ≈5 % for current densities $J > 10^{-12}$ mA $\hbox {cm}^{-2}$. The larger difference between the two simulators in this instance can be attributed to a change of over 7 orders of magnitude in the electron density at the absorber-ETL interface (Figure S.14) due to both a transition in the conduction band eDOS from $N_\mathrm {CB} = 10^{18}$ to $10^{20}$ $\hbox {cm}^{-3}$ and a change in the conduction band energy of 0.3 eV. Under these circumstances the difference in discretisation schemes between the two tools becomes apparent: the linear discretisation method used in the PDEPE and Driftfusion cannot calculate the change in carrier densities within the interfaces to as high a degree of accuracy as the internal boundary conditions used in ASA (see Section S.8.1 for further details). The deviation can be reduced significantly, however, by using a uniform eDOS of $N_\mathrm {CB} = 10^{18}$ $\hbox {cm}^{-3}$ across all layers as the results for PS 2b show. With respect to device characteristics, despite the percentage difference in results for PS 2a, the key metrics of the J-V curve such as the $V_\mathrm {OC}$, ideality factor and fill-factor are all preserved. Figure 15 shows a comparison of the electrostatic potential and hole density profiles calculated using Driftfusion and ASA for PS 1a under illumination at increasing applied bias. The electron density is given in the Supplemental Information, Figure S.15. The agreement here between the solutions is excellent taking into consideration the difference in treatment of the interfaces between the two simulators.

### Numerical solution for three-layer devices with electronic and mobile ionic carriers

Courtier et al. recently published IonMonger, [20] a three-layer (HTL/absorber/ETL) drift-diffusion simulation tool for modelling perovskite solar cells which solves for coupled electron, hole and cation carrier distributions. In contrast to Driftfusion, IonMonger uses abrupt interfaces and solves carriers and the electrostatic potential for each of the three device layers simultaneously. The advantage to this approach is that boundary conditions can be established between the absorber and transport layers, such that interfacial recombination between electrons from one material and holes from another can be evaluated at the same grid point. The disadvantage is that 8 variables are solved for simultaneously (minority carriers are not included in the transport regions) as opposed to 4 in Driftfusion, for an equivalent three-layer device with a single mobile ionic species. Here, we compare results obtained from Driftfusion with those from IonMonger for devices with similar parameter sets.

*Methods* We modelled three layer perovskite HTL/absorber/ETL solar cells with electronic and mobile ionic carriers in the absorber layer and electronic carriers only in the HTL and ETL using IonMonger and Driftfusion. For the IonMonger simulations only holes were solved for within the HTL and electrons within the ETL. For the Driftfusion simulations all carriers were solved for in all regions with the ionic carrier mobility set to zero in the HTL, ETL and interfacial regions. Since the ionic carriers are modelled as inert and their charge is compensated by a static background charge density (see Eq. 17) they have no effect in these regions. For the comparison we ran J-V scan simulations at a variety of scan speeds using similar parameter sets to those published in Ref. [8]: a device dominated by bulk recombination and a device dominated by interfacial recombination using the volumetric surface recombination scheme described in Sect. 3.7.3.

**Fig. 16:**
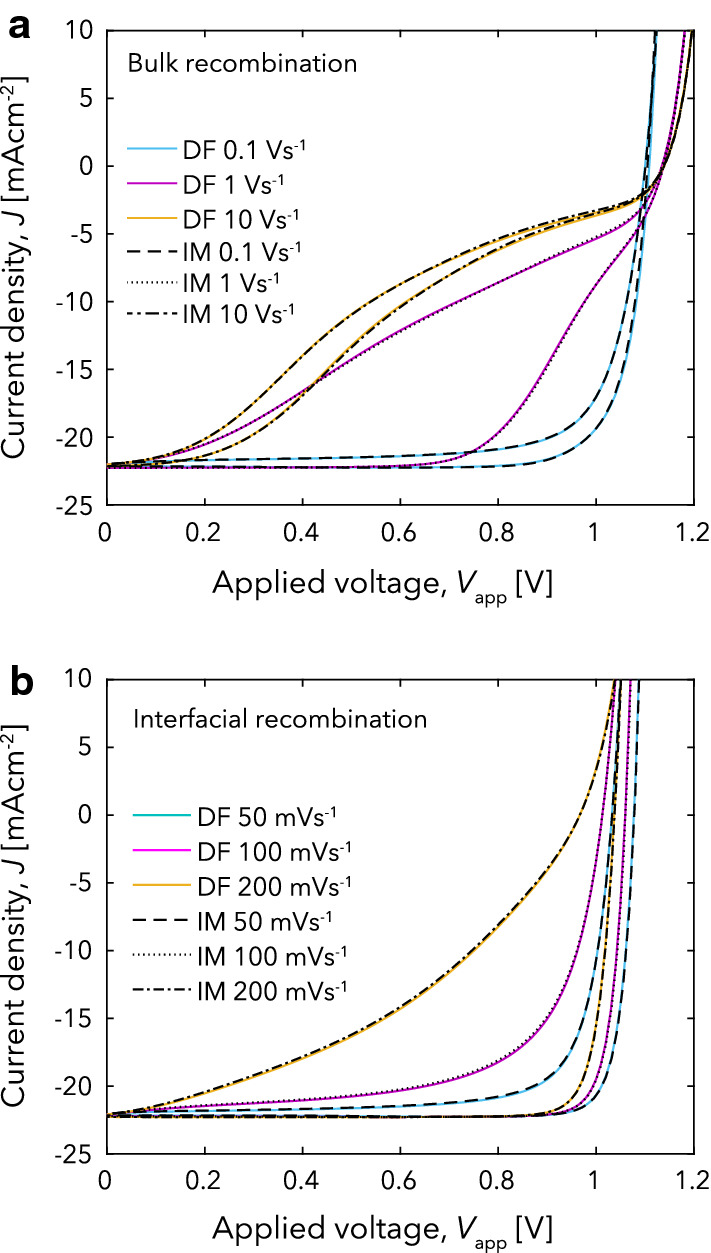
Comparison of results calculated using Driftfusion and IonMonger for three-layer solar cells including mobile ionic carriers in the absorber layer. **a** Current–voltage scans at $k_\mathrm {scan}=0.1$, 1, and 10 V $\hbox {s}^{-1}$ for a device dominated by bulk recombination. **b** Current–voltage scans at $k_\mathrm {scan}=50$, 100, and 200 mV $\hbox {s}^{-1}$ for a device dominated by interfacial recombination using the scheme described in Sect. 3.7.3. The complete parameter sets for the simulations are given in Tables S.13– S.15

*Results: Current–voltage characteristics* Fig. 16a, b shows the J-V results from Driftfusion (solid coloured curves) and IonMonger (black dashed curves) for the bulk and interfacial recombination dominated devices at varying voltage scan rates. The electrostatic potential profile, ionic carrier accumulation and electronic carrier profiles during the 1 V $\hbox {s}^{-1}$ forward scan for the bulk recombination device, and at 100 mV $\hbox {s}^{-1}$ for in the interfacial recombination dominated device, at increasing applied bias are given in the Supplemental Information Figures S.21 and S.22, respectively. For both sets of parameters the results obtained from the two simulators are very similar with marginal differences in the calculated current outputs. This variance arises principally from the treatment of electronic currents across interfaces, differences in the spatial mesh, and calculation of ionic carrier densities throughout all layers of the device in Driftfusion as opposed to IonMonger, which introduces small additional integration errors into the space charge density (Supplemental Information Figure S.20). For the device dominated by interfacial recombination (Fig. 16b), additional errors are also introduced by the volumetric surface recombination scheme. Figure S.23 shows that, while the scheme is self-consistent, differences arise from the surface carrier densities (specifically the electron density at the active layer-HTL interface). These differences are accentuated by increasing the energetic barriers to minority carriers from 0.4 to 0.8 eV (Eqs. 37 and 38) as shown in Supplemental Information Figure S.24. The differences can however be reduced by increasing the interface thickness and using a larger number of interface mesh points (Figure S.24). To this end, consistency with established analytical models that use abrupt interfaces is somewhat sacrificed in Driftfusion in favour of greater flexibility, which enables the physical models to be easily edited, devices with any number of material layers to be simulated and a range of interface-specific properties and grading functions to be specified.

We have verified Driftfusion against two analytical and two existing numerical models and found that in all cases the calculated results are in good agreement. The results show that the discrete interface approach produces results comparable to models with abrupt interfaces, albeit with marginal errors introduced attributable to the combination of the interface treatment and the linear discretisation scheme used in Driftfusion.

## Conclusions

We have developed an efficient and powerful one-dimensional drift-diffusion simulation tool for modelling semiconductor devices with mixed ionic-electronic conducting layers based on MATLAB’s pdepe toolbox. Distinct from existing codes, in addition to electronic carriers, Driftfusion can include up to two ionic carrier species and virtually any number of material layers. This flexibility is made possible by a discrete interlayer interface approach whereby the material properties can be graded between two adjoining semiconductor layers. This method has the added advantage that interface-specific properties can easily be specified.

The default physical models underlying the simulation were described and analytical approximations to the carrier densities and fluxes within the interfacial regions were stated. Using these solutions, a method for approximating surface recombination within interfacial regions using a volumetric recombination scheme was derived.

The system architecture was presented and the processes by which users can define device properties and change the simulation’s physical model were outlined. Protocol functions, determining time-dependent voltage and light conditions, were described as well as solution structures and how to use built-in analysis and plotting functions to calculate and visualise outputs from the simulation solutions. A step-by-step guide was presented detailing how to create a device, find an equilibrium solution, and calculate current–voltage characteristics using a cyclic voltammogram protocol. Lastly, advanced features, including how to calculate multiple solutions in parallel, were briefly introduced.

Driftfusion was verified by comparing calculated solutions with two analytical and two existing numerical models which tested different aspects of the simulation. In all cases the agreement was good and the general device behaviour was reproduced. The discrete treatment of the interfaces resulted in small variations in the calculated currents as compared to other simulators using abrupt interfaces at layer boundaries.

The ease with which the underlying models can be changed, and new material layers introduced, places Driftfusion in a unique space compared to other free-to-use simulation codes for which an intricate knowledge of the numerical mechanics is required to adapt the physical models and device architecture. By making Driftfusion both accessible and free-to-use our hope is that this work will advance our collective understanding of mixed ionic-electronic conducting materials and devices.

## Supplementary Information

Below is the link to the electronic supplementary material.

Supplementary file 1 (pdf 7669 KB)

## Data Availability

The data presented in this work are available at reasonable request from the authors.

## Footnotes

1 Based on an ion density of $1.6\times 10^{19}$ $\hbox {cm}^{-3}$.

2 It follows that $E_{{ {CB}},0}$ is only dependent on position, while $E_{ {CB}}$ is also time-dependent.

3 For simplicity, the temperature-dependence of $N_\mathrm {CB}$ and $N_\mathrm {VB}$ has been omitted from the equations herein and it should further be noted that this temperature dependence is not explicitly dealt with in this release of Driftfusion.

4 $$N_\mathrm {CB} = 2(2\pi m_e^* k_{\mathrm {B}} T/h^2)^{3/2}$$ and $$N_\mathrm {VB} = 2(2\pi m_h^* k_{\mathrm {B}} T/h^2)^{3/2}$$

5 For clarity only charge neutrality of the Schottky defect terms is guaranteed, this does not include the contribution from dopant atoms, which may not be compensated by electronic carriers in regions where an electric field is present.

6 We note that while either model may be closer in one or more aspects to the real situation, both lack a comprehensive quantum mechanical treatment.

7 Note that at the time of writing this method is not stable for all input parameter sets.

## Declarations

### Code availability

The simulation code is freely available at: https://github.com/barnesgroupICL/Driftfusion. https://doi.org/10.5281/zenodo.6045592
