# Supplementary material for: Driftfusion: an open source code for simulating ordered semiconductor devices with mixed ionic-electronic conducting materials in one dimension
Source: J Comput Electron. 2022 May 25;21(4):960–91. doi: 10.1007/s10825-021-01827-z (PMC13476298; doi:10.1007/s10825-021-01827-z)
Supplement: Supplementary file 1 [file 10825_2021_1827_MOESM1_ESM.pdf]

manuscript No.
(will be inserted by the editor)

Supplemental information for Driftfusion

An open source code for simulating ordered semiconductor devices with mixed ionic-electronic conducting materials in one dimension

Philip Calado[1] · Ilario Gelmetti[2] · Benjamin Hilton[1] · Mohammed Azzouzi[1] · Jenny Nelson[1] · Piers R. F. Barnes[1]

S.1 Universal constants, abbreviations and symbols

Table S.1 Universal constants.

Constant Name	Symbol	Driftfusion Property Name	Value
Boltzmann constant	k_{B}	`kB`	8.62×10^{-5} eV K^{-1}
Charge of an electron	q	`e`	1.60×10^{-19} C
Permittivity of free space	ε_0	`epp0`	8.85×10^{-12} m^{-3}kg^{-1}s^{4}A^{2}

UK Engineering and Physical Sciences Research Council grant No. EP/J002305/1, EP/M025020/1, EP/M014797/1, EP/R020574/1, EP/R023581/1, EP/L016702/1, EP/T028513/1 (ATIP) and European Union's Horizon 2020 research and innovation program grant agreement No. 742708.

P. Calado
E-mail: p.calado13@imperial.ac.uk
[1] Department of Physics, Imperial College London, London SW7 2AZ, UK.
[2] Institute of Chemical Research of Catalonia (ICIQ), Barcelona Institute of Science and Technology (BIST), Avda. Paisos Catalans 16, 43007 Tarragona, Spain.

Table S.2 Table of symbols. †The absorption and photon flux data are loaded directly from libraries according to the layer names defined in `stack` and the choice of light source using the properties `light_source1` and `light_source2`. *Solely for the `Methods-depletion-approximation` branch. **For the .csv file the name `thickness` can also be used. For a complete description of all user-defined and dependent properties please see the comments in the parameters class `pc`. For information on calculated outputs see the analysis class `dfana`. VSR ~~indicates~~ denotes auto-generated coefficients used in the volumetric surface recombination scheme.

Symbol	Driftfusion Variable Name	Variable type	Name	Unit
α_{abs}	†	-	Absorption coefficient	cm^{-1}
γ	-	-	Reaction order	
ε_{r}	`epp`	Property	Relative permittivity	
η	-	-	External quantum efficiency	
κ	-	-	Reflectance	-
λ	-	-	Wavelength	nm
μ_e	`mu_n`	Property	Electron mobility	$\mathrm{cm}^2\ \mathrm{V}^{-1}\ \mathrm{s}^{-1}$
μ_h	`mu_p`	Property	Hole mobility	$\mathrm{cm}^2\ \mathrm{V}^{-1}\ \mathrm{s}^{-1}$
μ_a	`mu_a`	Property	Anion mobility	$\mathrm{cm}^2\ \mathrm{V}^{-1}\ \mathrm{s}^{-1}$
μ_c	`mu_c`	Property	Cation mobility	$\mathrm{cm}^2\ \mathrm{V}^{-1}\ \mathrm{s}^{-1}$
ρ	`rho`	Calculated output	Charge density	cm^{-3}
σ	-	-	Conductivity	$\mathrm{S\ cm}^{-1}$
τ_n, SRH	`taun`	Property	SRH electron lifetime	s
τ_p, SRH	`taup`	Property	SRH hole lifetime	s
τ_n, vsr	`taun_vsr`	Dependent property	VSR electron lifetime,	s
τ_p, vsr	`taup_vsr`	Dependent property	VSR hole lifetime	s
τ_n	`taun`*	Property	Electron first order lifetime	s
τ_p	`taup`*	Property	Hole first order lifetime	s
~~v_n sn Property Electron interfacial surface recombination velocity cms^{-1} v_p sp Property Hole interfacial surface recombination velocity cms^{-1}~~ ϕ	†	-	Photon flux	$\mathrm{cm}^{-2}\mathrm{s}^{-1}$
Φ_{EA}	`EA`	Property	Electron affinity	eV
Φ_{IP}	`IP`	Property	Ionisation potential	eV
Φ_l	`Phi_l`	Property	Left-hand electrode workfunction	eV
Φ_r	`Phi_r`	Property	Right-hand electrode workfunction	eV
a	`a`	Solution variable	Mobile anion carrier density	cm^{-3}
a_{max}	`a_max`	Property	Limiting anion density	cm^{-3}
c	`c`	Solution variable	Mobile cation carrier density	cm^{-3}
c_{max}	`c_max`	Property	Limiting cation density	cm^{-3}
d	`d`**	Property	Layer thickness	cm
d_{dev}	`dcum(end)`	Dependent property	Device thickness	cm
d_{DR}	-	-	Depletion region thickness	cm
g	`gx1`,`gx2`, `gx`	Property	Volumetric generation rate	$\mathrm{cm}^{-3}\ \mathrm{s}^{-1}$
g_0	`g0`	Property	Uniform generation rate	$\mathrm{cm}^{-3}\ \mathrm{s}^{-1}$
j	`j`	Calculated output	Flux density	$\mathrm{cm}^{-2}\ \mathrm{s}^{-1}$
k_γ	-	-	Rate coefficient for reaction order γ	Variable
B	`B`	Property	Band-to-band recombination rate coefficient	$\mathrm{cm}^{-3}\ \mathrm{s}^{-1}$
k_{scan}	-	-	Current-voltage scan rate	Vs^{-1}
k_{TPV}	-	-	TPV decay rate coefficient	s^{-1}
n	`n`	Solution variable	Electron carrier density	cm^{-3}
n_0	`n0`	Dependent property	Equilibrium electron density	cm^{-3}
n_{id}	-	-	Diode ideality factor	
n_{i}	`ni`	Dependent property	Intrinsic carrier density	cm^{-3}
n_{t}	`nt`	Dependent property	SRH electron trap parameter	cm^{-3}
p	`p`	Solution variable	Hole carrier density	cm^{-3}
p_0	`p0`	Dependent property	Equilibrium hole density	cm^{-3}
p_{t}	`pt`	Dependent property	SRH hole trap parameter	cm^{-3}
r	`r`	Calculated output	Volumetric recombination rate	$\mathrm{cm}^{-3}\ \mathrm{s}^{-1}$
r_{btb}	`r.btb`	Calculated output	Band-to-band recombination rate	$\mathrm{cm}^{-3}\ \mathrm{s}^{-1}$
r_{SRH}	`r.srh`	Calculated output	SRH recombination rate	$\mathrm{cm}^{-3}\ \mathrm{s}^{-1}$
r_{vsr}	`r.vsr`	Calculated output	Volumetric surface recombination rate	$\mathrm{cm}^{-3}\ \mathrm{s}^{-1}$
s_n	`sn`	Property	Electron surface recombination velocity at interfaces	$\mathrm{cm\ s}^{-1}$

Table S.3 **Table of symbols continued.** For a complete description of all user-defined and dependent properties please see the comments in the parameters class `pc`. For information on calculated outputs see the analysis class `dfana`.

**Symbol**	**Driftfusion Property Name**	**Variable type**	**Name**	**Unit**
t	`t`	Property	Time	s
w_{n}	-	-	n-type depletion width	cm
w_{p}	-	-	p-type depletion width	cm
x	`x`	Property	Position	cm
x_n	-	-	Translated spatial co-ordinate for electron distribution within the interfacial regions	cm
x_p	-	-	Translated spatial co-ordinate for hole distribution within the interfacial regions	cm
y	-	-	Generic carrier concentration	cm^{-3}
z	`z`	Property	Integer charge state	-
~~**Table of symbols continued.** For a complete description of all user-defined and dependent properties please see the comments in the parameters class `pc`. For information on calculated outputs see the analysis class `dfana`. **Symbol Driftfusion Property Name Variable type Name Unit**~~ D_n	`Dn`	Solver variable	Electron diffusion coefficient	$\mathrm{cm}^2\ \mathrm{s}^{-1}$
D_p	`Dp`	Solver variable	Hole diffusion coefficient	$\mathrm{cm}^2\ \mathrm{s}^{-1}$
E	-	-	Energy	eV
E_γ	-	-	Photon energy	eV
E_{CB}	`Ec`	Property	Conduction band energy	eV
E_{VB}	`Ev`	Property	Valence band energy	eV
E_{Fi}	`Efi`	Dependent property	Intrinsic Fermi energy	eV
E_{F0}	`E0`	Property	Equilibrium Fermi level	eV
E_{Fn}	`Efn`	Calculated output	Electron quasi Fermi level	eV
E_{Fp}	`Efp`	Calculated output	Hole quasi Fermi level	eV
E_{vac}	`Evac`	Calculated output	Vacuum energy	eV
E_{g}	`Eg`	Dependent property	Bandgap energy	eV
E_{t}	`Et`	Property	SRH trap energy	eV
F	`F`	Calculated output	Electric field	$\mathrm{V\ cm}^{-1}$
J	`J`	Calculated output	Current density	$\mathrm{A\ cm}^{-2}$
J_{SC}	`Jsc`	Calculated output	Short circuit current density	$\mathrm{A\ cm}^{-2}$
J_0	-	-	Dark saturation current	$\mathrm{A\ cm}^{-2}$
$J_{0,\mathrm{rad}}$	-	-	Black body recombination current	$\mathrm{A\ cm}^{-2}$
L	-	-	Diffusion length	cm
$\bar{L}$	-	-	Mean free path length	cm
N_{ani}	`Nani`	Property	Intrinsic anion density	cm^{-3}
N_{cat}	`Ncat`	Property	Intrinsic cation density	cm^{-3}
N_{A}	`NA`	Dependent property	Acceptor density	cm^{-3}
N_{D}	`ND`	Dependent property	Donor density	cm^{-3}
N_{CB}	`Ncb`	Property	Conduction band effective density of states	cm^{-3}
N_{VB}	`Nvb`	Property	Valence band effective density of	cm^{-3}

Table S.4 Abbreviations.

**General**	
ASA	Advanced Semiconductor Analysis
AM	Air Mass
CB	Conduction Band
DD	Drift-Diffusion
DSSC	Dye Sensitised Solar Cell
eDOS	Effective density of states
EQE	External Quantum Efficiency
ETL	Electron Transport Layer
FF	Fill Factor
HTL	Hole transport Layer
J-V	Current density-Voltage Scan
LED	Light Emitting Diode
OPV	Organic Photovoltaic
PCE	Power Conversion Efficiency
PDEPE	Partial Differential Equation solver for Parabolic and Elliptic equations
PSC	Perovskite Solar Cell
PV	Photovoltaics
QFL	Quasi Fermi Level
SRH	Shockley-Read-Hall
VB	Valence Band
**Materials**	
ITO	Indium tin oxide
MAPI	Methylammonium lead iodide
mp-TiO_2	Mesoporous TiO_2
PCBM	Phenyl-C61-butyric acid methyl ester
Spiro-OMeTAD	2,2’,7,7’-Tetrakis[N,N-di(4-methoxyphenyl)amino]-9,9’ -spirobifluorene

S.2 Physical principles

S.2.1 Carrier flux densities

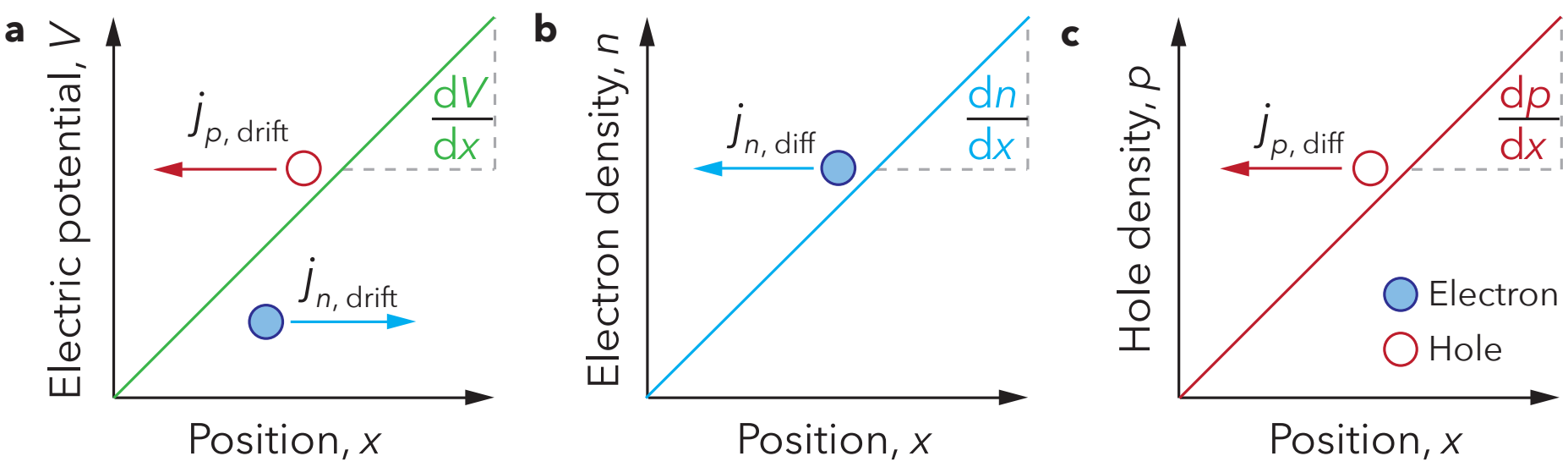

Fig. S.1 Electron and hole carrier flux densities. Direction of electron j_n and hole j_p carrier flux densities in response to positive gradients in (**a**) the electric field potential (note: $F = -\frac{dV}{dx}$), and (**b**) electron and (**c**) hole carrier densities. The subscripts ‘drift’ and ‘diff’ denote drift and diffusion flux densities respectively. Analogous flux densities can be drawn for mobile ionic species by substituting cations for holes and anions for electrons.

S.2.2 Energy level diagrams

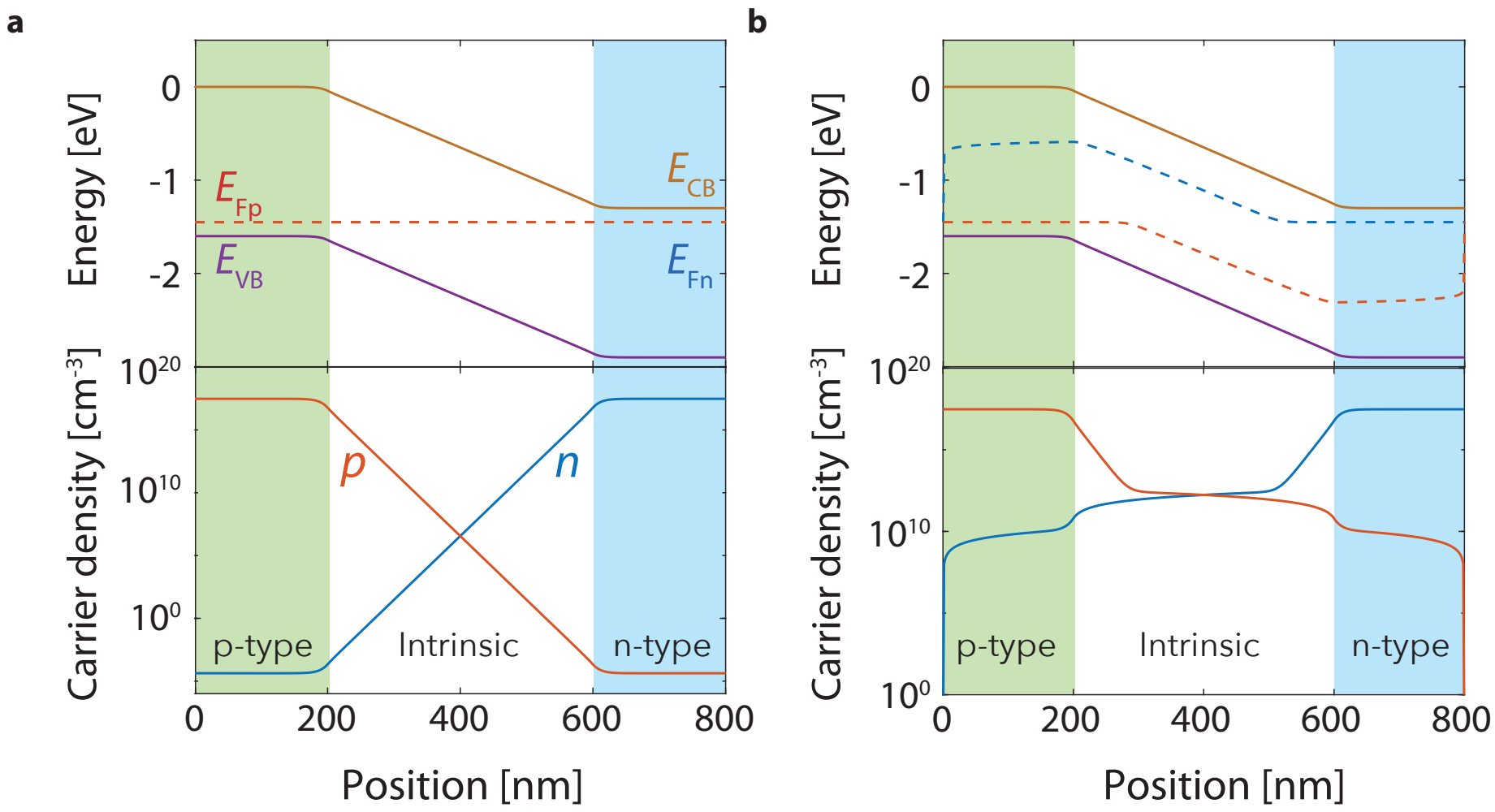

Fig. S.2 Short circuit equilibrium and illuminated p-i-n energy level diagrams and charge densities. (**a**) A simulated *p*-*i*-*n* structure at thermal equilibrium. p and n-type regions are shaded in green and blue respectively. The built-in field predominantly drops across the active layer (white region) while the chemical potential gradient is equal and opposite accounting for the same slope in the n and p profiles plotted on a logarithmic scale. This results in flat quasi-Fermi levels (QFLs) for both electrons and holes indicating that no current is flowing. (**b**) The same device at short circuit under optical bias: The QFLs split due to population of the valence and conduction bands with photoexcitated electrons and holes. Here, the Fermi level gradients indicate that net electron and hole currents are flowing in the device.

Energy level diagrams[1] provide a convenient method for visualising the spatial solution of the simulation at a single time point. The electron and hole quasi-Fermi levels (QFLs), and conduction and valence band energies at each location within the device are plotted with respect to E_{vac}. The slope of the bands indicates the strength of the electric field. The position of the QFLs with respect to the bands gives an indication of the occupation state of the bands, while the slope of the QFLs determines the direction and strength of the overall electrochemical driving force. Figure S.2a and S.2b shows example energy level diagrams of a *p*-*i*-*n* structure at equilibrium and short circuit under illumination.

[1] The terms 'band diagrams' and 'energy level diagrams' are often used interchangeably although we avoid this terminology here to prevent confusion with band structure diagrams (plots of energy versus crystal momentum).

S.3 Steady-state analytical solution for electronic carrier ~~density solutions~~ densities within the interfacial regions

S.3.1 Solution

To examine how the carrier densities change within the interfacial regions we begin with the continuity equation for electrons and assume no generation such that:

$$\frac{\partial n(x,t)}{\partial t} = -\frac{\partial j_n(x,t)}{\partial x} - r_n(x,t) \tag{S.1}$$

For simplicity, we will assume that electrons within the interfaces are in a steady-state condition such that $\partial n(x,t)/\partial t = 0$ and

$$-\frac{\partial j_n(x,t)}{\partial x} = r(x,t), \tag{S.2}$$

where:

$$j_n(x,t) = \mu_n(x,t)n\left(-\frac{\partial \Phi_{\mathrm{EA}}(x)}{\partial x} - qF\right) - D_n\left(\frac{\partial n(x,t)}{\partial x} - \frac{n(x,t)}{N_{\mathrm{CB}}(x)}\frac{\partial N_{\mathrm{CB}}(x)}{\partial x}\right). \tag{S.3}$$

$$j_n(x,t) = k_{\mathrm{B}}T\mu_n(x,t)\left(\alpha n(x,t) - \frac{\partial n(x,t)}{\partial x}\right) \tag{S.4}$$

where,

$$\alpha = -\frac{1}{k_{\mathrm{B}}T}\left(\frac{\partial \Phi_{\mathrm{EA}}(x)}{\partial x} + qF\right) + \frac{1}{N_{\mathrm{CB}}(x)}\frac{\partial N_{\mathrm{CB}}(x)}{\partial x}, \tag{S.5}$$

To solve Equation S.2 we make the following approximations and assumptions;

1. Transport within the interface is fast with respect to the surrounding materials such that the carriers within the interface can be treated as begin at steady-state.
2. Given that the interfacial regions are largely depleted of carriers, the electric field can be treated as constant throughout the interfaces.
3. The recombination rate r is uniform throughout the thickness of the interface such that it can be treated as constant for a given majority carrier boundary density.
4. The non-generalised Einstein relation ($D = k_{\mathrm{B}}T\mu$) can be applied (i.e. Boltzmann statistics are valid).

Under these conditions the following general solution to Equation S.2 can be obtained:

$$n(x') = \frac{C_I}{\alpha}\exp(\alpha x') - \frac{qrx'}{k_B T\alpha\mu_n} + C_{II}, \tag{S.6}$$

C_I, C_{II} are constants, and x' is the translated spatial co-ordinate such that $x' = x - x_1$, where x_1 is the position of the left-hand interface boundary as shown in Figure 4 of the Main Text.

Using the boundary conditions $n(0) = n_{\mathrm{s}}$, and flux $j_n(0) = j_{n,s}$ the following expressions for the electron density and flux within the interfaces can be obtained:

$$n(x') = n_{\mathrm{s}}e^{(\alpha x')} + \frac{j_{n,s}}{k_B T\alpha\mu_n}(1 - e^{(\alpha x')}) - \frac{r}{k_B T\alpha^2\mu_n}(1 - e^{(\alpha x')} + \alpha x'),$$

$$n(x') = n_s e^{\alpha x'} + \frac{j_{n,s}}{k_B T \alpha \mu_n}(1 - e^{\alpha x'}) - \frac{r}{k_B T \alpha^2 \mu_n}(1 - e^{\alpha x'} + \alpha x'), \quad \text{(S.7)}$$

$$j_n(x') = j_{n,s} - rx'. \quad \text{(S.8)}$$

A similar method can be used to obtain analogous expressions for the hole carrier and flux densities:

$$p(x') = p_s e^{(\beta x')} + \frac{j_{p,s}}{k_B T \beta \mu_p}(1 - e^{(\beta x')}) - \frac{r}{k_B T \beta^2 \mu_p}(1 - e^{(\beta x')} + \beta x'),$$

$$p(x') = p_s e^{\beta x'} + \frac{j_{p,s}}{k_B T \beta \mu_p}(1 - e^{\beta x'}) - \frac{r}{k_B T \beta^2 \mu_p}(1 - e^{\beta x'} + \beta x'), \quad \text{(S.9)}$$

$$j_p(x') = j_{p,s} - rx'. \quad \text{(S.10)}$$

where,

$$\beta = \frac{1}{k_B T}\left(\frac{\partial \Phi_{\mathrm{IP}}(x)}{\partial x} + qF\right) + \frac{1}{N_{\mathrm{VB}}(x)}\frac{\partial N_{\mathrm{VB}}(x)}{\partial x} \quad \text{(S.11)}$$

S.3.2 Validation under different transport and recombination regimes

Repository branch: `Methods-interface-solutions`
Parameters file: `./Input_files/3_layer_test_vary.csv`
Script: `./Scripts/interface_numerical_analytical`

To validate the analytical solutions derived in Section S.3.1, here we present results comparing analytical and numerical solutions of the carrier densities within the interfacial regions for a three-layer device including ionic carriers in the active layer. Solely for testing purposes a constant and uniform recombination rate, r_{con} was set throughout the interfacial regions. Four different mobility and recombination regimes were tested; 1. High mobility, no recombination; 2. Low mobility, no recombination; 3. High mobility, high recombination; and 4. Low mobility, high recombination. Current-voltage scans from 0 to 1 to 0 V at a scan rate of 100 mV s^{-1} were executed in each case. To calculate the analytical values the boundary values n_s, p_s, $j_{n,s}$ and $j_{p,s}$ were taken from the numerical solution (the nearest interior point on the subinterval mesh) for each interface. The solutions in Figure S.3 are plotted at $t = 0, 4, 8$ and 12 s, corresponding to $V_{app} = 0, 0.4, 0.8$ (forward scan), and 0.8 (reverse scan) V.

The results from the analytical and numerical solutions show close agreement. The small differences between the two can be accounted for by the fact that the fluxes are calculated on the subinterval mesh. In the low mobility, high recombination case, negative carrier densities result from the unphysical constant recombination rate. In practice this can be avoided by using relatively high mobilities within the interfacial regions such that the r term in Equations S.7 and S.9 is minimised. As discussed in Section 3.7.3, the use of a recombination zone further minimises this issue by including recombination only in the region with the highest minority carrier density.

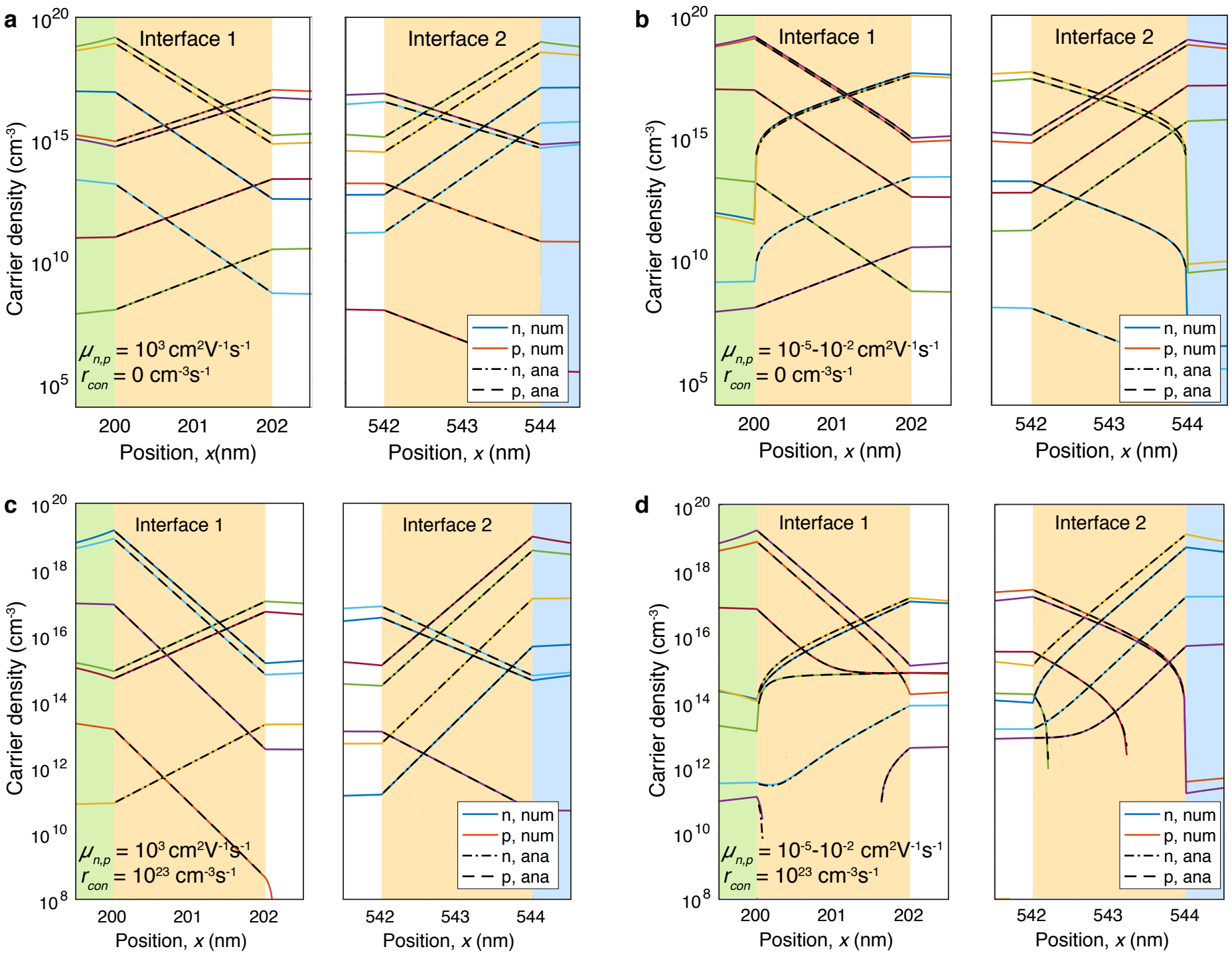

Fig. S.3 A comparison of numerical solutions and analytical approximations to the interfacial carrier density profiles within a three-layered solar cell with mobile ions during current-voltage scans. The following mobility and recombination regimes were tested; (**a**) High mobility, no recombination; (**b**) Low mobility, no recombination; (**c**) High mobility, high recombination and; (**d**) Low mobility, high recombination. Solutions are plotted at $t = 0, 4, 8$ and 12 s, corresponding to $V_{app} = 0, 0.4, 0.8$ (forward scan), and 0.8 (reverse scan) V. A constant and uniform recombination rate, r_{con} was set throughout the interfacial regions. Numerical (num) and analytical (ana) solutions for electrons (n) and holes (p) are indicated by solid colours and black dashed curves respectively.

S.4 Interfacial volumetric surface recombination model

S.4.1 Recombination zone

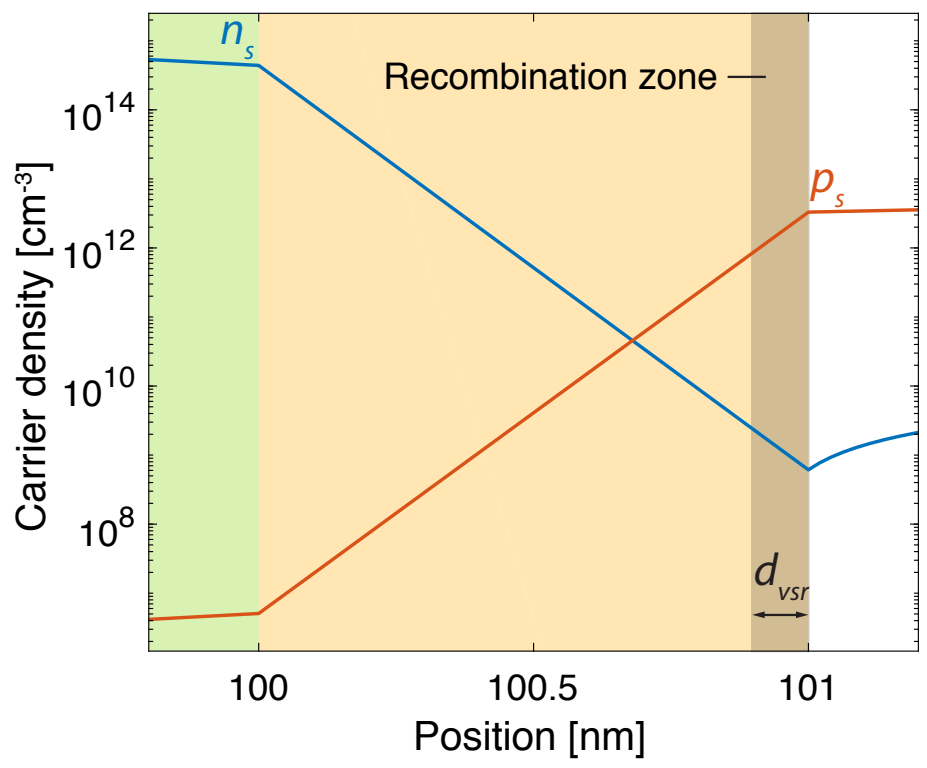

Fig. S.4 Recombination zone within an interface for the volumetric surface recombination scheme. The recombination zone for the volumetric surface recombination model is located close to the boundary with the highest minority carrier density.

S.5 System architecture

S.5.1 Creating the generation profile

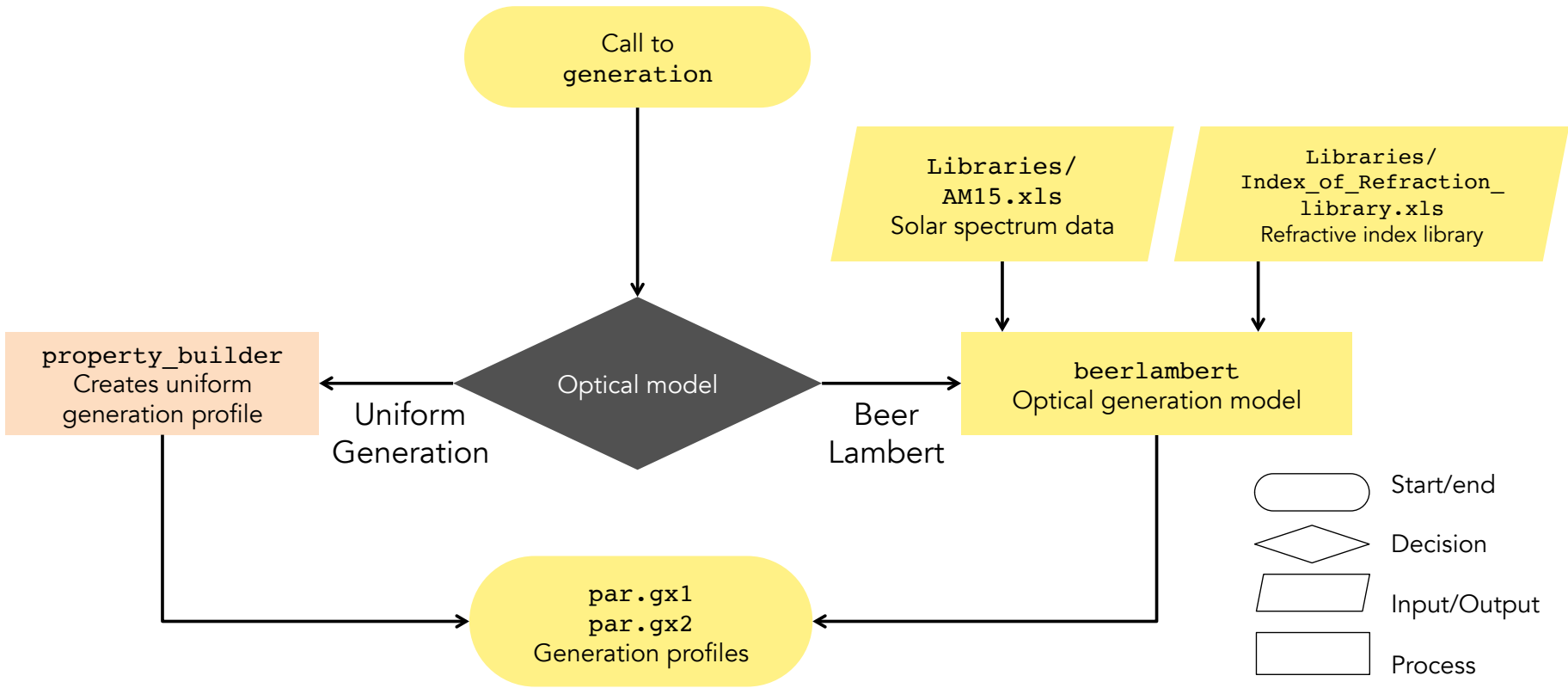

Fig. S.5 Flow diagram for building the generation profile.

S.6 Using the equation editor to adapt the physical model: Example

In the default version of Driftfusion ionic carriers do not have generation and recombination terms. In the following example we show how to change the underlying physical model by adding generation and recombination terms for ionic carriers. In general, the following steps are required:

1. Add terms to the equation editor `dfpde`, initial conditions `dfic` and boundary conditions `dfbc` subfunctions in `df` as required.
2. Ensure that changes in the equation editor and boundary conditions are reproduced comprehensively in `dfana`, with particular attention to `dfana.calcJ` (flux terms) and `dfana.calcr` (recombination terms).
3. Add necessary properties to `pc`.
4. Define how the properties are graded in `build_device`.

S.6.1 Switching to two ionic carrier species

We start with the default expressions shown in Listing 1 of the Main Text and the input properties defined in `Input_files/spiro_mapi_tio2.csv`. To ensure charge conservation we maintain ionic carrier neutrality, two ionic carrier variables are required. As such we first need to set `N_ionic_species` to 2 in the .csv file as shown in Figure S.6

sn_l	sn_r	sp_l	sp_r	PhiA	PhiC	OM	xmesh_type	side	N_ionic_species
1.00E+07	1.00E+07	1.00E+07	1.00E+07	-4.80E+00	-4.20E+00	1.00E+00	5.00E+00	2.00E+00	2

Fig. S.6 Setting the number of ionic carrier variables to 2.

S.6.2 Adding new device properties

In order to be able to adjust our generation term we will introduce two new device properties `k_iongen` and `k_ionrec` that characterise the ionic generation and recombination rates. For simplicity we will assume that these terms are material specific and equal to zero in the transport layers of our three-layer device. `k_iongen` and `k_ionrec` need to be added as arrays to the parameters class `pc` and the device builder before we can create a parameters object for the device. First we create the properties in `pc` with default values as shown in Listing S1.

Listing S2 shows how new properties can then be added to the device builder. The new coefficient arrays `k_iongen` and `k_ionrec` are used as the first input arguments. The device building code `build_device` then uses these values with our chosen interface grading option to define the values of these properties at every point in the device and stores the resulting arrays in the device structures `par.dev` and `par.dev_sub`. Here the `'lin_graded'` option is chosen to linearly grade the new properties in the interface regions.

Once the new properties have been added to the `pc` and `build_device`, they can additionally be added to the `.csv` parameters file and `import_properties` to enable them to be easily imported. Figure S.7 shows two new columns added to the `.csv` file defining `k_iongen` and `k_ionrec` for each layer.

```
144 %% Mobile ions
145 % Mobile ion defect density [cm-3]
146 N_ionic_species = 1;
147 K_anion = 1;          % Coefficients to easily accelerate ions
148 K_cation = 1;         % Coefficients to easily accelerate ions
149 Nani = [1e19];
150 Ncat = [1e19];
151 % Approximate density of iodide sites [cm-3]
152 % Limits the density of iodide vancancies
153 a_max = [1.21e22];
154 c_max = [1.21e22];
155 % Ionic generation and recombination rates
156 k_iongen = [0];
157 k_ionrec = [0];
```

Listing S. 1 Creating property arrays in the parameters class. New coefficients for ionic generation and recombination coefficients are added in lines 156 and 157.

```
function dev = build_device(par, meshoption)
% BUILD_DEVICE calls BUILD_PROPERTY for each device property. BUILD_PROPERTY
    then defines the
% properties at each point on the grid defined by MESHOPTION

switch meshoption
    case 'whole'
        xmesh = par.xx;
    case 'sub'
        xmesh = par.x_sub;
end

% Properties with constant values in the interfacial regions
% Constant properties
dev.mu_c = build_property(par.mu_c, xmesh, par, 'constant', 0);
dev.mu_a = build_property(par.mu_a, xmesh, par, 'constant', 0);
dev.epp = build_property(par.epp, xmesh, par, 'constant', 0);
dev.k_iongen = build_property(par.k_iongen, xmesh, par, 'constant', 0);
dev.k_ionrec = build_property(par.k_ionrec, xmesh, par, 'constant', 0);

% Linearly graded properties
dev.EA = build_property(par.EA, xmesh, par, 'lin_graded', 0);
dev.IP = build_property(par.IP, xmesh, par, 'lin_graded', 0);
dev.E0 = build_property(par.E0, xmesh, par, 'lin_graded', 0);

% Logarithmically graded properties
dev.NA = build_property(par.NA, xmesh, par, 'zeroed', 0);
dev.ND = build_property(par.ND, xmesh, par, 'zeroed', 0);
dev.Nc = build_property(par.Nc, xmesh, par, 'log_graded', 0);
```

Listing S. 2 Adding properties to the device builder.

c_max	a_max	mu_n	mu_p	mu_c	mu_a	epp	g0	k_iongen	k_ionrec	B
1.21E+22	1.21E+22	1	1	0.00E+00	0.00E+00	4.00E+00	0.00E+00	0.00E+00	0.00E+00	3.18E-11
1.21E+22	1.21E+22	1.00E+05	1.00E+05	0.00E+00	0.00E+00	2.30E+01	0.00E+00	0.00E+00	0.00E+00	0.00E+00
1.21E+22	1.21E+22	2.00E+01	2.00E+01	1.00E-10	1.00E-10	2.30E+01	2.64E+21	1.00E+03	1.00E+00	3.60E-12
1.21E+22	1.21E+22	1.00E+05	1.00E+05	0.00E+00	0.00E+00	2.30E+01	0.00E+00	0.00E+00	0.00E+00	0.00E+00
1.21E+22	1.21E+22	1.00E+01	1.00E+01	0.00E+00	0.00E+00	1.20E+01	0.00E+00	0.00E+00	0.00E+00	1.54E-10

Fig. S.7 Adding new properties to the .csv file. The new properties `k_iongen` and `k_ionrec` have been added to the final two columns.

```
try
    par.k_iongen = T{1, 'k_iongen'};
catch
    warning('k_iongen undefined in .csv. Using default in PC')
end
try
    par.k_ionrec = T{1, 'k_ionrec'};
catch
    warning('k_ionrec undefined in .csv. Using default in PC')
end
```

Listing S. 3 Including new properties to be imported from the .csv file in import_properties.

New properties can be inserted anywhere the user chooses into the table as `import_properties` checks for matches between the column headers and listed property names. Lastly, the new properties are added to `import_properties` using try statements (Listing 3). If a match to a column heading is found in the `.csv` file the the properties are imported, otherwise the default values in `pc` are used.

This completes the steps necessary to add new properties to the parameters object and make them easily accessible from the .csv file. We will now look at how to adapt the physical model using the Equation Editor to include our new ionic carrier generation and recombination terms.

S.6.3 Using the equation editor

For simplicity, we will assume that ionic carriers are generated at a rate proportional to the optical intensity and hence the electron and hole generation rate g. We will further assume that the ionic carriers recombine at a rate proportional to their respective densities. *We stress here that there is no physical basis for this choice of models and they are only been used for illustrative purposes.* To adapt the Driftfusion master code `df` to our purposes we first need to unpack the new variables from the parameters object by adding the to the list of variables at the start of the code as shown in Listing S4.

While this approach is laborious, creating individual variables in the workspace in this way (as opposed to using `par.k_iongen` directly in the Equation Editor) dramatically improves the performance of the code.

```
epp = device.epp;              % Dielectric constant
eppmax = max(par.epp);         % Maximum dielectric constant (for normalisation)
B = device.B;                  % Radiative recombination rate coefficient
ni = device.ni;                % Intrinsic carrier density
taun = device.taun;            % Electron SRH time constant
taup = device.taup;            % Electron SRH time constant
taun_vsr = device.taun_vsr; % Electron SRH time constant- volumetric
    interfacial surface recombination scheme
taup_vsr = device.taup_vsr; % Electron SRH time constant- volumetric
    interfacial surface recombination scheme
k_iongen = device.k_iongen; % Ion generation coefficient
k_ionrec = device.k_ionrec; % Ion recombination coefficient
```

Listing S. 4 Unpacking the new coefficients at the start of df.

```
247 %% Equation editor
248 % Time-dependence prefactor term
249 C_V = 0; C_n = 1; C_p = 1; C_c = 1; C_a = 1;
250 C = [C_V; C_n; C_p; C_c; C_a];
251
252 % Flux terms
253 F_V = (epp(i)/eppmax)*dVdx;
254 F_n = mu_n(i)*n*(-dVdx + gradEA(i)) + (Dn(i)*(dndx - ((n/Nc(i))*gradNc(i))));
255 F_p = mu_p(i)*p*(dVdx - gradIP(i)) + (Dp(i)*(dpdx - ((p/Nv(i))*gradNv(i))));
256 F_c = mu_c(i)*(z_c*c*dVdx + kB*T*(dcdx + (c*(dcdx/(c_max(i) - c)))));
257 F_a = mu_a(i)*(z_a*a*dVdx + kB*T*(dadx + (a*(dadx/(a_max(i) - a)))));
258 F = [F_V; mobset*F_n; mobset*F_p; mobseti*K_c*F_c; mobseti*K_a*F_a];
259
260 % Electron and hole recombination
261 % Radiative
262 r_rad = radset*B(i)*(n*p - ni(i)^2);
263 % Bulk SRH
264 r_srh = SRHset*srh_zone(i)*((n*p - ni(i)^2)/(taun(i)*(p + pt(i)) + taup(i)*(n + nt(i))));
265 % Volumetric surface recombination
266 alpha = sign_xn(i)*q*dVdx/(kB*T) + alpha0_xn(i);
267 beta = sign_xp(i)*q*-dVdx/(kB*T) + beta0_xp(i);
268 r_vsr = SRHset*vsr_zone(i)*((n*exp(-alpha*xprime_n(i))*p*exp(-beta*xprime_p(i)) - ni(i)^2)...
269     /(taun_vsr(i)*(p*exp(-beta*xprime_p(i)) + pt(i)) + taup_vsr(i)*(n*exp(-alpha*xprime_n(i)) + nt(i))));
270 % Total electron and hole recombination
271 r_np = r_rad + r_srh + r_vsr;
272
273 % Source terms
274 S_V = (q/(eppmax*epp0))*(-n + p - NA(i) + ND(i) + z_a*a + z_c*c - z_a*Nani(i) - z_c*Ncat(i));
275 S_n = g - r_np;
276 S_p = g - r_np;
277 S_c = k_iongen*g - k_ionrec*(c*a-Ncat(i)*Nani(i));
278 S_a = 0;
279 S = [S_V; S_n; S_p; S_c; S_a];
```

Listing S. 5 Adding source terms for mobile cations in the Equation Editor. New terms for generation and recombination of cations are introduced on line 277.

These terms are then simply added to the appropriate lines in the Equation Editor as shown in Listing S5.

S.6.4 Adapting analysis functions in `dfana` to the new physical model

It is particularly important to bear in mind that the physical models in the master code and the analysis functions are not coupled. Users therefore need to adapt any of the analysis functions in `dfana` to account for changes to the physical model. For example, if one were to change the recombination model, `dfana.calcr` would need to be updated in accordance with the changes made to the expressions in the Equation Editor.

S.7 Troubleshooting

We hope that your experience using Driftfusion will be a relatively trouble-free experience. Along with the possibility of minor bugs that come with experimental research software,

you may encounter two common error messages: spatial discretization and time integration failures.

S.7.1 Spatial discretization has failed

This error typically occurs for one of two reasons:

1. The point density of spatial mesh is too high: Try reducing the number of points in individual layers or using a different spatial mesh type (see `meshgen_x` for possible options).
2. The boundary conditions are not consistent with initial conditions: The initial value of a variable must be consistent with those set in the boundary conditions. If the initial or boundary conditions are changed from the default, ensure that they are consistent with one another.

S.7.2 Time integration failure

This error occurs when the solver cannot converge and has a number of possible solutions:

1. Reduce the `par.tmax` property to reduce the total time of the simulation.
2. Reduce the `par.MaxStepFactor` property to reduce the solver's maximum time step. Please note that this may slow down solving time considerably.
3. Use the `par.K_cation` and `par.K_anion` properties to change the ionic carrier transport to be on a similar time scale to electronic carrier transport.
4. Split the protocol into a number of intermediate steps and associated solutions.
5. Relax (increase) the solver tolerances `par.RelTol` and `par.AbsTol`.
6. Stabilise the solution with the ionic carriers frozen (set `par.mobseti = 0`) then run a subsequent simulation with the ion transport switched on (set `par.mobseti = 1`).

Time integration errors can also occur when inconsistencies are introduced to the physical model in the simulation.

S.7.3 Unexpected values calculated using `dfana`

As discussed in the Main Text, owing to the computational cost of using functions external to `df` for the solving the equations, *the physical model described in the Equation Editor is not coupled to that used in the analysis functions*. For greater consistency an obvious change to the programming architecture would be to use centralised flux and source functions to describe the physical model, which could then be called during both the solving and analysis stages. Multiple tests have shown, however, that this method comes with a significant increase in computation cost owing to the large number of calls made by the solver to such functions. Hence the present method of adapting the model in both `df` and `dfana` separately, while more prone to user error, is considerably more efficient.

S.7.4 Bug reporting

If you find a bug with Driftfusion please raise an issue using the `Issues` tab on the Driftfusion GitHub repository page.[1] This is the best way to ensure that other users can see which bugs have been addressed and understand how they have been fixed.

S.8 Known issues

S.8.1 Linear discretisation

Many existing drift-diffusion models use Scharfetter-Gummel finite volume discretisation scheme[2][3] to account for the exponential change in carrier density in a constant electric field. In order to take advantage of MATLAB's Partial Differential Equation Parabolic and Elliptic (PDEPE) toolbox and the ease with which transport models can be altered with it, a major trade off is the use a simplified finite element discretisation scheme for which carrier densities are assumed to change linearly between neighbouring grid points. This can be compensated for somewhat with the use of a logarithmically-spaced spatial grid and a higher density of grid points. There are however limits to the allowed point density and while the comparison simulations that use the Scharfetter-Gummel scheme (Section 5.3 of the Main Text) is generally good, the user should be aware that the use of lower point densities will increase this error. One consequence of this simplified scheme is that the currents may not always be close to zero at equilibrium. Following exploratory work at lower point densities, users are encouraged to use as high a point density as possible for obtaining the final results, especially since the solver is highly efficient. Communication with Mathworks has suggested the maximum point density is a function of various factors and therefore cannot be distilled to a single value. A degree of trial and error is therefore required on the part of the user to find the maximum allowed point density for a given device configuration.

S.8.2 Numerical errors with interfacial currents

Following from the brief discussion in Subsection S.8.1, and as noted in the comparison of Driftfusion and ASA results in Section 5.3, the linear discretisation method and large gradients in the band energies and eDOS within the interfacial interlayers of Driftfusion makes these regions particularly prone to numerical errors. As shown in Section S.9.4, errors can be minimised by reducing band energy and eDOS gradients as well as increasing the number of spatial mesh points within the interfaces. However, where accurate calculation of very low current densities ($< 10^{-12}$ A cm^{-2} for example, based on the ASA comparison calculations) across one or more heterojunction interfaces is required we presently do not recommend the use of Driftfusion for this purpose.

S.8.3 Normalisation of the dielectric constant

As may be noted from the Equation Editor (Listing 1, Main Text) the dielectric constant must be normalised for the electrostatic potential flux and source terms to avoid a spatial discretisation error. The origin of this problem is currently unknown as normalisation has little effect on the magnitude of the input values.

S.8.4 High extraction coefficients do not result in constant charge density at the boundaries

Under some uncommon operating conditions e.g. large preconditioning voltages, the carrier densities at the boundaries do not tend to their limiting value when using high extraction

coefficients. Under most circumstances this can be solved by switching to fixed carrier densities at the boundaries. This does however present a problem when calculating currents for a single carrier device as the minority carrier fluxes can no longer be used as the boundary value for the fluxes. This issue is currently ongoing and under investigation.

S.8.5 Drift and diffusion currents do not sum to give the total current

Drift and diffusion currents calculated using `dfana.Jddxt` do not sum to give the correct current. This is related to the way in which fluxes are calculated in the solver but a solution has yet to present itself. Total carrier currents are correctly calculated using the continuity equations in `dfana.calcJ` and users are recommended to use this method instead, reserving `dfana.Jddxt` only for individual analysis of the approximate drift and diffusion currents.

S.8.6 `lightOnRs` protocol instability

At the time of writing `lightOnRs` protocol does not converge for all devices parameter sets at high values of series resistance.

S.9 Validation against existing models

The following sections provide references to the relevant Github Driftfusion repository branches, parameters files and scripts required to reproduce the results in Section 5 of the Main Text.

S.9.1 The depletion approximation for a p-n junction

Repository branch:	`Methods-depletion-approximation-comparison`
Parameters file:	`./Input_files/TPV_test.csv`
Script:	`df_methods_depletion_approx_pn_junction.m`

Table S.5 Key layer-specific parameters in the Driftfusion simulation comparison with the depletion approximation for a p-n junction.

Variable	Symbol	p-type quasi-neutral	p-type depletion region	n-type depletion region	n-type quasi-neutral	Unit
Thickness	d	10^{-2}	1.38×10^{-5}	6.25×10^{-5}	10^{-2}	cm
Electron affinity	Φ_{EA}	0	0	0	0	eV
Ionisation potential	Φ_{IP}	-1.12	-1.12	-1.12	-1.12	eV
Equilibrium Fermi energy	E_{F0}	-0.94	-0.94	-0.22	-0.22	eV
Acceptor density	N_{A}	2.01×10^{15}	2.01×10^{15}	0	0	cm^{-3}
Donor density	N_{D}	0	0	9.47×10^{15}	9.47×10^{15}	cm^{-3}
SRH trap energy	E_{trap}	-0.56	-0.56	-0.56	-0.56	eV
eDOS conduction band	N_{CB}	10^{19}	10^{19}	10^{19}	10^{19}	cm^{-3}
eDOS valence band	N_{VB}	10^{19}	10^{19}	10^{19}	10^{19}	cm^{-3}
Electron mobility	μ_e	2000	2000	2000	2000	$\mathrm{cm}^2\ \mathrm{V}^{-1}\ \mathrm{s}^{-1}$
Hole mobility	μ_h	2000	2000	2000	2000	$\mathrm{cm}^2\ \mathrm{V}^{-1}\ \mathrm{s}^{-1}$
Relative dielectric constant	ε_r	12	12	12	12	-
Uniform generation rate	g_0	0	3.49×10^{21}	3.49×10^{21}	0	$\mathrm{cm}^{-3}\ \mathrm{s}^{-1}$
First order recombination electron lifetime	τ_n	$10^{-6}, 10^{-7}, 10^{-8}$	10^{100}	10^{100}	10^{100}	s
First order recombination hole lifetime	τ_p	10^{100}	10^{100}	10^{100}	$10^{-6}, 10^{-7}, 10^{-8}$	s

Table S.6 Key device-wide parameters in the Driftfusion simulation comparison with the depletion approximation for a p-n junction.

Variable	Symbol	Unit	
Left-hand boundary Fermi energy	Φ_l	-0.94	eV
Right-hand boundary Fermi energy	Φ_r	-0.22	eV
Left-hand boundary electron extraction coefficient	$s_{n,l}$	0	$\mathrm{cm\ s}^{-1}$
Right-hand boundary electron extraction coefficient	$s_{n,r}$	10^{10}	$\mathrm{cm\ s}^{-1}$
Left-hand boundary hole extraction coefficient	$s_{p,left}$	10^{10}	$\mathrm{cm\ s}^{-1}$
Right-hand boundary hole extraction coefficient	$s_{p,r}$	0	$\mathrm{cm\ s}^{-1}$

S.9.1.1 Additional data

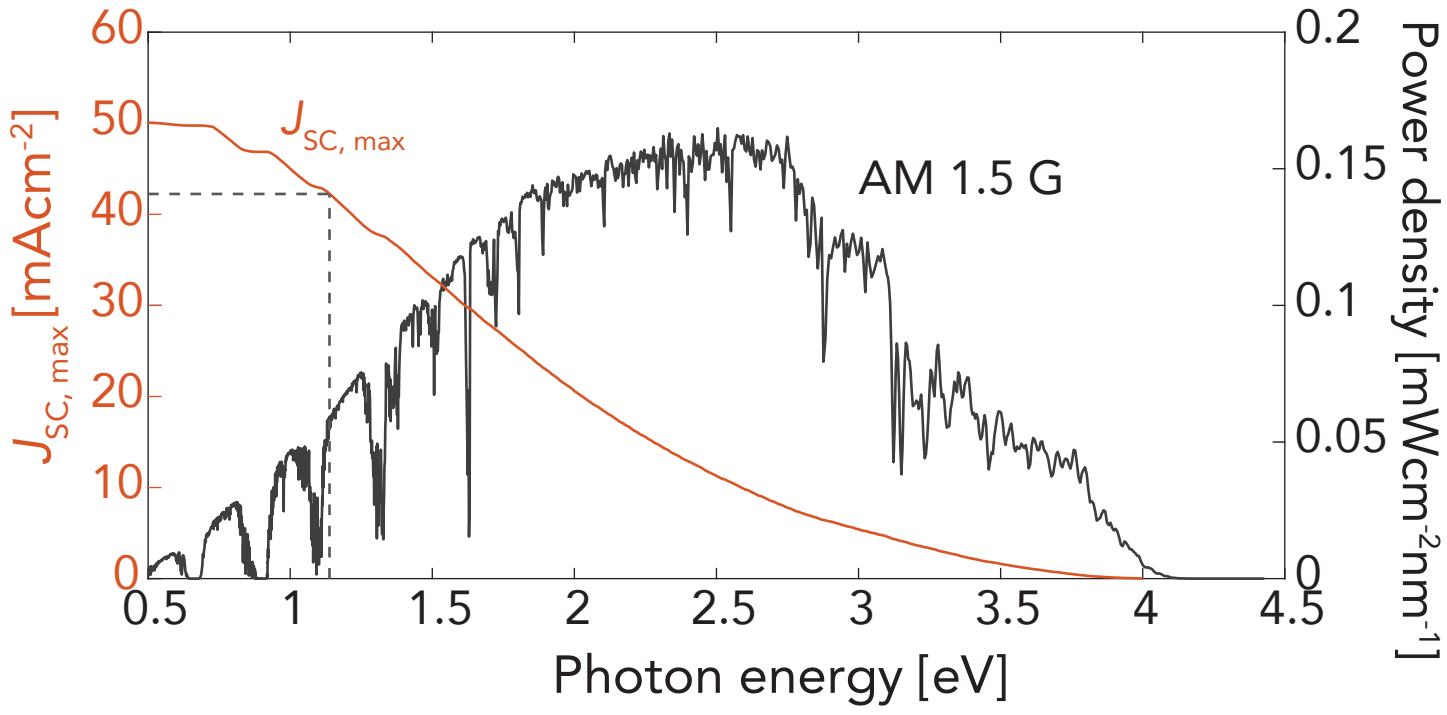

Fig. S.8 AM 1.5 solar spectrum and maximum theoretical short circuit current. AM 1.5 power density versus wavelength (right axis, black curve). Integrating the spectral photon flux above the band gap gives the maximum theoretical short circuit current $J_{SC,max}$ (left axis, red curve). The dashed line indicates the position of 1.12 eV. Note: 'Global Tilt' is for a south facing surface at an angle of 37 degrees to the horizontal and includes sky diffuse and diffuse reflected light from the ground.[4]

S.9.2 Transient photovoltage response of a single field-free device

Repository branch:	`Methods`
Parameters file:	`./Input_files/TPV_test.csv`
Script:	`df_methods_TPV.m`

Table S.7 Key layer-specific parameters in the Driftfusion simulation comparison with transient photovoltage kinetic model analytical solution.

Variable	Symbol	Absorber layer	Unit
Thickness	d	10^{-5}	cm
Electron affinity	Φ_{EA}	-3.8	eV
Ionisation potential	Φ_{IP}	-5.4	eV
Equilibrium Fermi energy	E_{F0}	-4.6	eV
Acceptor density	N_A	0	cm^{-3}
Donor density	N_D	0	cm^{-3}
eDOS conduction band	N_{CB}	10^{20}	cm^{-3}
eDOS valence band	N_{VB}	10^{20}	cm^{-3}
Electron mobility	μ_e	10	cm^2 V^{-1} s^{-1}
Hole mobility	μ_h	10	cm^2 V^{-1} s^{-1}
Relative dielectric constant	ε_r	23	-
Uniform generation rate	g_0	1.89×10^{21}	cm^{-3} s^{-1}
Direct recombination coefficient	k_{rad}	10^{-10}	s

Table S.8 Key device-wide parameters in the Driftfusion simulation comparison with transient photovoltage kinetic model analytical solution.

Variable	Symbol	Unit	
Left-hand boundary Fermi energy	Φ_l	−4.6	eV
Right-hand boundary Fermi energy	Φ_r	−4.6	eV
Left-hand boundary electron extraction coefficient	$s_{n,l}$	0	cm s^{-1}
Right-hand boundary electron extraction coefficient	$s_{n,r}$	0	cm s^{-1}
Left-hand boundary hole extraction coefficient	$s_{p,l}$	0	cm s^{-1}
Right-hand boundary hole extraction coefficient	$s_{p,r}$	0	cm s^{-1}

S.9.2.1 Derivation of a zero-dimensional kinetic model of recombination

This section has been largely reproduced from reference [5].

Under the assumption that the parabolic band and Boltzmann approximations are valid,[2] the charge densities of electrons n and holes p for an intrinsic semiconductor with intrinsic carrier density n_i and Fermi energy E_Fi are given by:[6]

$$n = n_\mathrm{i} \exp\left(\frac{E_\mathrm{Fn} - E_\mathrm{Fi}}{k_\mathrm{B}T}\right) \tag{S.12}$$

$$p = n_\mathrm{i} \exp\left(\frac{E_\mathrm{Fi} - E_\mathrm{Fp}}{k_\mathrm{B}T}\right) \tag{S.13}$$

Using Equations S.12 and S.13, the open circuit (OC) voltage V_OC in a field free, zero-dimensional material, where electron and hole charge carrier concentrations are equal ($n = p$), can be expressed as:

$$V_\mathrm{OC} = \frac{2k_\mathrm{B}T}{q} \ln\left(\frac{n_\mathrm{OC}}{n_\mathrm{i}}\right) \tag{S.14}$$

Here, n_OC is carrier density at OC and is dependent on the generation rate g and the recombination model. For example, in an idealised device with band-to-band recombination only, the recombination rate r is given by $r = B(n^2 - n_\mathrm{i}^2)$, where B is the band-to-band recombination coefficient. At OC steady-state, generation is equal to recombination ($r = g$) leading to:

$$n_\mathrm{OC} = \left(\frac{g}{B} + n_\mathrm{i}^2\right)^{1/2} \approx \left(\frac{g}{B}\right)^{1/2} \tag{S.15}$$

During a small perturbation measurement a small additional charge density $\Delta n << n_\mathrm{OC}$ is injected into the device such that the state of the system is not significantly altered. In a transient photovoltage measurement this charge is generated by an excitation light pulse imposed onto a background bias light (Figure S.9a).

Figure S.9b is a schematic showing how the relaxation of the excess electron carrier density after a pulse results in a change in the electron quasi Fermi level (QFL). The recombination of excess charge with rate constant k_{rec} , results in an associated decay of the QFLs. In this 0-D representation, the open circuit voltage is defined by the difference in electron and hole QFLs ($qV_\mathrm{OC} = E_\mathrm{Fn} - E_\mathrm{Fp}$) and decays with the same rate constant as the charge.

[2] Criteria for validity: the band edges can be modelled as parabolic functions and the quasi Fermi levels should be $> 3k_\mathrm{B}T$ from their respective bands.[6]

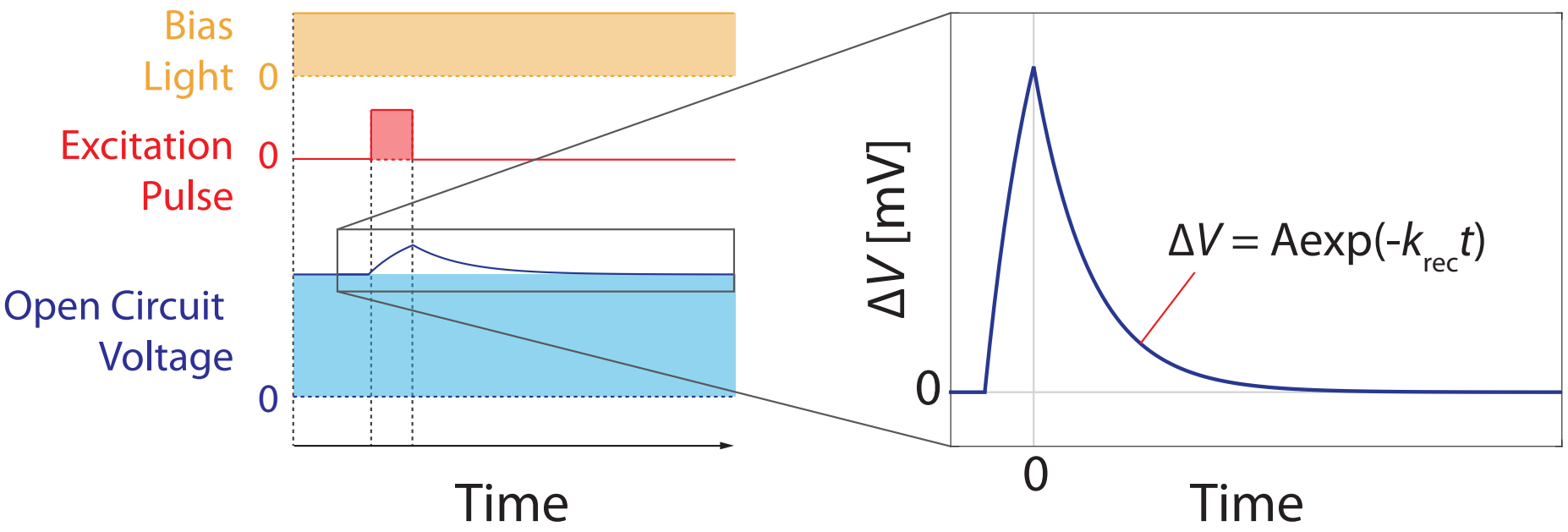

Fig. S.9 Transient photovoltage experimental timeline The exponential decay rate constant of ΔV equates to k_{rec} in the 0-D model.

In the most general case, following a small perturbation ending at $t = 0$, the rate of change of addition charge $\frac{\mathrm{d}\Delta n}{\mathrm{d}t}$ can be expressed using a small perturbation rate constant, k_{TPV} (see reference [7] , pp. 101 − 103 for full derivation):

$$\frac{\mathrm{d}\Delta n}{\mathrm{d}t} \approx -k_{\mathrm{TPV}}\Delta n \tag{S.16}$$

$$k_{\mathrm{TPV}} = \sum_{ij}\left(k_{ij}n^{\gamma_i}p^{\gamma_j}\left(\frac{\gamma_i}{n}+\frac{\gamma_j}{p}\right)\right) \tag{S.17}$$

where i and j are summation indices and the exponents γ_i and γ_j can take any value (including non-integers). k_{ij} is the associated rate coefficient for the reaction order. The solution to Equation S.16 is an exponential:

$$\Delta n = \exp(-k_{\mathrm{TPV}}t) \qquad \text{for } t > 0 \tag{S.18}$$

Using Equation S.14, the change in open circuit voltage ΔV_{OC} produced by a light pulse introducing an additional charge Δn can be expressed as:

$$\Delta V_{\mathrm{OC}} = \frac{2k_{\mathrm{B}}T}{q}\ln\left(1+\frac{\Delta n}{n_{\mathrm{OC}}}\right) \approx \frac{2k_{\mathrm{B}}T\Delta n}{n_{\mathrm{OC}}}$$

Substituting for Δn using Equation S.18 leads to:

$$\Delta V_{\mathrm{OC}} = \frac{2k_{\mathrm{B}}T}{qn_{\mathrm{OC}}}\exp(-k_{\mathrm{TPV}}t) \qquad \text{for } t > 0 \tag{S.19}$$

A similar method can be used to find the change in charge carrier density and voltage rise during a pulse of duration t_{pulse}:

$$\Delta n = \frac{\Delta g}{k_{\mathrm{TPV}}}\left(1-\exp\left(-k_{\mathrm{TPV}}(t+t_{\mathrm{pulse}})\right)\right) \qquad \text{for } -t_{\mathrm{pulse}} < t <= 0$$

$$\Delta n = \frac{\Delta g}{k_{\mathrm{TPV}}}\left(1-\exp\left(-k_{\mathrm{TPV}}(t+t_{\mathrm{pulse}})\right)\right) \qquad \text{for } -t_{\mathrm{pulse}} < t \leqslant 0 \tag{S.20}$$

$$\Delta V = \frac{2k_{\mathrm{B}}T}{qn_{\mathrm{OC}}}\frac{\Delta g}{k_{\mathrm{TPV}}}\left(1-\exp\left(-k_{\mathrm{TPV}}(t+t_{\mathrm{pulse}})\right)\right) \qquad \text{for } -t_{\mathrm{pulse}} < t <= 0$$

$$\Delta V = \frac{2k_B T}{q n_{OC}} \frac{\Delta g}{k_{TPV}} \left(1 - \exp\left(-k_{TPV}(t + t_{pulse})\right)\right) \quad \text{for } -t_{pulse} < t \leqslant 0 \tag{S.21}$$

where Δg is the additional generation rate from the pulse light.

In experimental measurements, a single empirical reaction order γ, with the corresponding rate constant k_γ, is typically assumed to dominate recombination such that:

$$r = k_\gamma n^\gamma \tag{S.22}$$

Accordingly, the slope of the $\log(k_{TPV})$ vs. $\log(n_{OC})$ plot can be used to determine γ:

$$\log(k_{TPV}) = (\gamma - 1)\log(n_{OC}) + \log(\gamma k_\gamma) \tag{S.23}$$

S.9.3 Numerical solution for a three-layer device without mobile ions: comparison with Advanced Semiconductor Analysis (ASA) software

Repository branch:	`Methods`
Parameters files:	`./Input_files/3_layer_methods_test1a.csv`
	`./Input_files/3_layer_methods_test1b.csv`
	`./Input_files/3_layer_methods_test2a.csv`
	`./Input_files/3_layer_methods_test2b.csv`
Script:	`df_methods_ASA_comparison.m`

S.9.3.1 Parameter Sets 1a & 1b

Table S.9 Key layer-specific parameters for Parameter Sets 1a and 1b in the Driftfusion simulation comparison with ASA. HTL and ETL denote hole and electron transport layers respectively.

Variable	Symbol	Layer			Unit
		HTL	Absorber	ETL	
Optical parameters material reference	-	SiO_2	MAPICl	SiO_2	-
Thickness	d	2×10^{-5}	**(a)** 4×10^{-5} **(b)** 2×10^{-5}	0.5×10^{-5}	cm
Electron affinity	Φ_{EA}	-3.3	-3.8	-4.0	eV
Ionisation potential	Φ_{IP}	-5.3	-5.4	-6.2	eV
Equilibrium Fermi energy	E_{F0}	-5.2	-4.6	-4.15	eV
Acceptor density	N_A	2.09×10^{17}	0	0	cm^{-3}
Donor density	N_D	0	0	3.02×10^{16}	cm^{-3}
SRH trap energy	E_{trap}	-4.3	-4.6	-5.1	eV
eDOS conduction band	N_{CB}	10^{19}	10^{18}	10^{19}	cm^{-3}
eDOS valence band	N_{VB}	10^{19}	10^{18}	10^{19}	cm^{-3}
Electron mobility	μ_e	0.2	20	0.1	$cm^2\ V^{-1}\ s^{-1}$
Hole mobility	μ_h	0.02	20	0.01	$cm^2\ V^{-1}\ s^{-1}$
Relative dielectric constant	ε_r	4	23	12	-
SRH recombination electron lifetime	τ_n	10^{-8}	10^{-7}	10^{-9}	s
SRH recombination hole lifetime	τ_p	10^{-8}	10^{-7}	10^{-9}	s

Table S.10 Key device-wide parameters for Parameter Sets 1a and 1b in the Driftfusion simulation comparison with ASA.

Variable	Symbol	Unit	
Left-hand boundary Fermi energy	Φ_l	-5.2	eV
Right-hand boundary Fermi energy	Φ_r	-4.15	eV
Left-hand boundary electron extraction coefficient	$s_{n,l}$	10^8	cm s^{-1}
Right-hand boundary electron extraction coefficient	$s_{n,r}$	10^8	cm s^{-1}
Left-hand boundary hole extraction coefficient	$s_{p,l}$	10^8	cm s^{-1}
Right-hand boundary hole extraction coefficient	$s_{p,r}$	10^8	cm s^{-1}

S.9.3.2 Parameter Sets 2a & 2b

Table S.11 Key layer-specific parameters for Parameter Sets 2a and 2b in the Driftfusion simulation comparison with ASA. HTL and ETL denote hole and electron transport layers respectively.

Variable	Symbol		Layer		Unit
		HTL	Absorber	ETL	
Optical parameters material reference	-	SiO_2	MAPICl	SiO_2	-
Thickness	d	1×10^{-5}	2×10^{-5}	0.7×10^{-5}	cm
Electron affinity	Φ_{EA}	-3.0	-3.8	-4.1	eV
Ionisation potential	Φ_{IP}	-5.1	-5.2	-6.2	eV
Equilibrium Fermi energy	E_{F0}	-5.0	-4.6	-4.4	eV
Acceptor density	N_A	2.09×10^{17}	8.32×10^{7}	0	cm^{-3}
Donor density	N_D	0	0	9.12×10^{14}	cm^{-3}
SRH trap energy	E_{trap}	-4.05	-4.5	-5.15	eV
eDOS conduction band	N_{CB}	(**a**) 10^{19} (**b**) 10^{18}	10^{18}	(**a**) 10^{20} (**b**) 10^{18}	cm^{-3}
eDOS valence band	N_{VB}	(**a**) 10^{19} (**b**) 10^{18}	10^{18}	(**a**) 10^{20} (**b**) 10^{18}	cm^{-3}
Electron mobility	μ_e	0.2	20	0.1	$cm^2\ V^{-1}\ s^{-1}$
Hole mobility	μ_h	0.02	2	0.001	$cm^2\ V^{-1}\ s^{-1}$
Relative dielectric constant	ε_r	8	10	20	-
SRH recombination electron lifetime	τ_n	10^{-9}	10^{-7}	10^{-6}	s
SRH recombination hole lifetime	τ_p	10^{-9}	10^{-7}	10^{-6}	s

Table S.12 Key device-wide parameters for Parameter Sets 2a and 2b in the Driftfusion simulation comparison with ASA.

Variable	Symbol	Unit	
Left-hand boundary Fermi energy	Φ_l	-5.0	eV
Right-hand boundary Fermi energy	Φ_r	-4.4	eV
Left-hand boundary electron extraction coefficient	$s_{n,l}$	10^8	cm s^{-1}
Right-hand boundary electron extraction coefficient	$s_{n,r}$	10^8	cm s^{-1}
Left-hand boundary hole extraction coefficient	$s_{p,l}$	10^8	cm s^{-1}
Right-hand boundary hole extraction coefficient	$s_{p,r}$	10^8	cm s^{-1}

S.9.3.3 Beer Lambert generation profile

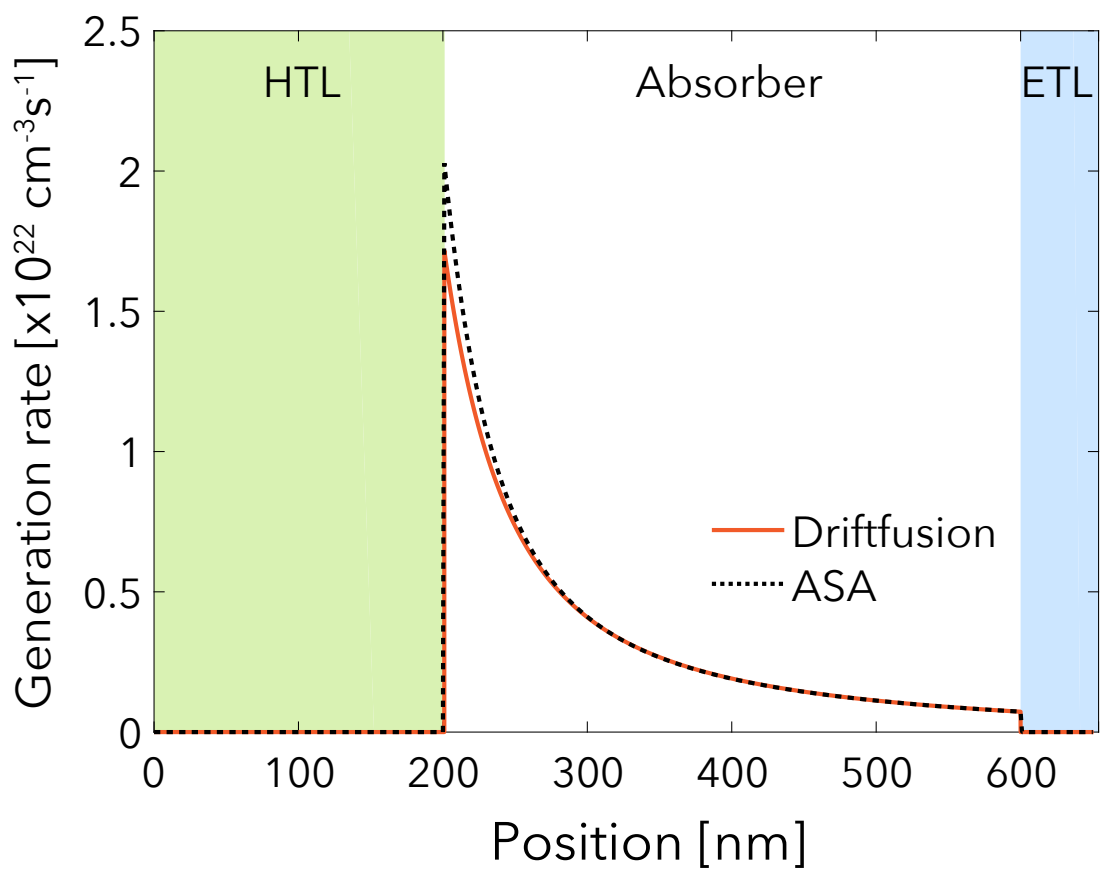

Fig. S.10 Integrated generation profile obtained using Driftfusion and ASA Beer Lambert models.

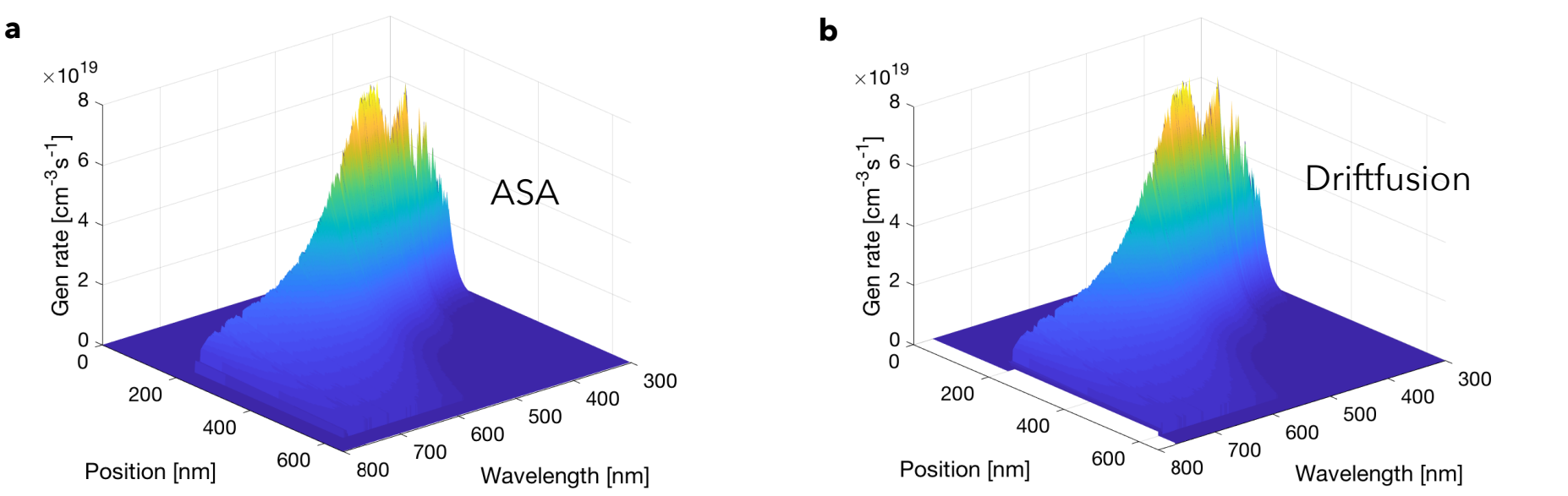

Fig. S.11 Comparison of optical generation rate as a function of wavelength and position using the Beer-Lambert model calculated by Driftfusion and ASA.

S.9.3.4 Equilibrium states

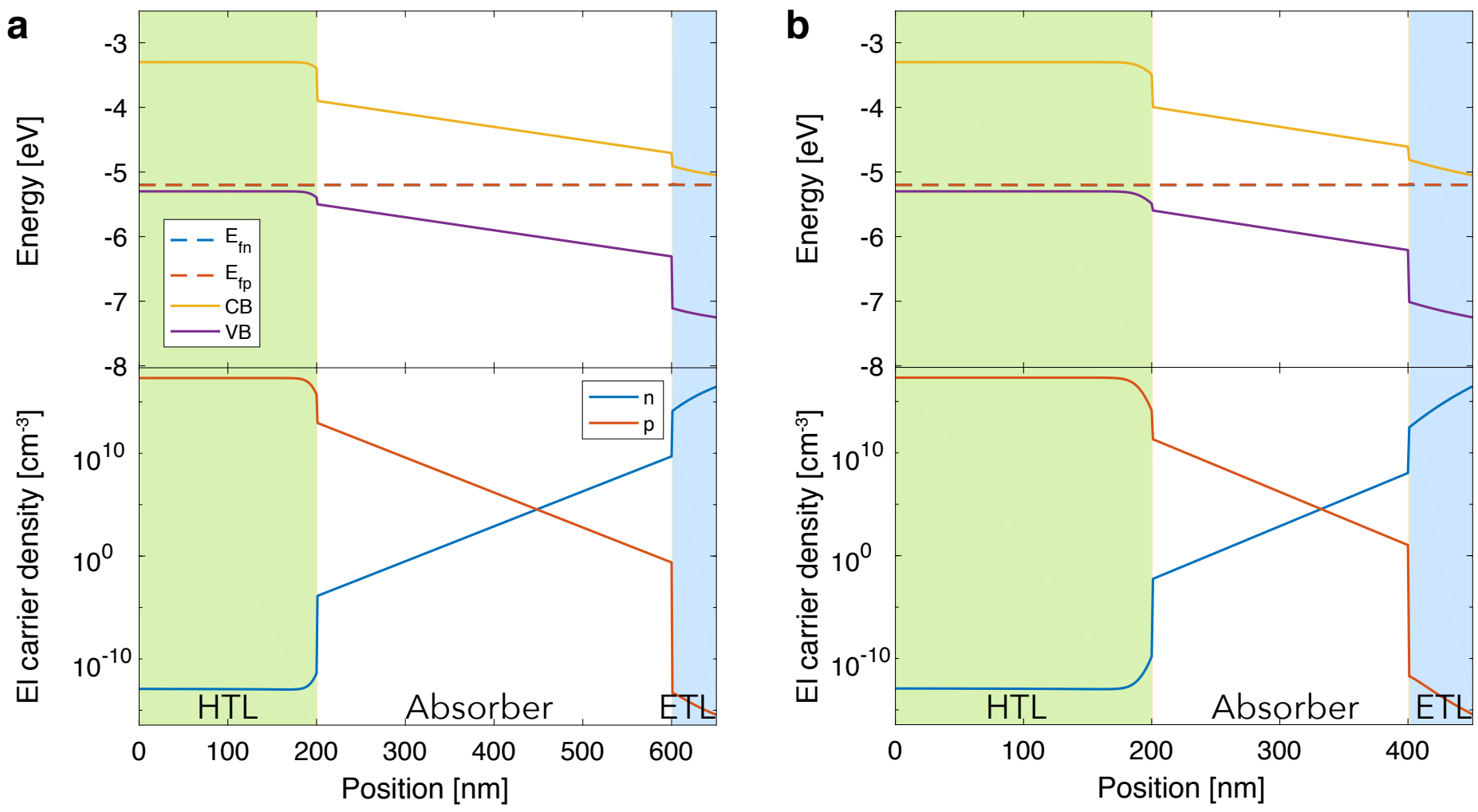

Fig. S.12 Equilibrium energy level diagrams and carrier densities for Parameter Sets 1a and 1b. Equilibrium states for (**a**) Parameter Set 1a and (**b**) Parameter Set 1b.

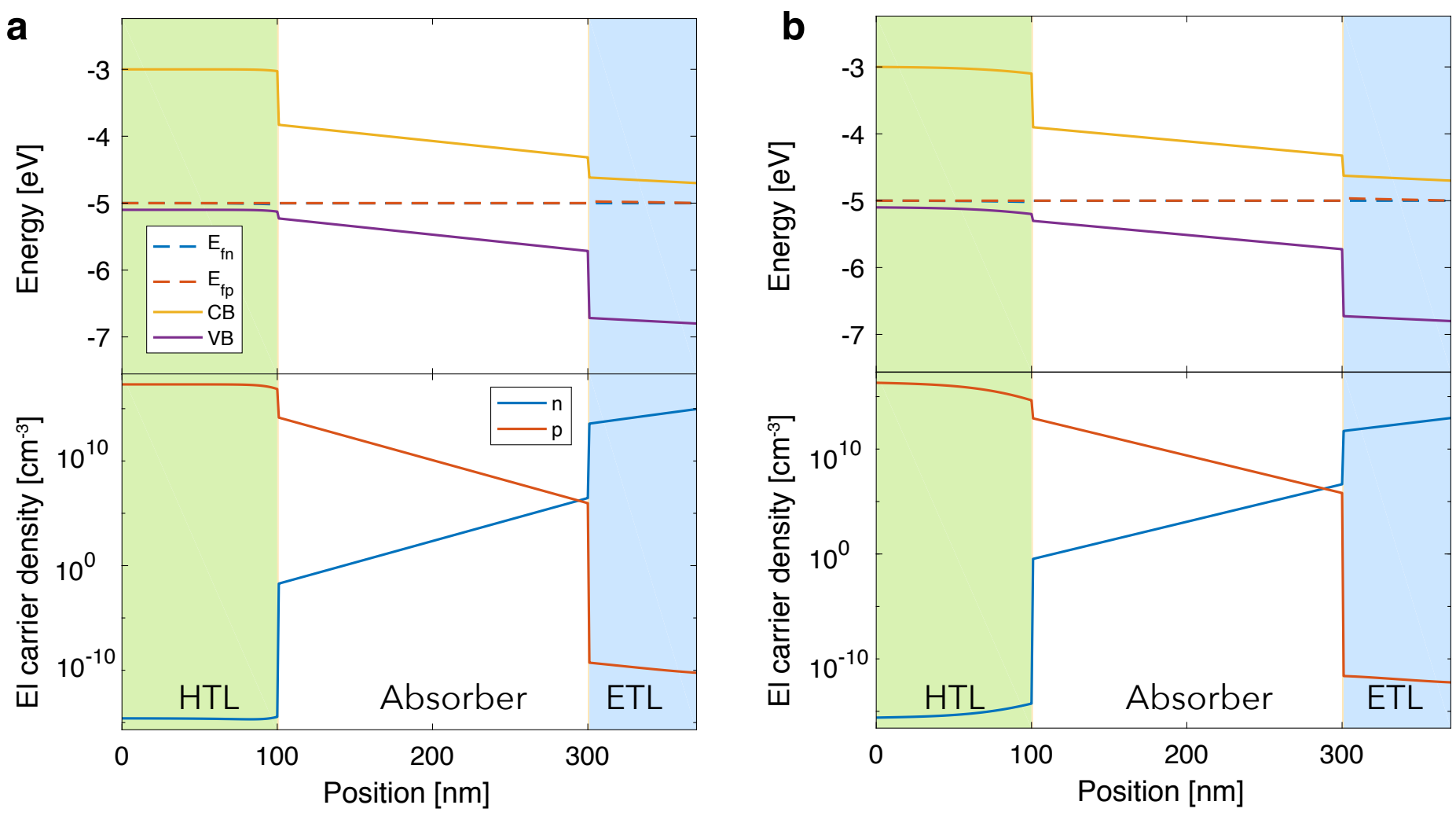

Fig. S.13 Equilibrium energy level diagrams and carrier densities for Parameter Sets 2a and 2b. Equilibrium states for (**a**) Parameter Set 2a and (**b**) Parameter Set 2b.

S.9.3.5 Additional data

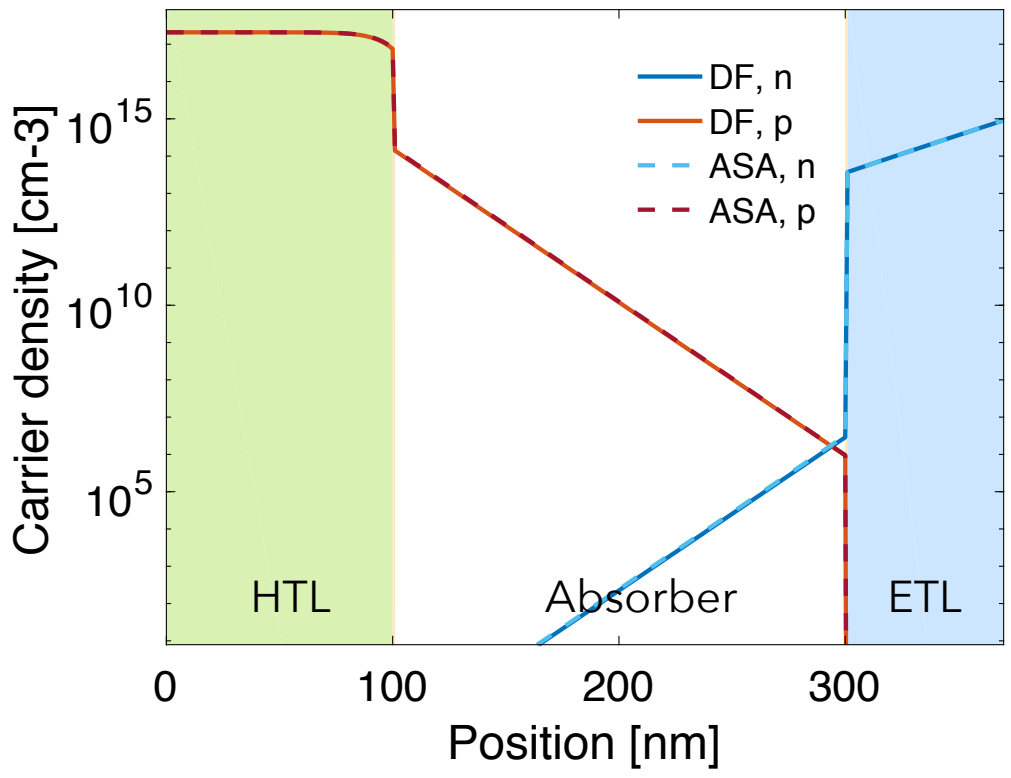

Fig. S.14 Carrier densities at equilibrium for a simulated device comparing Driftfusion with ASA using parameter set 2. Results from Driftfusion are indicated by solid curves whereas results from ASA are indicated by dashed curves.

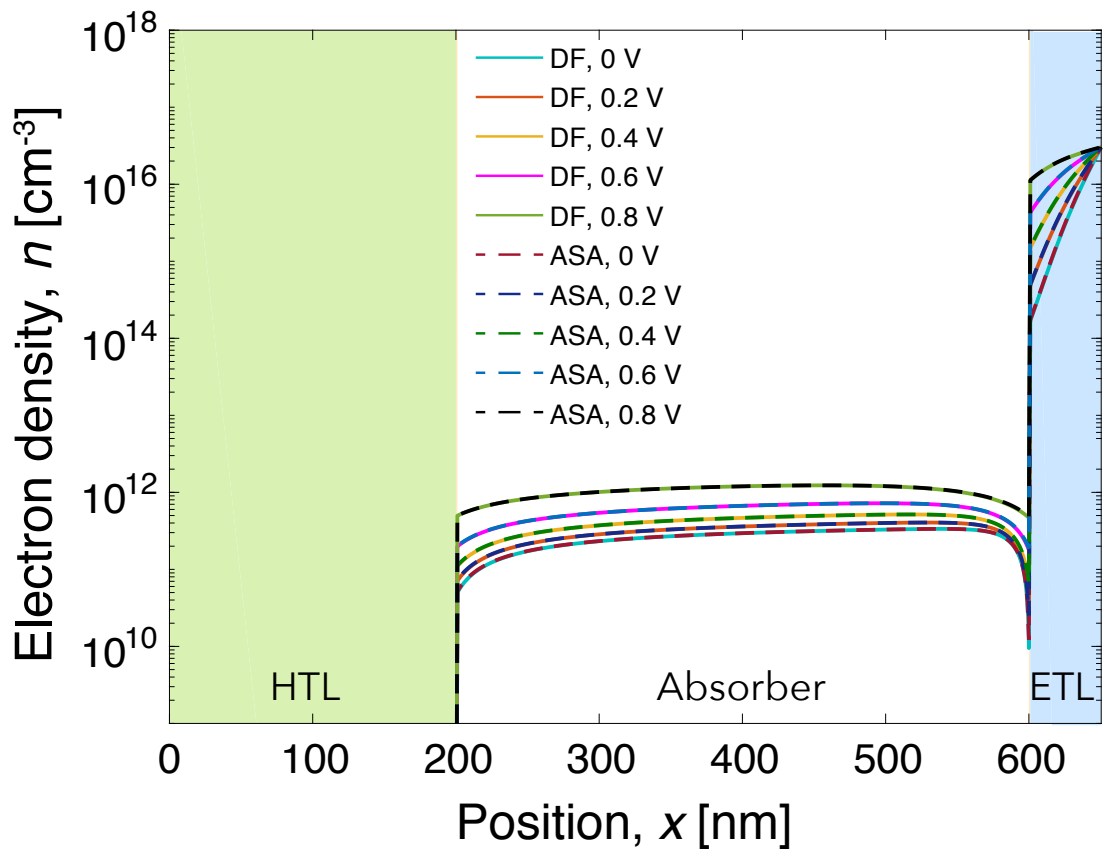

Fig. S.15 Comparison of calculated Driftfusion and ASA electron density profiles during *J*-*V* scan for a three-layer device with Parameter Set 1 under illumination.

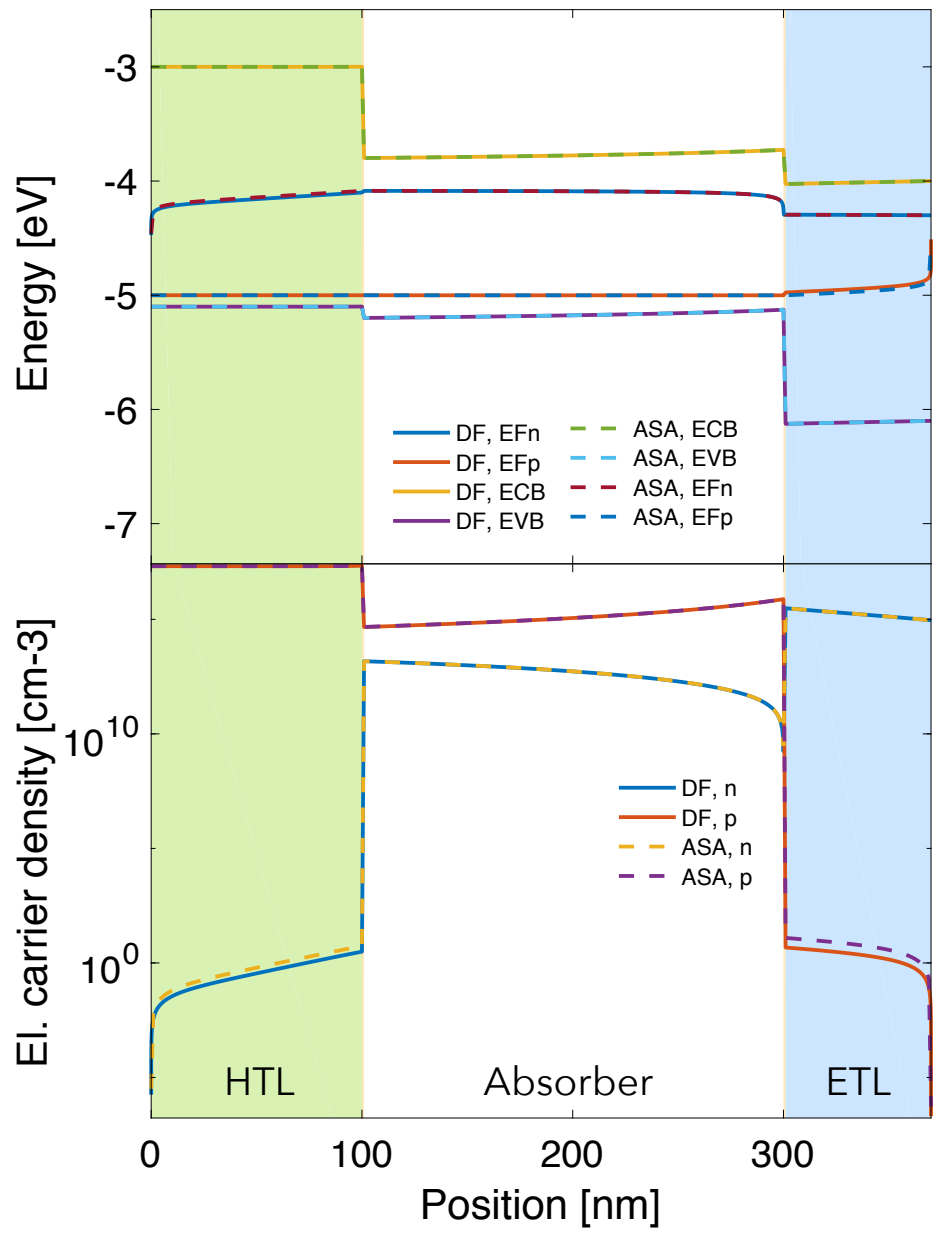

Fig. S.16 Energy level diagram and carrier densities for a device with properties defined by Parameter Set 2 under illumination at Vapp = 0.7 V. Results from Driftfusion indicated by the solid lines, while those from ASA are indicated by dashed lines.

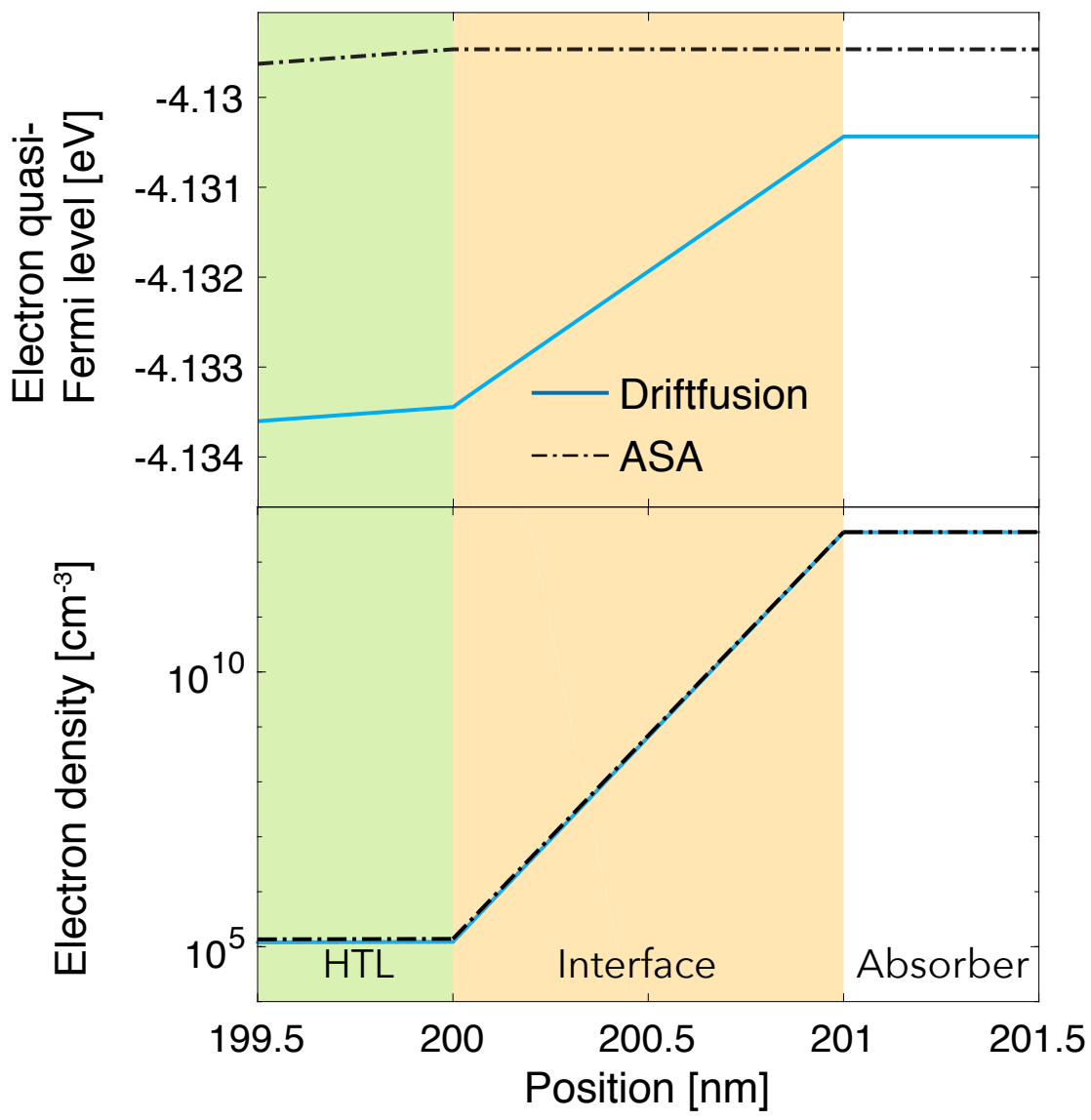

Fig. S.17 Electron quasi-Fermi level and carrier density under illumination at Vapp = 1 V comparison between Driftfusion and ASA. Results from Driftfusion are indicated by solid blue curves whereas results from ASA are indicated by dashed black curves.

S.9.4 Numerical solution for a three-layer device with mobile ions: comparison with IonMonger

Repository branch:	`Methods_IonMonger_comparison`
Parameters files:	`./Input_files/IonMonger_default_bulk.csv`
	`./Input_files/IonMonger_default_IR.csv`
Scripts:	`df_methods_IonMonger_comparison_bulk.m`
	`df_methods_IonMonger_comparison_IR.m`

Table S.13 Key layer-specific parameters for simulation comparison with IonMonger. *Ions in these layer are immobile and are balanced by a static counter charge hence they do not contribute to mobile ionic space charge. †The limiting ion densities have been set to a very high value since IonMonger does not include a steric model similar to that described in Section 3.4. ‡ Minority carriers are not simulated in the transport layers in IonMonger, hence recombination was effectively switched off in these regions. HTL and ETL denote hole and electron transport layers respectively.

Variable	Symbol	Layer			Unit
		HTL	Absorber	ETL	
Optical parameters material reference	-	SiO_2	MAPICl	SiO_2	-
Thickness	d	1×10^{-5}	4×10^{-5}	2×10^{-5}	cm
Electron affinity	Φ_{EA}	-3.1	-3.7	-4.0	eV
Ionisation potential	Φ_{IP}	-5.1	-5.4	-6.0	eV
Equilibrium Fermi energy	E_{F0}	-5.0	-4.55	-4.1	eV
Acceptor density	N_A	1.03×10^{18}	8.32×10^{7}	0	cm^{-3}
Donor density	N_D	0	0	1.03×10^{18}	cm^{-3}
SRH trap energy	E_{trap}	-4.1	-4.55	-5.0	eV
eDOS conduction band	N_{CB}	5×10^{19}	8.1×10^{18}	5×10^{19}	cm^{-3}
eDOS valence band	N_{VB}	5×10^{19}	5.8×10^{18}	5×10^{19}	cm^{-3}
Intrinsic cation density	N_{cat}	$5\times10^{19*}$	5×10^{19}	$5\times10^{19*}$	cm^{-3}
Intrinsic anion density	N_{ani}	$5\times10^{19*}$	$5\times10^{19*}$	$5\times110^{19*}$	cm^{-3}
Limiting cation density	c_{max}	$10^{100\dagger}$	$10^{100\dagger}$	$10^{100\dagger}$	cm^{-3}
Limiting anion density	a_{max}	$10^{100\dagger}$	$10^{100\dagger}$	$10^{100\dagger}$	cm^{-3}
Electron mobility	μ_e	0.2	20	0.1	$cm^2\ V^{-1}\ s^{-1}$
Hole mobility	μ_h	0.02	2	0.001	$cm^2\ V^{-1}\ s^{-1}$
Cation mobility	μ_c	$0^{\ddagger}$	3.12×10^{-12}	$0^{\ddagger}$	$cm^2\ V^{-1}\ s^{-1}$
Anion mobility	μ_a	$0^{\ddagger}$	$0^{\ddagger}$	$0^{\ddagger}$	$cm^2\ V^{-1}\ s^{-1}$
Relative dielectric constant	ε_r	3	24.1	10	-
Direct recombination coefficient	k_{rad}	$0^{\ddagger}$	1×10^{-12}	$0^{\ddagger}$	cm^3s^{-1}
SRH recombination electron lifetime	τ_n	$10^{100\ddagger}$	3×10^{-10}	$10^{100\ddagger}$	s
SRH recombination hole lifetime	τ_p	$10^{100\ddagger}$	3×10^{-8}	$10^{100\ddagger}$	s

Table S.14 Interface-specific parameters for simulation comparisons with IonMonger. (**a**) and (**b**) denote the bulk and interfacial recombination dominated devices respectively.* The relative dielectric constant is set artificially high in this parameter set to reduce the electric field strength within the interfacial regions: this modification facilitates a more direct comparison with the abrupt interface model.

Variable	Symbol	HTL-Absorber	ETL-Absorber	Unit
Thickness	d	10^{-7}	10^{-7}	cm
Electron surface recombination velocity	s_n	(**a**) 10^{-20} (**b**) 10	(**a**) 10^{-20} (**b**) 10^{7}	cm s^{-1}
Hole surface recombination velocity	s_p	(**a**) 10^{-20} (**b**) 10^{7}	(**a**) 10^{-20} (**b**) 10^{3}	cm s^{-1}
Relative dielectric constant	ε_r	10^{3}*	10^{3}*	-

Table S.15 Key device-wide parameters for simulation comparisons with IonMonger.

Variable	Symbol	Unit	
Left-hand boundary Fermi energy	Φ_l	−4.1	eV
Right-hand boundary Fermi energy	Φ_r	−5.0	eV
Left-hand boundary electron extraction coefficient	$s_{n,l}$	10^{7}	cm s^{-1}
Right-hand boundary electron extraction coefficient	$s_{n,r}$	0	cm s^{-1}
Left-hand boundary hole extraction coefficient	$s_{p,l}$	0	cm s^{-1}
Right-hand boundary hole extraction coefficient	$s_{p,r}$	10^{7}	cm s^{-1}

S.9.4.1 Equilibrium state

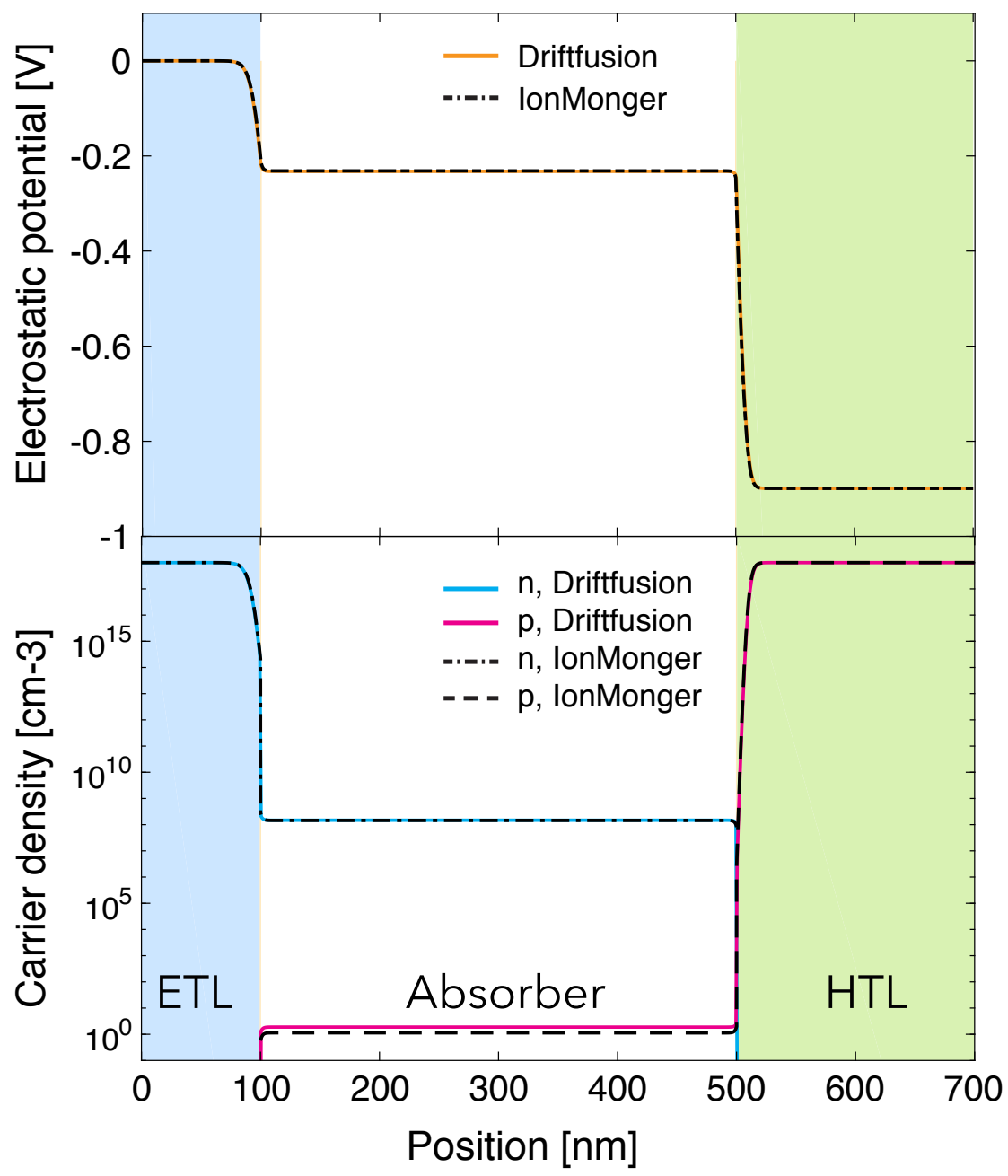

Fig. S.18 Comparison of the equilibrium state electrostatic potential and electronic carrier densities of the bulk carrier recombination device calculated by Driftfusion and IonMonger. Results from Driftfusion (DF) are indicated by solid coloured lines, whereas those from IonMonger (IM) are indicated by dashed black lines. Results for the device dominated by interfacial recombination (not shown) were very similar.

S.9.4.2 Additional data

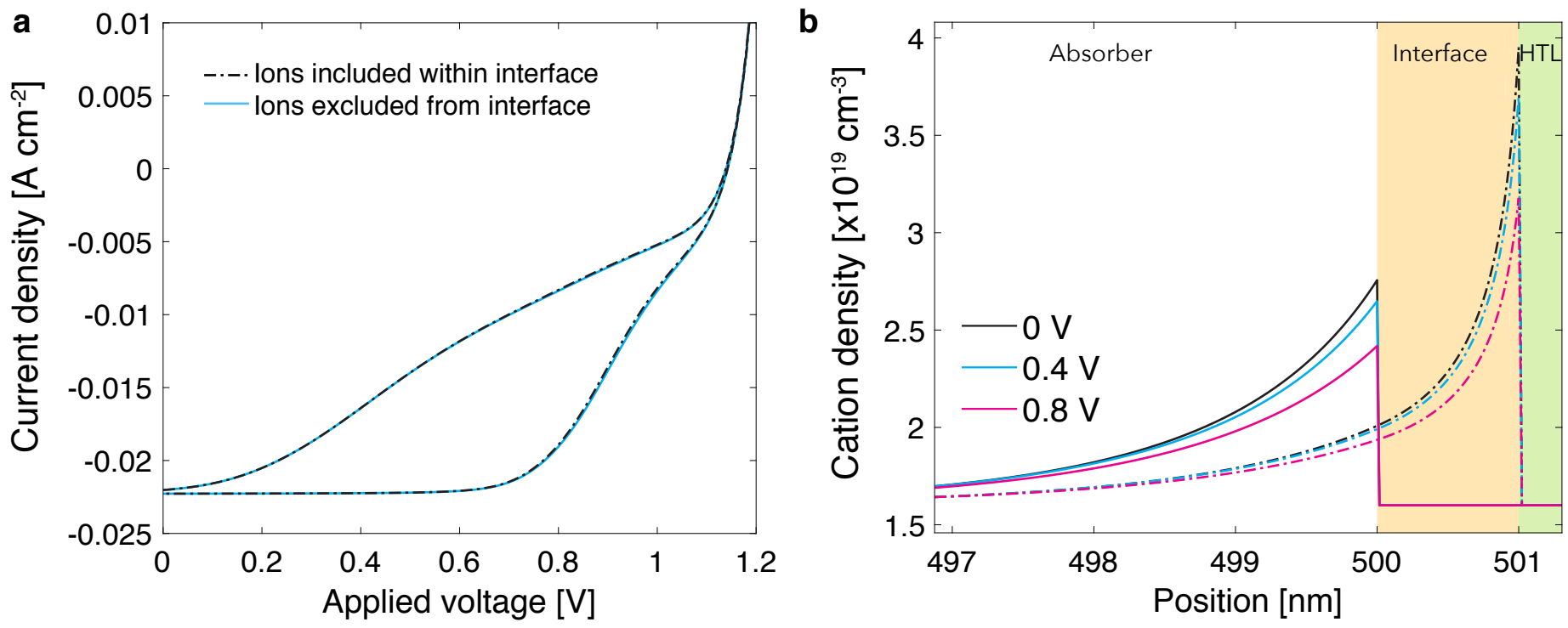

Fig. S.19 Comparison of current voltage characteristics and ionic carrier density profiles for a three-layer device calculated with Driftfusion. (**a**) Current-voltage scans for the three-layer device described by the parameters given in Tables S.13 and S.15 at a scan rate of 1 Vs^{-1}. (**b**) Corresponding ionic carrier densities for the two conditions at increasing applied voltage. Solid lines indicate calculations where ions were excluded, whilst dashed lines indicate cases where ions were included in the interface regions (yellow background). The green background indicates the hole transport layer.

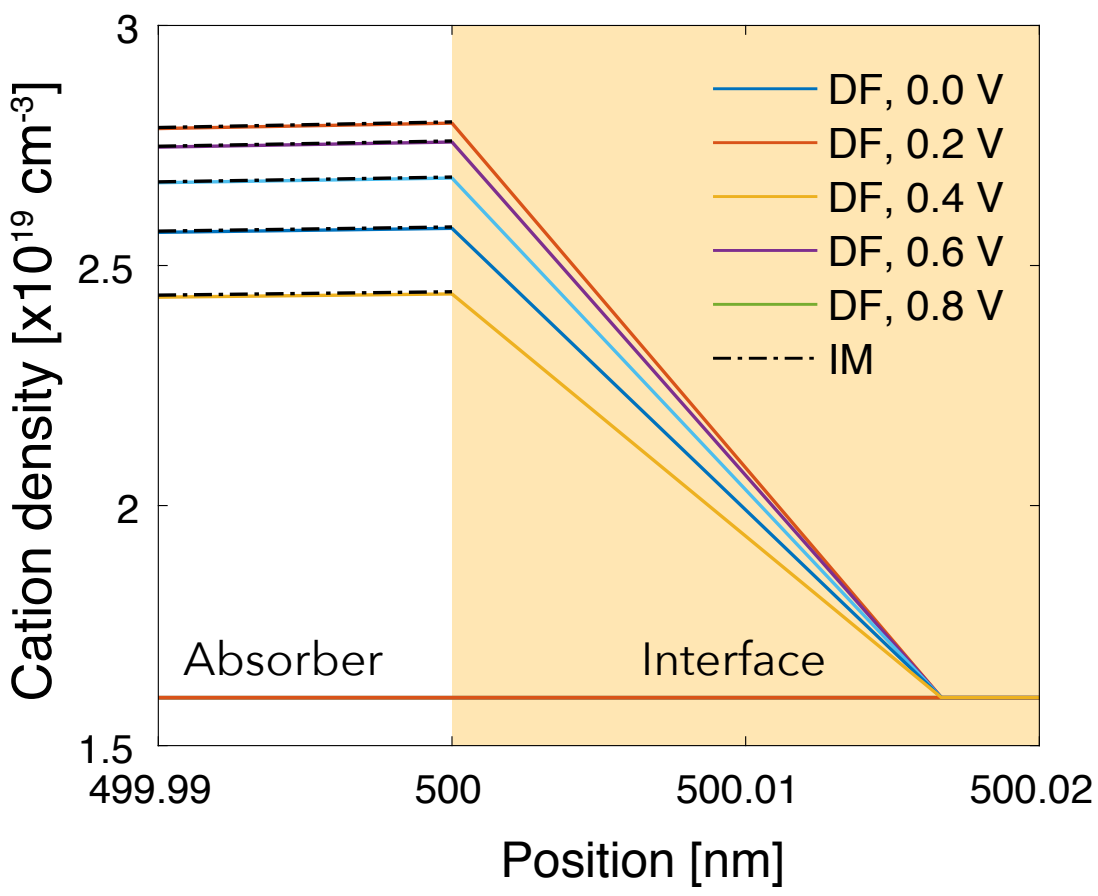

Fig. S.20 Zoom of ionic carrier density profiles for a three-layer device calculated with Driftfusion. Results from Driftfusion (DF) are indicated by solid coloured lines, whereas those from IonMonger (IM)are indicated by dashed black lines.

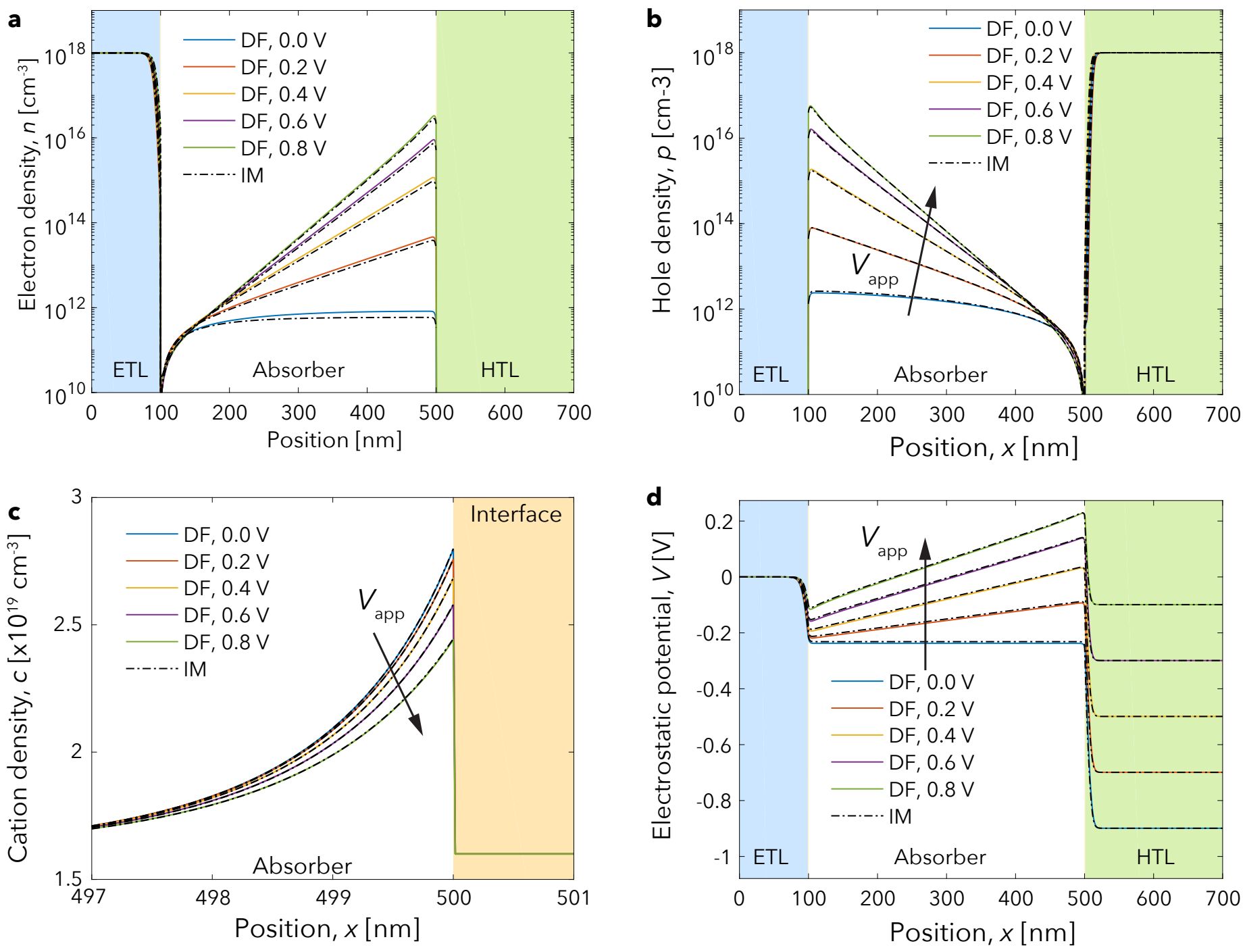

Fig. S.21 Comparison of results calculated using Driftfusion and IonMonger for a three-layer solar cell dominated by bulk recombination including mobile ionic carriers in the absorber layer. (**a**) Electron density (**b**) Hole density, (**c**) Cation density, and (**d**) electrostatic potential profiles at increasing voltage during a 1 V s^{-1} forward scan (c.f. Figure 16). Results from Driftfusion (DF) are indicated by solid coloured lines, whereas those from IonMonger (IM) are indicated by dashed black lines. The complete parameter sets for the simulations are given in Tables S.13, S.14 and S.15.

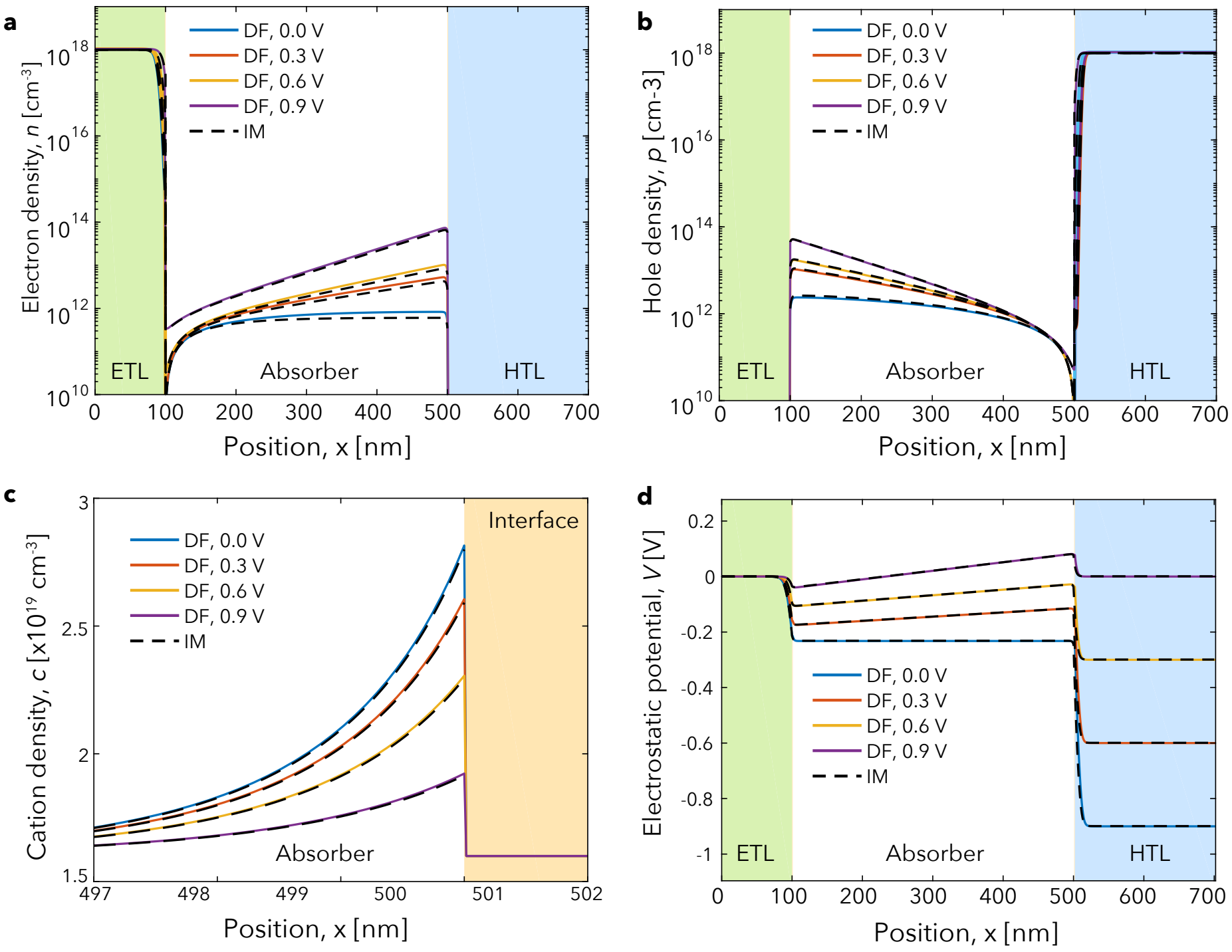

Fig. S.22 Comparison of results calculated using Driftfusion and IonMonger for a three-layer solar cell dominated by interfacial recombination including mobile ionic carriers in the absorber layer. (**a**) Electron density (**b**) Hole density, (**c**) Cation density, and (**d**) electrostatic potential profiles at increasing voltage during a 100 mV s^{-1} forward scan (c.f. Figure 16). Results from Driftfusion (DF) are indicated by solid coloured lines, whereas those from IonMonger (IM) are indicated by dashed black lines. The complete parameter sets for the simulations are given in Tables S.13, S.14 and S.15.

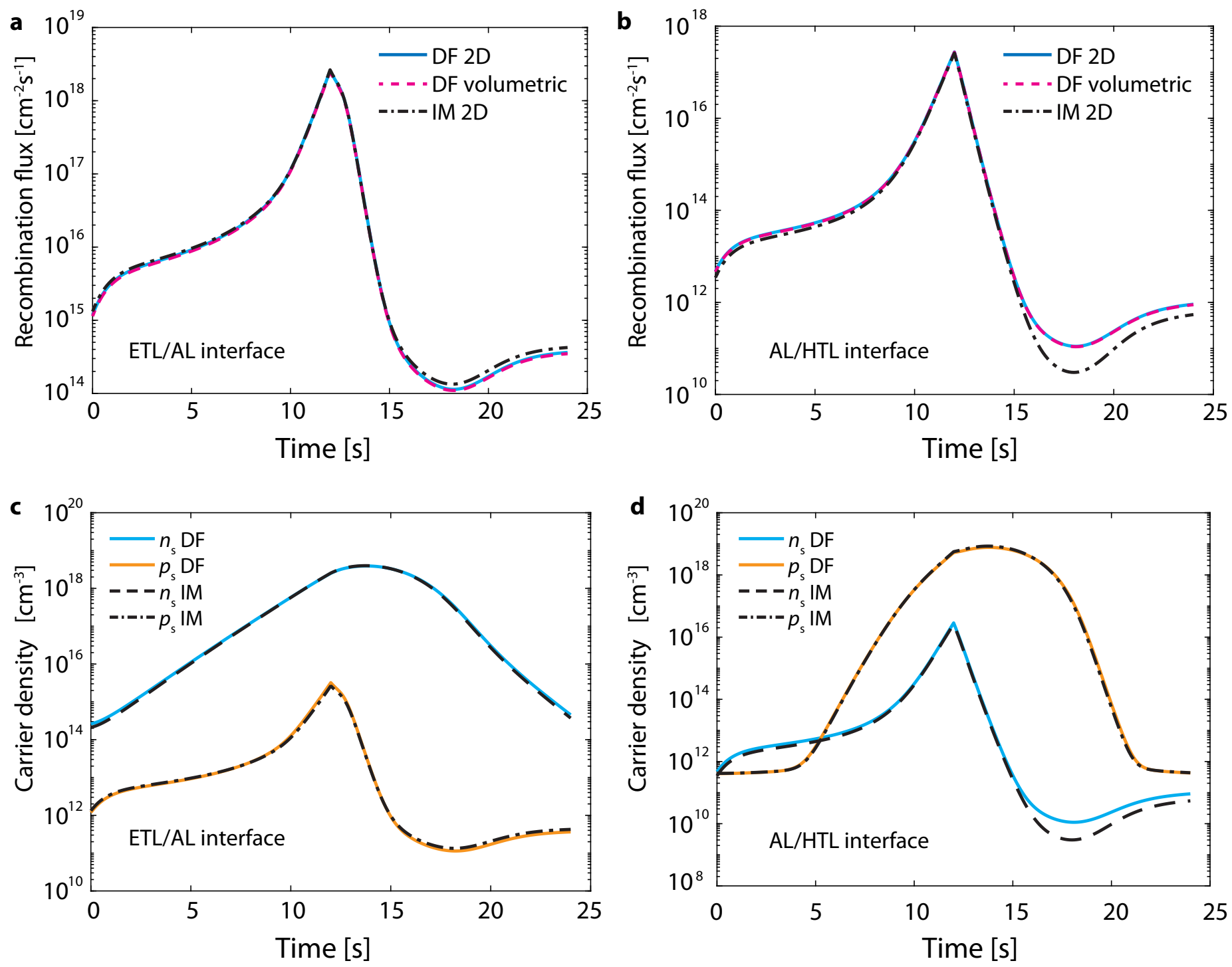

Fig. S.23 Comparison of interfacial recombination fluxes and interface surface carrier densities as a function of time during a 100 mV *J*-*V* scan calculated using Driftfusion and IonMonger for a three-layer solar cell dominated by interfacial recombination and including mobile ionic carriers in the absorber layer. Recombination fluxes for (**a**) the electron transport layer - active layer (ETL/AL) interface and (**b**) the active layer - hole transport layer interface (AL/HTL). (**c**) and (**d**): Corresponding interfacial surface carrier densities n_s and p_s (see Figure 4 for illustration) for the ETL/AL and AL/HTL interfaces respectively. The two dimensional recombination flux for Driftfusion (DF 2D, solid coloured curves) have been back-calculated from the solution using n_s and p_s and the SRH surface recombination expression in Equation 46. The actual recombination fluxes calculated in Driftfusion, using the volumetric scheme described in Section 3.7.3 are labelled as DF volumetric (dashed coloured curves). The recombination fluxes calculated using IonMonger[8] are also back-calculated using the expression in Ref [9], where additional factors are included with the n_i^2, which have been neglected here. The volumetric interfacial recombination scheme in Driftfusion shows a high level of self-consistency with the 2-D model. Some variation is observed between Driftfusion and IonMonger, which can be attributed to the difference in electron density at the AL/HTL interface owing to small differences in the ionic carrier distributions shown in Figure S.22.

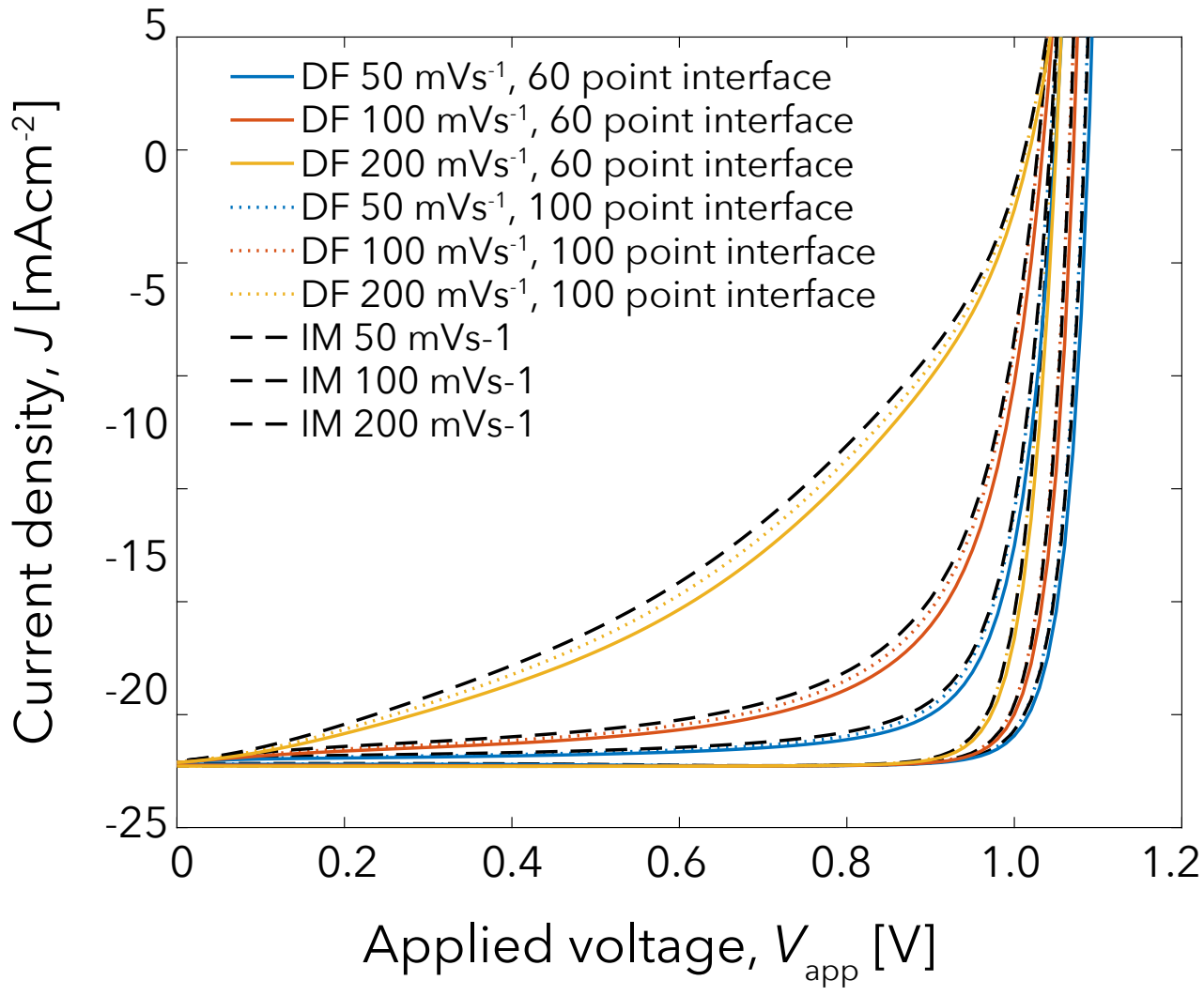

Fig. S.24 Effect of change in band energy and mesh spacing on accuracy of the volumetric surface recombination model currents. Current density-voltage scans for devices with the parameter set given in Table S.13 with energetic barriers to minority carriers of 0.8 eV as opposed to 0.4 eV for both transport layers. The larger values of α and β (Main Text, Equations 37 and 38) for the 0.8 eV barriers (solid curves) lead to a larger divergence from the abrupt interface model (black dashed curves) than the 0.4 eV barriers shown in Figure 16 of the Main Text. The accuracy of the volumetric recombination scheme can be improved by doubling the interface thickness to 2 nm (thus reducing α and β) and increasing the number of points in the interfaces from 60 to 100 (Coloured, dotted curves).

References

1. P. Calado, P.R.F. Barnes, I. Gelmetti, M. Azzouzi, B. Hilton. Driftfusion (2017). DOI 10.5281/zenodo.3670155. URL `https://github.com/barnesgroupICL/Driftfusion`
2. D.L. Scharfetter, H.K. Gummel, IEEE Transactions on Electron Devices **ED-16**(1) (1969). DOI 10.1109/T-ED.1969.16566
3. P. Farrell, N. Rotundo, D.H. Doan, M. Kantner, J. Fuhrmann, T. Koprucki, Numerical methods for drift-diffusion models. Tech. rep., Leibniz-Institut im Forschungsverbund Berlin, Berlin (2016)
4. NREL. Reference Solar Spectral Irradiance: ASTM G-173 (2017). URL `https://www.nrel.gov/grid/solar-resource/spectra.html`
5. P. Calado, Transient optoelectronic characterisation and simulation of perovskite solar cells. Ph.D. thesis, Imperial College London (2017). URL `https://spiral.imperial.ac.uk/handle/10044/1/66894`
6. J. Nelson, *The physics of solar cells* (Imperial College Press, 2003)
7. C.G. Shuttle, Recombination dynamics in polythiophene: fullerene solar cells. Ph.D. thesis, Imperial College London (2008)
8. N.E. Courtier, J.M. Cave, A.B. Walker, G. Richardson, J.M. Foster, Journal of Computational Electronics **18**(4), 1435 (2019). DOI 10.1007/s10825-019-01396-2
9. N.E. Courtier, J.M. Cave, J.M. Foster, A.B. Walker, G. Richardson, Energy & Environmental Science **12**(1), 396 (2019). DOI 10.1039/C8EE01576G
